# Supplementary material for: Estimating the basic reproduction number at the beginning of an outbreak
Source: PLoS One. 2022 Jun 17;17(6):e0269306. doi: 10.1371/journal.pone.0269306 (PMC9205483; doi:10.1371/journal.pone.0269306)
Supplement: S1 File — (PDF) [file pone.0269306.s001.pdf]

Supplementary Information for "Estimating the basic reproduction number at the beginning of an outbreak"

Sawitree Boonpatcharanon[1], Jane Heffernan[2,3], Hanna Jankowski[2,3]

[1] Statistics, Chulalongkorn Business School, Chulalongkorn University, Thailand
[2] Mathematics & Statistics, York University, Toronto, Canada
[3] Centre for Disease Modelling, York University, Toronto, Canada

April 18, 2022

1 Introduction

Our study centres on estimating R_0 using six R_0 estimator methods from the literature (see Table 1). We consider well-specified and misspecified cases, whereby the estimator and the model follow the same epidemic structure (well-specified), the estimator and the model do not have the same epidemic structure (misspecified) or the estimator is provided information that is incorrect (misspecified). Well-specified (WS) and misspecified (MS) cases are indicated in Section Background Information.

We provide plots of the mean squared error (MSE) of the estimated R_0 value to the known R_0 value. These are accompanied by side-by-side box plots of the R_0 estimates for each estimator method, for the well-specified and misspecified cases. Finally, we also report tables of R_0 values for each method, with standard deviation (SD). Again, values are reported for the well-specified and misspecified cases.

In the tables, the median is reported in the top row of each column. This is followed by the SD in the next row. SD values greater than one million are reported as *. SD values of zero reflect SD values that are so small that they round to zero.

Some estimators can return NA, with certain numerical combinations, for example, when there are two zero values in two consecutive weeks of data.. In these cases, we report the number of simulations that yield NA. If there is no NA count row in the table, then the method did not yield any NA values.

1.1 Background Information

In our study we employ six methods to estimate R_0 from epidemic data. The estimators are described in Table 1. More detail on each estimator method is provided in the main text of this study.

We consider synthetic and real world epidemic data. The real world data includes reported case counts from the early weeks of the Canadian COVID-19 pandemic, from January 25, 2020 to the implementation of lockdowns in mid-March. We consider case count data for the entire Canadian population, and the three largest province, by population, the provinces of Ontario (ON), Quebec (QC), and British Columbia (BC).

The synthetic data are generated from epidemic agent-based models that follow

SIR: **S**usceptible–**I**nfectious–**R**ecovered

SEIR: **S**usceptible–**E**xposed–**I**nfectious–**R**ecovered

Table 1: Summary of estimation methods for R_0.

method	summary
WP	White & Pagano Method, due to (1). Serial distribution can be assumed known or can be estimated using MLE; method developed under branching process model; simple method which yields real-time estimates (when serial interval is unknown the method takes longer to compute).
seqB	Sequential Bayes Method, due to (2). Serial distribution assumed known (only the mean is used); method developed assuming SIR model and uses sequential Bayes methods; simple method which yields real-time estimates.
ID	Incidence Decay Method. Serial distribution assumed known (only the mean is used); method developed assuming an SIR model structure and uses least squares estimation. It is a simple method which yields real-time estimates.
IDEA	The Incidence Decay and Exponential Adjustment Method is presented in (3). Serial distribution assumed known (only the mean is used); method developed assuming SIR model and uses least squares estimation; simple method which yields real-time estimates. IDEA uses a slightly more complex model for fitting than ID.
plug-and-play	Plug-and-Play Method. See (4). Serial distribution assumed unknown; method selects one of SIR/-SEIR/SEAIR model; implementations available though not real-time (depending on input selection). Generally, this approach fits the complete model using maximum likelihood and relying on Monte Carlo to fill in missing observations.
fullBayes	Full Bayes Method. See (5). Serial distribution assumed unknown; method selects one of SIR/SEIR/SEAIR model; not real-time. this approach fits the complete model using maximum likelihood and relying on Monte Carlo to fill in missing observations.

SEAIR: **S**usceptible–**E**xposed–(**A**symptomatic Infectious)–(Symptomatic **I**nfectious)–**R**ecovered

epidemic structures. These models for the basis of all epidemiological models of infection diseases (6; 7; 8). The models are each composed of three to five compartments (with labels matching the model name). Individuals transition from one compartment to the next based on prespecified random dynamics. Here, we assume that these distributions are exponential, and thus assume systems of ordinary differential equations (ODEs).

The mathematical model simulations are used to generate data for three different parameter sets that are representative of pandemic influenza (Influenza 1 and Influenza 2 parameter sets) and COVID-19. The parameter sets are provided in Table 2 for the SIR, SEIR, and SEAIR models, given the parameters that apply to each disease model structure. Model parameters were taken from the literature. Pandemic influenza

parameters sets were chosen such that $R_0 \in [1.2, 7]$ and the serial interval $\in [1.5, 9.5]$. The COVID-19 parameter set has $R_0 \in [1.6, 3.4]$ and serial interval $\in [4.2, 7.5]$ (9; 10; 11; 12). Influenza 1 parameters are such that $R_0 = 5/3$ for SIR and SEIR and $R_0 = 7/3$ for SEAIR. For this example, the serial interval is 5 days for the SIR model and 8 days for the SEIR and SEAIR models. Influenza 2 parameters are such that $R_0 = 5/3$ and the serial interval is 5 days for each of the SIR, SEIR, and SEAIR models. The COVID-19 parameters are such that $R_0 = 2.6$ and the serial interval is 5.2 days, again, for all models.

For each epidemic, the population size N is set to 10,001 where $S(0) = 10,000$ and $I(0) = 1$. We ignore demographics (births and deaths), and therefore have a constant size population. That is, for all $t \geq 0$ it holds that, for the SIR model, $S(t) + I(t) + R(t) = S_0 + I_0 = N$. Similarly, $S(t) + E(t) + I(t) + R(t) = S_0 + I_0 = N$ for the SEIR model, and $S(t) + E(t) + A(t) + I(t) + R(t) = S_0 + I_0 = N$ for the SEAIR model.

Using the synthetic data, we consider well-specified (WS) and misspecified (MS) cases for each estimator. These are indicated as WS and MS in the following list:

1. WP method assuming
   - WS - serial distribution (SD) is known and set to exponential with correct mean (5 days for influenza 1 and 2 and 5.2 days for COVID-19)
   - MS - SD is known and set to exponential with incorrect mean (3 days for influenza 1, 2 and 7 days for influenza 2, and 4.2 and 7.5 days for COVID-19)
   - WS but with room for error - SD is unknown and estimated from a gamma distribution with unknown mean and variance (using a grid search algorithm). The estimated SD is given by a series of values denoted by p_t.
   - Method is applied to SIR (WS), SEIR (MS), and SEAIR (MS) data
2. seqB method assuming
   - WS - SD has the correct mean (5 days for influenza 1 and 2 and 5.2 days for COVID-19)
   - MS - SD has an incorrect mean (3 days for influenza 1, 2 and 7 days for influenza 2, and 4.2 and 7.5 days for COVID-19)
   - Method is applied to SIR (WS), SEIR (MS), and SEAIR (MS) data
3. ID and IDEA methods assuming
   - WS - SD has the correct mean (5 days for influenza 1 and 2 and 5.2 days for COVID-19)
   - MS - SD has an incorrect mean (3 days for influenza 1, 2 and 7 days for influenza 2, and 4.2 and 7.5 days for COVID-19)
   - Method is applied to SIR (WS), SEIR (MS), and SEAIR (MS) data
4. plug-n-play and fullBayes methods developed assuming
   - All scenarios have SD unknown
   - SIR model applied to SIR (WS), SEIR (MS), and SEAIR (MS) data
   - SEIR model applied to SEIR (WS) SEAIR (MS) data
   - SEAIR model applied to SEAIR data only (WS)

For the real-world COVID-19 data, we assume serial intervals of 2, 5, and 8 days if a known serial interval is needed for the method to run.

Table 2: SIR, SEIR, SEAIR model parameter values

model	influenza 1	influenza 2	COVID-19
SIR (β, γ)	$(1/3, 1/5)$	$(1/3, 1/5)$	$(1/2, 5/26)$
SEIR (β, σ, γ)	$(1/3, 1/3, 1/5)$	$(5/9, 1/2, 1/3)$	$(13/11, 1/3, 5/11)$
SEAIR $(\beta, \sigma, \rho, \gamma)$	$(1/3, 1/3, 1/2, 1/5)$	$(5/12, 1/2, 1, 1/3)$	$(26/57, 1/3, 2/7, 5/11)$

2 Influenza 1 example

Parameter values are listed in Table 2. Influenza 1 parameters are such that $R_0 = 5/3$ for SIR and SEIR and $R_0 = 7/3$ for SEAIR. For this example, the serial interval is 5 days for the SIR model and 8 days for the SEIR and SEAIR models.

For the WP method, we obtain values of p_t, representing the serial distribution. The serial distribution values, discretized to weeks, from the SIR/SEIR/SEAIR model epidemic data for influenza 1 are

- 0.75 0.19 0.05 0.01
- 0.53 0.33 0.10 0.03 0.01
- 0.48 0.36 0.12 0.03 0.01 .

Given a known serial interval μ (in days), we obtain the following serial distributions, discretized to weeks:

- ($\mu = 5$) 0.75 0.19 0.05 0.01
- ($\mu = 2$) 0.97 0.03
- ($\mu = 8$) 0.58 0.24 0.10 0.04 0.02 0.01 .

When the serial distribution is unknown, the WP method determines serial distributions. fullBayes and plug-n-play also return estimates of the serial distribution, for the SIR, SEIR, and SEAIR examples. Estimates of the serial distribution are shown in Table 3. Note that for the WP method, we make the standard actuarial mid-week assumption. That is, we assume that within a week, an event occurs on average in the middle of the week.

Table 3: Influenza example 1 - estimating SI (value in weeks): WP, fullBayes, and plug-n-play methods applied to SIR, SEIR, and SEAIR data, with SI unknown. fullBayes and plug-n-play methods assume SIR, SEIR, or SEAIR model structures. Each row reports the median value. The true SI (in weeks) is for $5/7 = 0.71$ for SIR and $8/7 = 1.14$ for the SEIR/SEAIR models.

		Week														
	data	1	2	3	4	5	6	7	8	9	10	11	12	13	14	15
WP (SI unknown)	SIR	0.5<br>(28)	0.5<br>(4.3)	0.5<br>(5.8)	0.7<br>(5.2)	0.8<br>(4.9)	0.8<br>(5)	0.5<br>(5.1)	0.5<br>(4.9)	0.5<br>(3.8)	0.5<br>(3.1)	0.5<br>(4.5)	0.5<br>(2.8)	0.5<br>(0.3)	0.5<br>(0.5)	0.5<br>(1.8)
	SEIR	0.5<br>(23.4)	0.5<br>(4)	0.6<br>(5.3)	1<br>(4.7)	1<br>(4.4)	1.1<br>(4.3)	1.2<br>(4.3)	1.3<br>(3.9)	1.3<br>(3.6)	1.2<br>(3.3)	1<br>(3.2)	0.9<br>(3)	0.5<br>(2.8)	0.5<br>(2.4)	0.5<br>(2)
	SEAIR	0.5<br>(14.8)	0.5<br>(12.1)	1.8<br>(8.2)	1.9<br>(8.6)	2.5<br>(8.2)	3.6<br>(8.4)	3.1<br>(8.1)	1.8<br>(8.7)	0.9<br>(8.2)	0.5<br>(9)	0.5<br>(8.7)	0.5<br>(7.6)	0.5<br>(8.6)	0.5<br>(6.1)	0.5<br>(3)
plug-n-play (SIR)	SIR	0.6<br>(0.4)	0.6<br>(0.4)	0.6<br>(0.4)	0.6<br>(0.4)	0.6<br>(0.4)	0.6<br>(0.4)	0.6<br>(0.4)	0.6<br>(0.4)	0.6<br>(0.4)	0.6<br>(0.4)	0.6<br>(0.4)	0.6<br>(0.4)	0.6<br>(0.4)	0.6<br>(0.4)	0.6<br>(0.4)
	SEIR	0.7<br>(0.3)	0.8<br>(0.3)	0.7<br>(0.3)	0.7<br>(0.3)	0.7<br>(0.3)	0.7<br>(0.3)	0.7<br>(0.3)	0.7<br>(0.3)	0.7<br>(0.3)	0.7<br>(0.3)	0.7<br>(0.3)	0.7<br>(0.3)	0.7<br>(0.3)	0.7<br>(0.3)	0.7<br>(0.3)
	SEAIR	0.7<br>(0.3)	0.7<br>(0.3)	0.7<br>(0.3)	0.8<br>(0.3)	0.7<br>(0.3)	0.7<br>(0.3)	0.7<br>(0.3)	0.7<br>(0.3)	0.7<br>(0.3)	0.7<br>(0.3)	0.7<br>(0.3)	0.7<br>(0.3)	0.7<br>(0.3)	0.7<br>(0.3)	0.7<br>(0.3)
plug-n-play (SEIR)	SEIR	1.2<br>(0.3)	1.2<br>(0.3)	1.2<br>(0.3)	1.2<br>(0.3)	1.2<br>(0.3)	1.2<br>(0.3)	1.2<br>(0.3)	1.2<br>(0.3)	1.2<br>(0.3)	1.2<br>(0.3)	1.2<br>(0.3)	1.2<br>(0.3)	1.2<br>(0.3)	1.2<br>(0.4)	1.2<br>(0.3)
	SEAIR	1.2<br>(0.3)	1.2<br>(0.3)	1.2<br>(0.3)	1.2<br>(0.3)	1.2<br>(0.3)	1.2<br>(0.3)	1.2<br>(0.3)	1.2<br>(0.3)	1.2<br>(0.3)	1.2<br>(0.3)	1.2<br>(0.3)	1.2<br>(0.3)	1.2<br>(0.3)	1.2<br>(0.3)	1.2<br>(0.3)
plug-n-play (SEAIR)	SEAIR	1.2<br>(0.3)	1.2<br>(0.3)	1.2<br>(0.3)	1.2<br>(0.3)	1.2<br>(0.3)	1.2<br>(0.3)	1.2<br>(0.3)	1.2<br>(0.3)	1.2<br>(0.3)	1.2<br>(0.3)	1.2<br>(0.4)	1.2<br>(0.3)	1.2<br>(0.3)	1.2<br>(0.4)	1.2<br>(0.3)
fullBayes (SIR)	SIR	8.2<br>(5.9)	2.4<br>(1)	2<br>(0.4)	1.9<br>(0.3)	1.7<br>(0.2)	1.6<br>(0.2)	1.5<br>(0.2)	1.3<br>(0.2)	1.2<br>(0.2)	1.1<br>(0.1)	1.1<br>(0.1)	1<br>(0.1)	1<br>(0.1)	1<br>(0)	1<br>(0)
	SEIR	3.9<br>(2)	2.3<br>(0.8)	1.9<br>(0.4)	1.7<br>(0.3)	1.6<br>(0.2)	1.6<br>(0.2)	1.5<br>(0.1)	1.4<br>(0.1)	1.4<br>(0.1)	1.3<br>(0.1)	1.3<br>(0.1)	1.3<br>(0.1)	1.2<br>(0.1)	1.2<br>(0.1)	1.1<br>(0.1)
	SEAIR	2.4<br>(1.7)	2.7<br>(1.1)	2.1<br>(0.5)	2<br>(0.3)	1.9<br>(0.3)	1.8<br>(0.2)	1.7<br>(0.1)	1.6<br>(0.1)	1.5<br>(0.1)	1.4<br>(0.1)	1.2<br>(0.2)	1.1<br>(0.1)	1.1<br>(0.1)	1<br>(0.1)	1<br>(0.1)
fullBayes (SEIR)	SEIR	5.7<br>(2.2)	3.2<br>(0.8)	2.9<br>(0.3)	3<br>(0.3)	3<br>(0.3)	3.1<br>(0.4)	3.2<br>(0.4)	3.3<br>(0.4)	3.3<br>(0.5)	3.4<br>(0.4)	3.5<br>(0.4)	3.6<br>(0.4)	3.7<br>(0.4)	3.9<br>(0.5)	4.1<br>(0.6)
	SEAIR	4.5<br>(1.8)	3.5<br>(1.2)	3.1<br>(0.5)	3<br>(0.3)	2.9<br>(0.3)	2.9<br>(0.3)	2.9<br>(0.2)	2.9<br>(0.2)	2.9<br>(0.2)	3<br>(0.2)	3.2<br>(0.3)	3.6<br>(0.5)	4.2<br>(0.7)	4.9<br>(0.9)	5.8<br>(1.1)
fullBayes (SEAIR)	SEAIR	2.2<br>(0.6)	2.1<br>(1.1)	1.5<br>(0.5)	1.2<br>(0.4)	1.2<br>(0.4)	1.3<br>(0.3)	1.4<br>(0.3)	1.4<br>(0.2)	1.4<br>(0.2)	1.4<br>(0.1)	1.4<br>(0.1)	1.4<br>(0.1)	1.3<br>(0.1)	1.3<br>(0.1)	1.3<br>(0.1)

2.1 MSE - Influenza Example 1

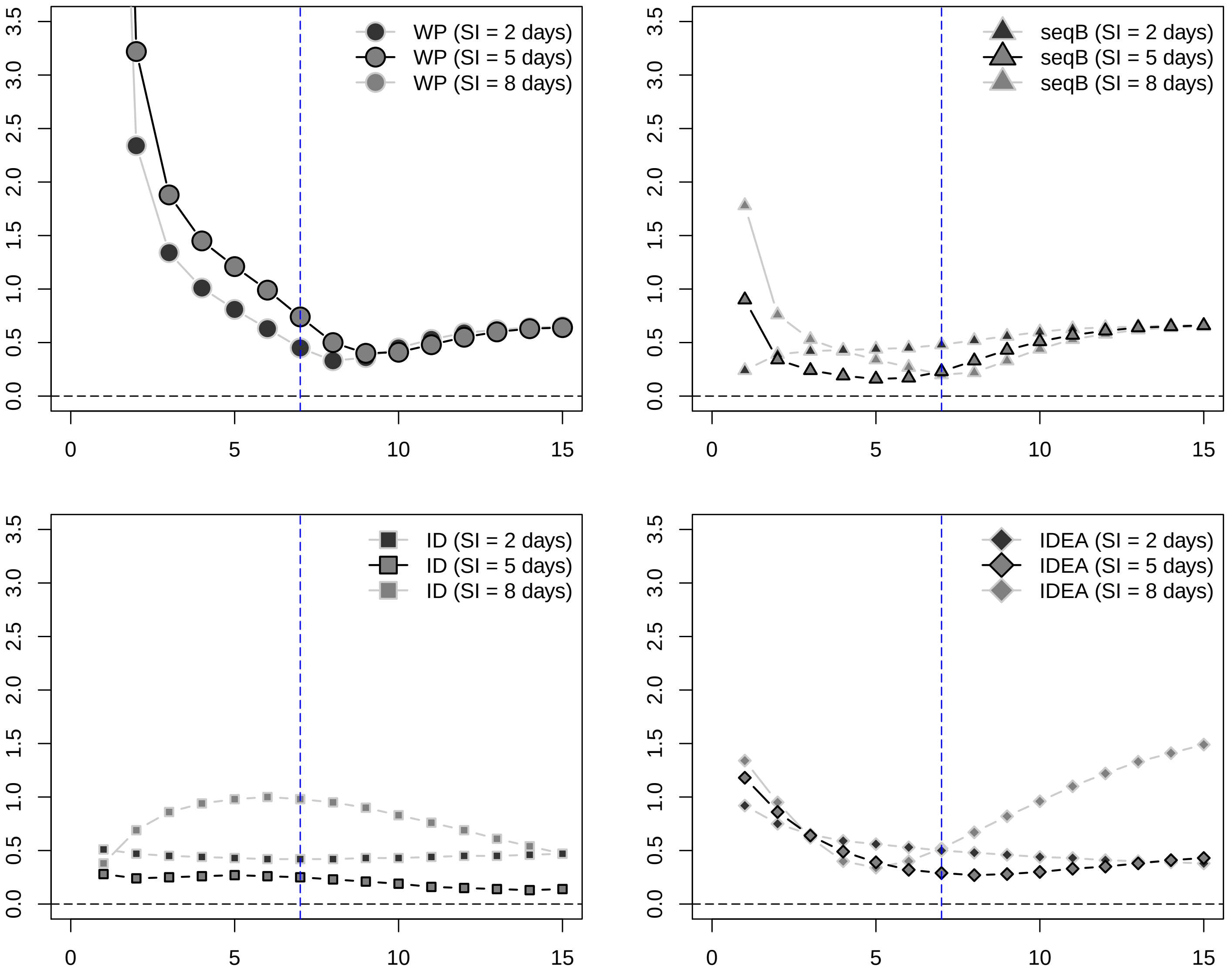

Figure 1: Influenza example 1 estimated MSE of WP, seqB, ID, and IDEA R_0 estimators assuming known SI with SIR data (week on x-axis) under well-specified and misspecified cases of the serial interval, where the true serial interval is 5 days. The inflection point related to the epidemic data curve of the SIR model is indicated by the blue dashed vertical line.

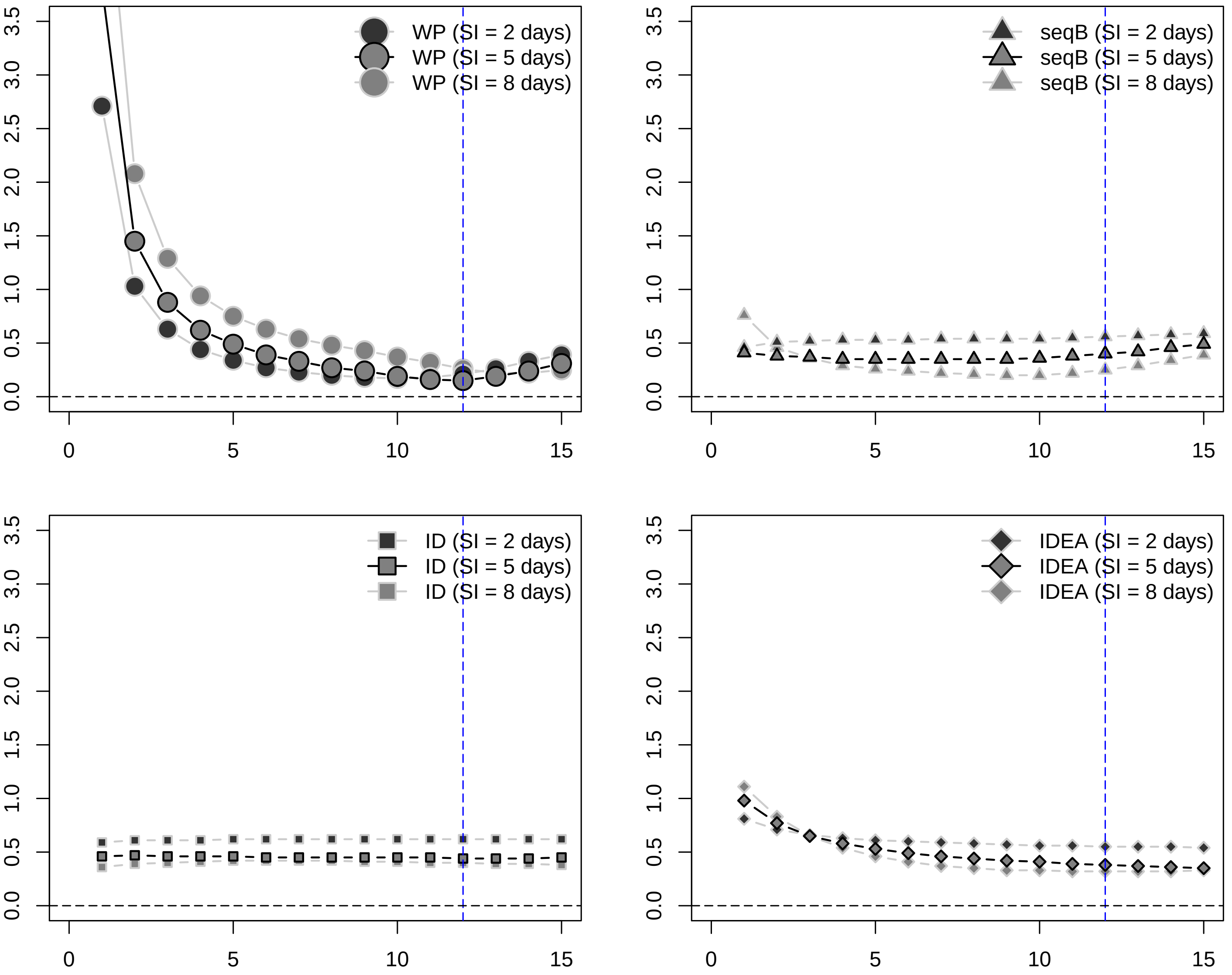

Figure 2: Influenza example 1 estimated MSE of WP, seqB, ID, and IDEA R_0 estimators assuming known SI with SEIR data (week on x-axis), where the true serial interval is 8 days. The inflection point related to the epidemic data curve of the SEIR model is indicated by the blue dashed vertical line.

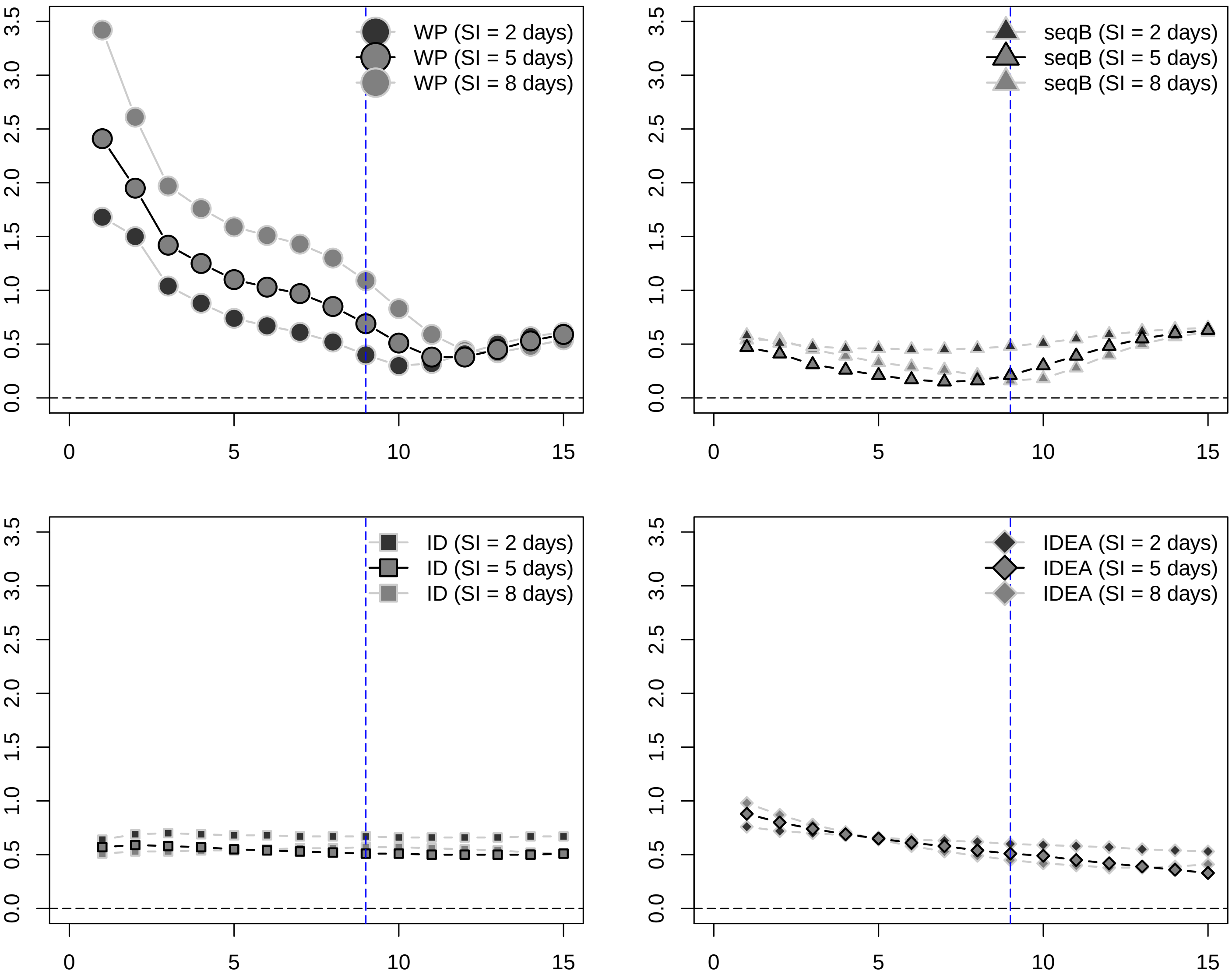

Figure 3: Influenza example 1 estimated MSE of WP, seqB, ID, and IDEA R_0 estimators assuming known SI with SEAIR data (week on x-axis), where the true serial interval is 8 days. The inflection point related to the epidemic data curve of the SEAIR model is indicated by the blue dashed vertical line.

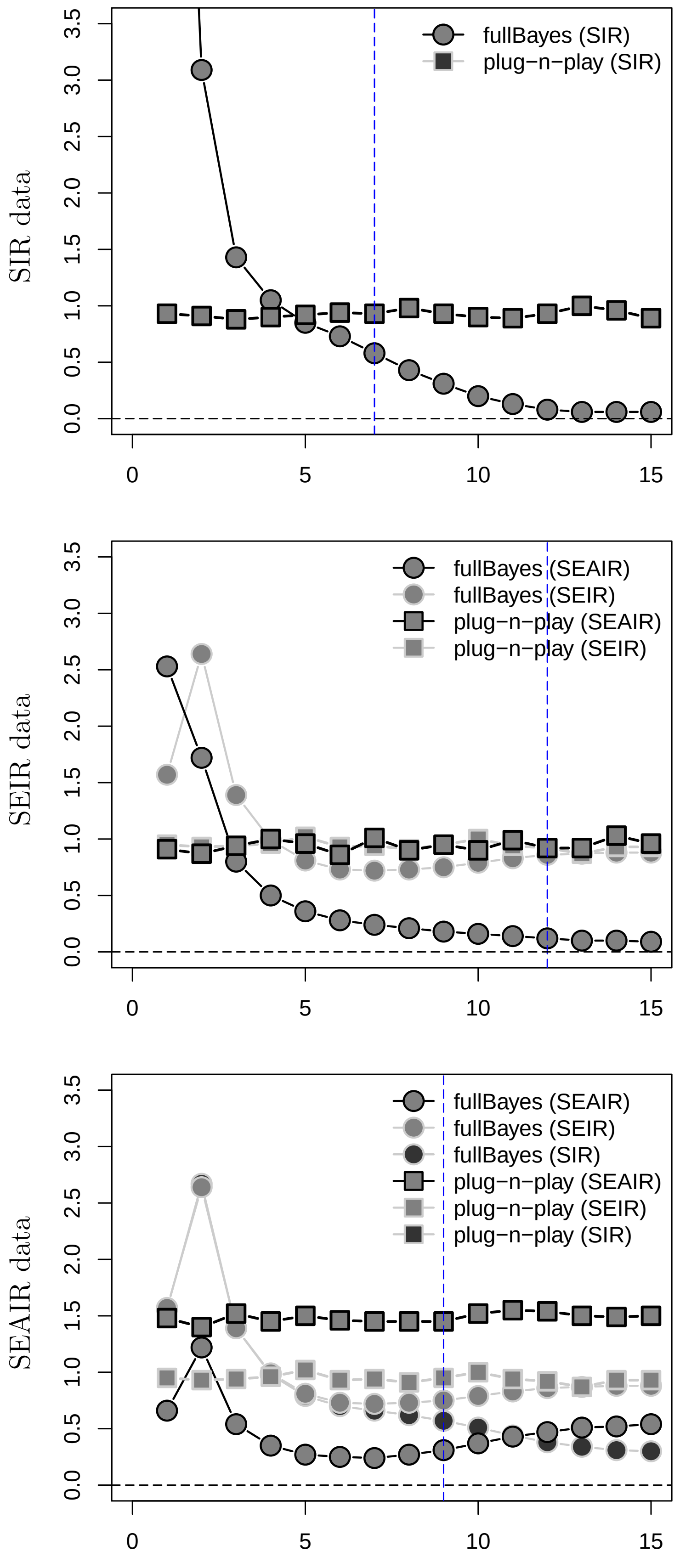

Figure 4: Influenza example 1 estimated MSE of fullBayes and plug-n-play R_0 estimators assuming unknown SI with SIR (top), SEIR (middle) and SEAIR (bottom) data (week on x-axis). The inflection points related to the epidemic data curve of the SIR, SEIR and SEAIR models are indicated by the blue dashed vertical lines.

2.2 Boxplots - Influenza Example 1

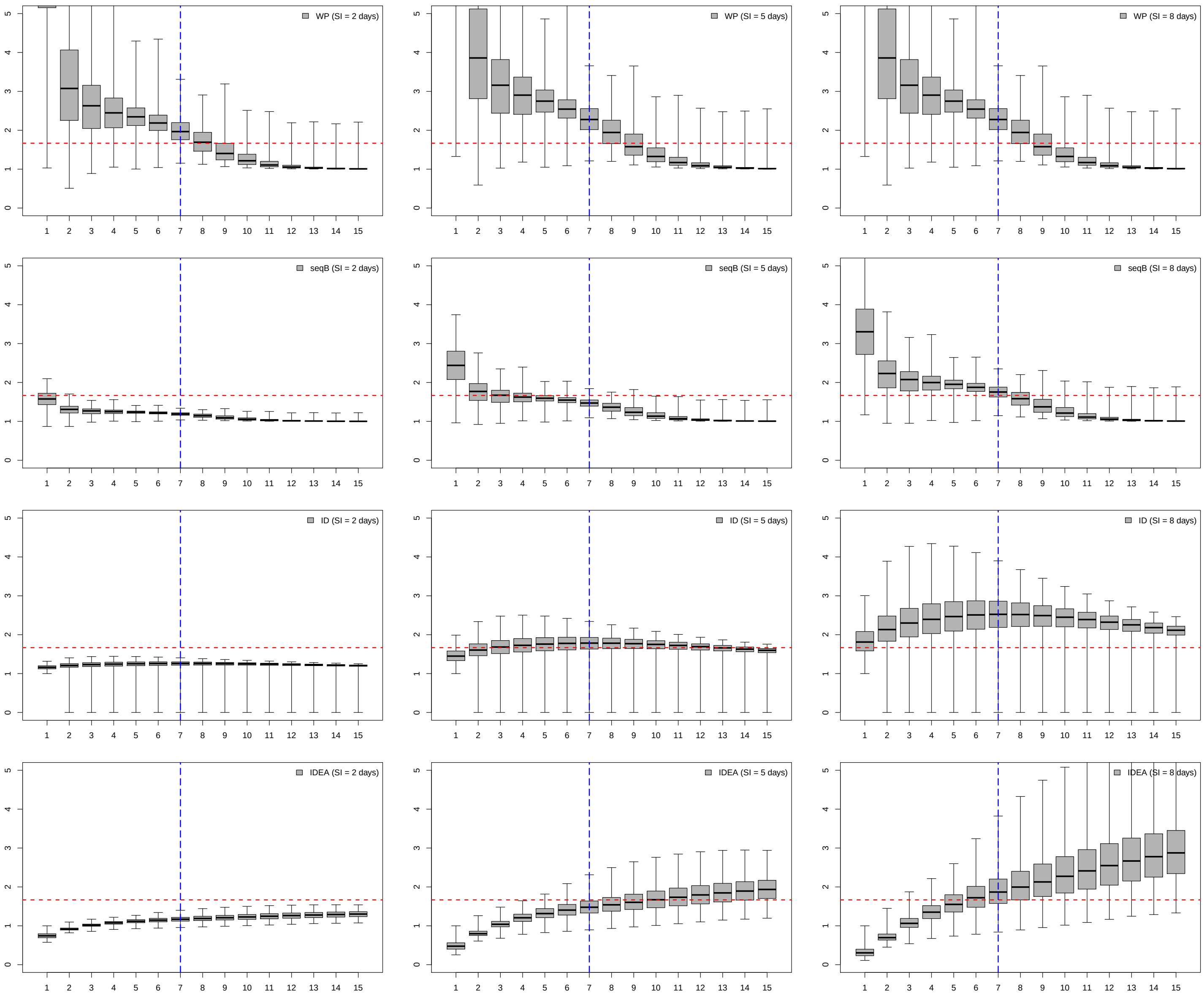

Figure 5: Influenza 1 R_0 estimates assuming known SI with SIR data (week on x-axis) for the WP (top row), seqB (second), ID (third), and IDEA (bottom) methods for known SI of 2 (left column), 5 (middle, true value for SIR model), and 8 (right, true value of SEIR and SEAIR mdoels) days. The true R_0 is given by the red dashed horizontal line. The inflection point related to the epidemic data curve of the SIR model is indicated by the blue dashed vertical lines.

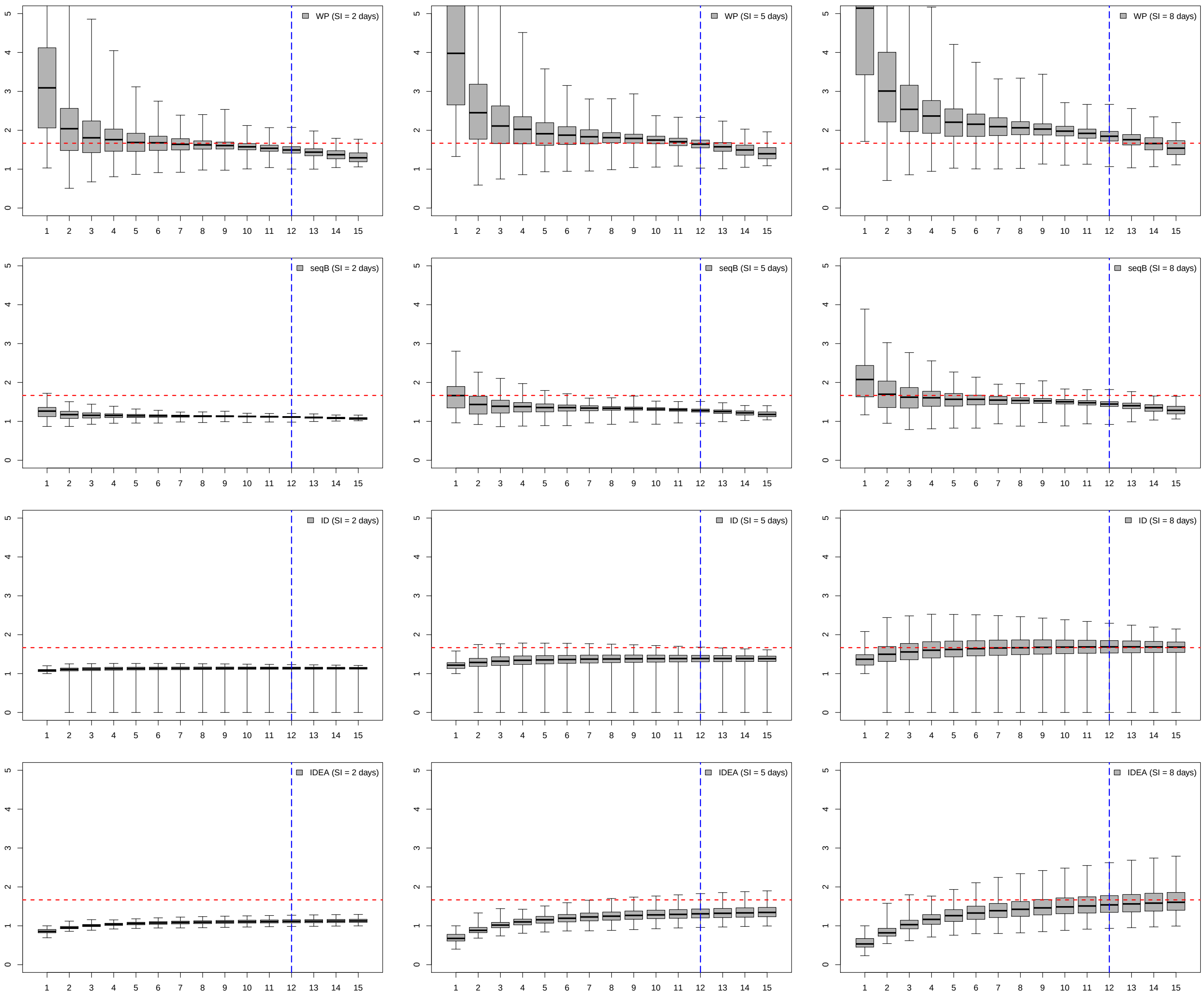

Figure 6: Influenza 1 R_0 estimates assuming known SI with SEIR data (week on x-axis) for the WP (top row), seqB (second), ID (third), and IDEA (bottom) methods for known SI of 2 (left column), 5 (middle, true value for SIR model), and 8 (right, true value of SEIR and SEAIR models) days. The true R_0 is given by the red dashed horizontal line. The inflection point related to the epidemic data curve of the SEIR model is indicated by the blue dashed vertical lines.

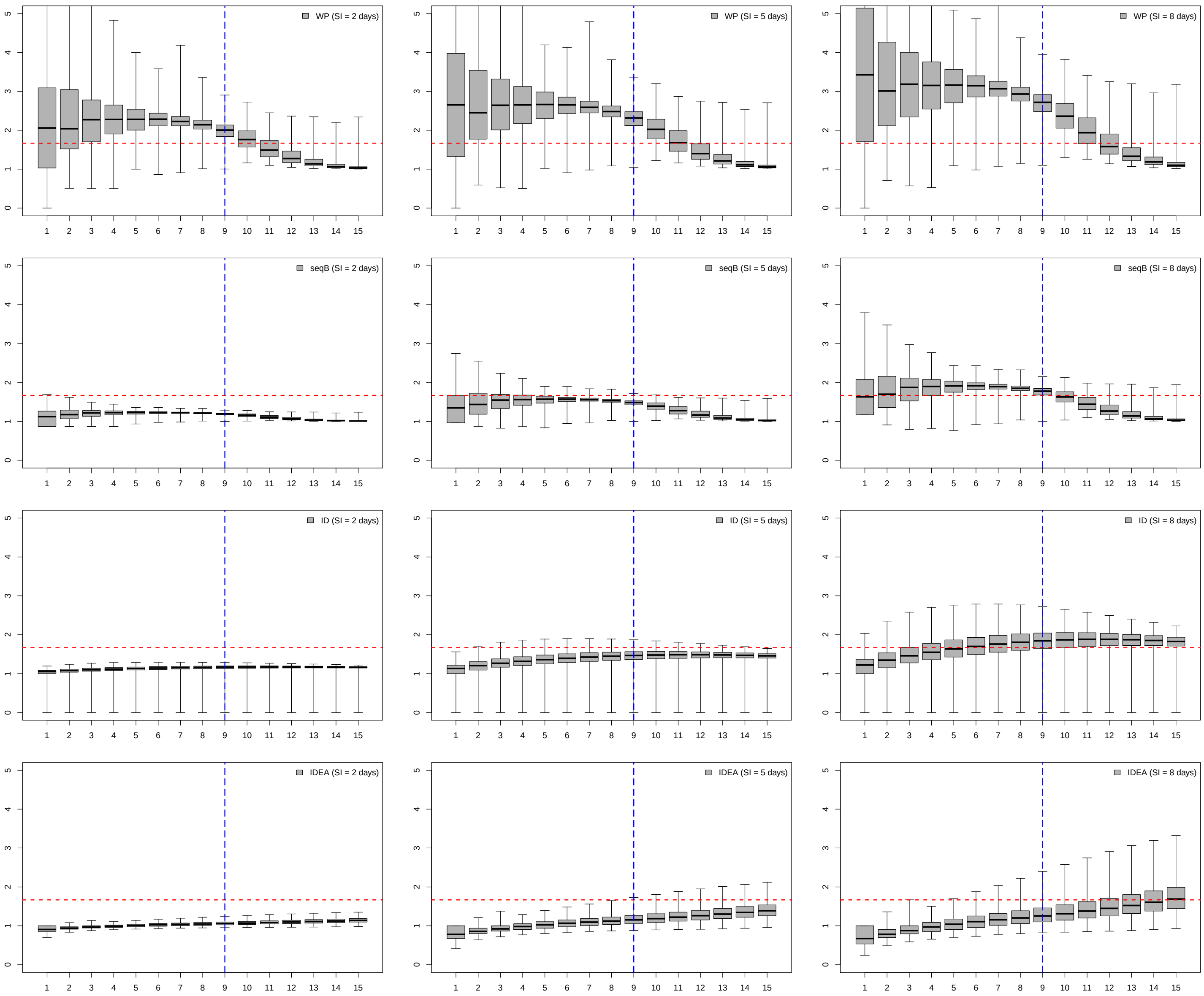

Figure 7: Influenza 1 R_0 estimates assuming known SI with SEAIR data (week on x-axis) for the WP (top row), seqB (second), ID (third), and IDEA (bottom) methods for known SI of 2 (left column), 5 (middle, true value for SIR model), and 8 (right, true value of SEIR and SEAIR mdoels) days. The true R_0 is given by the red dashed horizontal line. The inflection point related to the epidemic data curve of the SEAIR model is indicated by the blue dashed vertical lines.

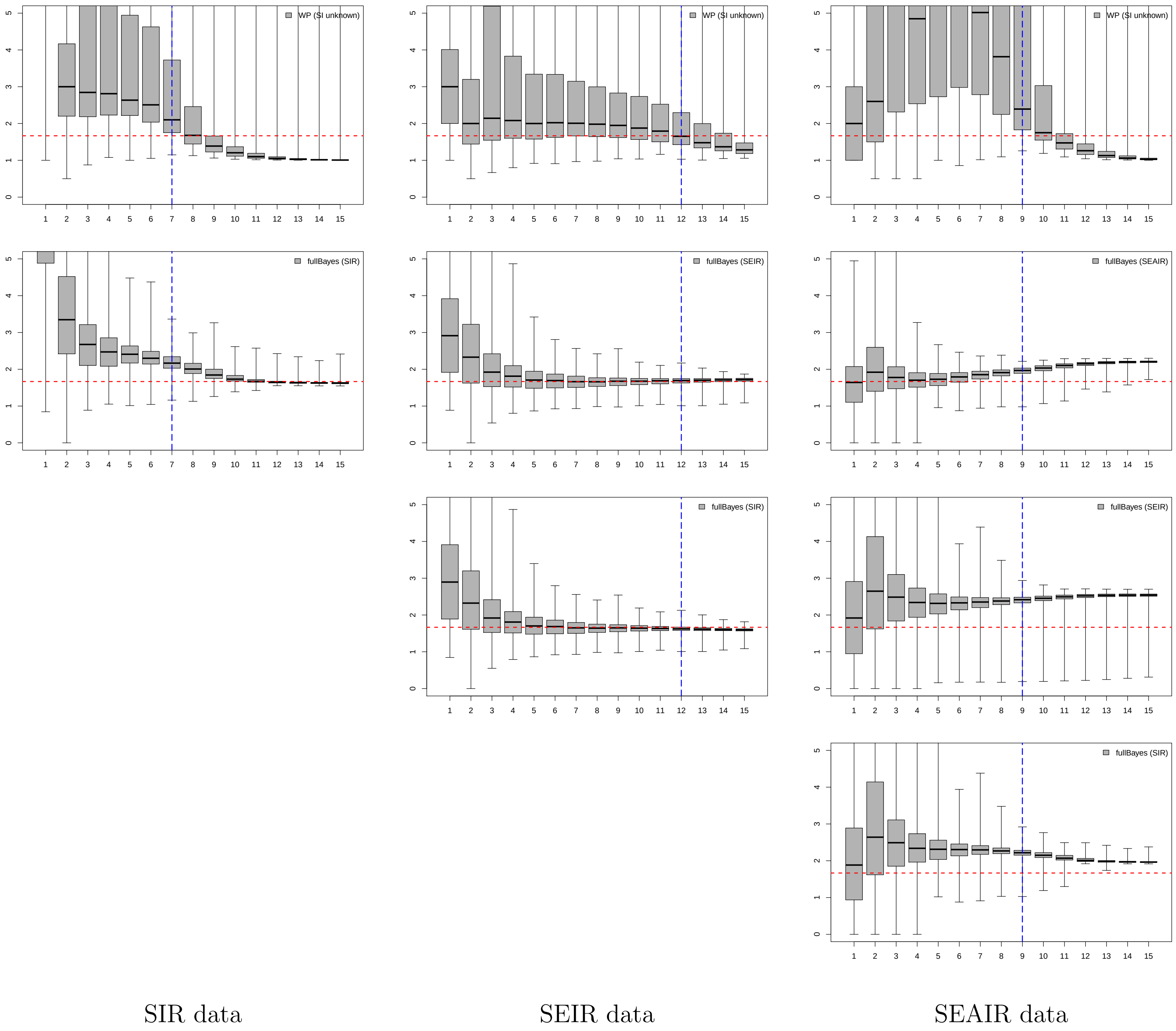

Figure 8: Influenza 1 R_0 estimates assuming unknown SI for the WP and fullBayes methods (week on x-axis). The WP method is applied to SIR, SEIR and SEAIR data. The fullBayes method assumes SIR, SEIR and SEAIR structures (rows), and is applied to SIR, SEIR, and SEAIR data (columns) with increasing dimension. The true R_0 is given by the red dashed horizontal line. The inflection points related to the epidemic data curve of the SIR, SEIR, or SEAIR models are indicated by the blue dashed vertical lines.

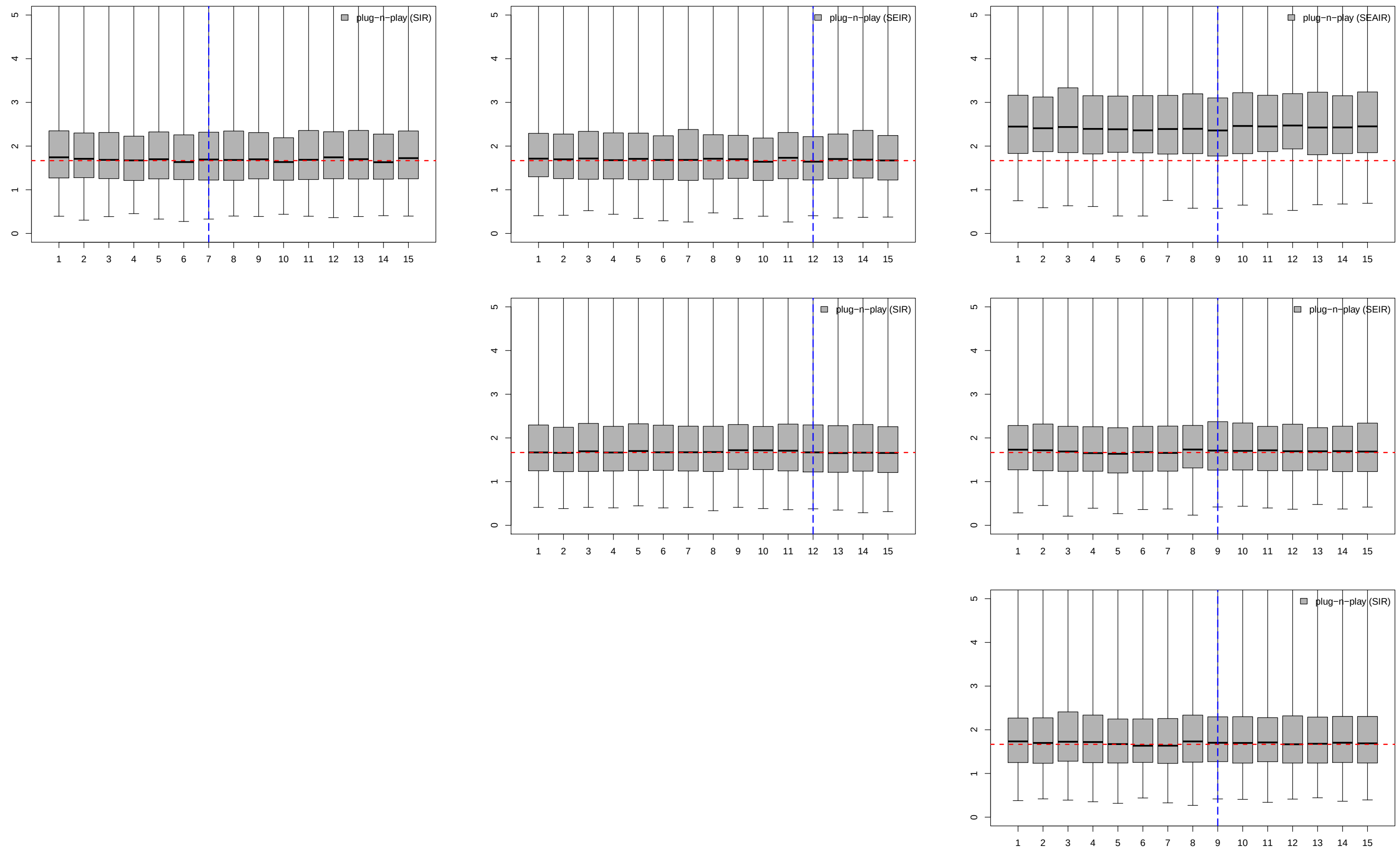

SIR data SEIR data SEAIR data

Figure 9: Influenza 1 R_0 estimates assuming unknown SI for the plug-n-play method (week on x-axis), assuming SIR, SEIR and SEAIR model structures (rows), applied to SIR, SEIR, and SEAIR data (columns) with increasing dimension. The true R_0 is given by the red dashed horizontal line. The inflection points related to the epidemic data curve of the SIR, SEIR, or SEAIR models are indicated by the blue dashed vertical lines.

2.3 Tables - Influenza Example 1

Table 4: Influenza example 1: WP, seq B, ID and IDEA methods applied to SIR data with known SI of 2, 5 (true value), 8 days. The true R_0 value is 5/3. Each row reports the median value and standard deviation (in brackets). NA count (if applicable) is the number of simulations that yield NA as a R_0 value. * (if applicable) denotes a standard deviation greater than one million.

		Week														
	SI	**1**	**2**	**3**	**4**	**5**	**6**	7	8	9	10	11	12	13	14	15
WP	2 days	8.2 (6.4)	3.1 (1.6)	2.6 (0.9)	2.4 (0.6)	2.3 (0.4)	2.2 (0.3)	2 (0.3)	1.7 (0.3)	1.4 (0.3)	1.2 (0.3)	1.1 (0.2)	1.1 (0.1)	1.0 (0.1)	1.0 (0.1)	1.0 (0.0)
	5 days	10.6 (8.3)	3.9 (2)	3.2 (1.1)	2.9 (0.8)	2.7 (0.5)	2.5 (0.4)	2.3 (0.4)	1.9 (0.4)	1.6 (0.4)	1.3 (0.3)	1.2 (0.3)	1.1 (0.2)	1.0 (0.1)	1.0 (0.1)	1.0 (0.1)
	8 days	10.6 (8.3)	3.9 (2)	3.2 (1.1)	2.9 (0.8)	2.7 (0.5)	2.5 (0.4)	2.3 (0.4)	1.9 (0.4)	1.6 (0.4)	1.3 (0.3)	1.2 (0.3)	1.1 (0.2)	1.0 (0.1)	1.0 (0.1)	1.0 (0.1)
seqB	2 days	1.6 (0.2)	1.3 (0.1)	1.3 (0.1)	1.2 (0.1)	1.2 (0.1)	1.2 (0)	1.2 (0)	1.1 (0.1)	1.1 (0.1)	1.1 (0.0)	1.0 (0.0)	1.0 (0.0)	1.0 (0.0)	1.0 (0.0)	1.0 (0.0)
	5 days	2.4 (0.5)	1.8 (0.3)	1.7 (0.2)	1.6 (0.2)	1.6 (0.1)	1.5 (0.1)	1.5 (0.1)	1.4 (0.1)	1.2 (0.1)	1.1 (0.1)	1.1 (0.1)	1.0 (0.1)	1.0 (0.1)	1.0 (0.0)	1.0 (0.0)
	8 days	3.3 (0.8)	2.2 (0.5)	2.1 (0.4)	2 (0.3)	2 (0.2)	1.9 (0.2)	1.8 (0.2)	1.6 (0.2)	1.4 (0.2)	1.2 (0.2)	1.1 (0.2)	1.1 (0.1)	1.0 (0.1)	1.0 (0.1)	1.0 (0.0)
ID	2 days	1.2 (0.1)	1.2 (0.1)	1.2 (0.1)	1.2 (0.1)	1.3 (0.1)	1.3 (0.1)	1.3 (0.1)	1.3 (0.1)	1.3 (0.1)	1.3 (0.1)	1.2 (0.1)	1.2 (0.1)	1.2 (0.1)	1.2 (0.1)	1.2 (0.1)
	5 days	1.4 (0.2)	1.6 (0.2)	1.7 (0.2)	1.7 (0.2)	1.8 (0.2)	1.8 (0.2)	1.8 (0.2)	1.8 (0.2)	1.8 (0.2)	1.8 (0.2)	1.7 (0.1)	1.7 (0.1)	1.7 (0.1)	1.6 (0.1)	1.6 (0.1)
	8 days	1.8 (0.4)	2.1 (0.5)	2.3 (0.5)	2.4 (0.5)	2.5 (0.5)	2.5 (0.5)	2.5 (0.5)	2.5 (0.4)	2.5 (0.4)	2.5 (0.3)	2.4 (0.3)	2.3 (0.3)	2.3 (0.2)	2.2 (0.2)	2.1 (0.2)
IDEA	NA count	0	2	3	3	3	3	3	3	3	3	3	3	3	3	3
	2 days	0.7 (0.1)	0.9 (0.0)	1 (0.0)	1.1 (0.1)	1.1 (0.1)	1.1 (0.1)	1.2 (0.1)	1.2 (0.1)	1.2 (0.1)	1.2 (0.1)	1.2 (0.1)	1.3 (0.1)	1.3 (0.1)	1.3 (0.1)	1.3 (0.1)
	5 days	0.5 (0.1)	0.8 (0.1)	1 (0.1)	1.2 (0.1)	1.3 (0.2)	1.4 (0.2)	1.5 (0.2)	1.5 (0.3)	1.6 (0.3)	1.7 (0.3)	1.7 (0.3)	1.8 (0.3)	1.8 (0.3)	1.9 (0.3)	1.9 (0.3)
	8 days	0.3 (0.2)	0.7 (0.1)	1.1 (0.2)	1.4 (0.2)	1.6 (0.3)	1.7 (0.4)	1.9 (0.5)	2 (0.5)	2.1 (0.6)	2.3 (0.7)	2.4 (0.7)	2.5 (0.7)	2.7 (0.8)	2.8 (0.8)	2.9 (0.8)

Table 5: Influenza example 1: WP, seq B, ID and IDEA methods applied to SEIR data with known SI of 2, 5, 8 (true value) days. The true R_0 value is 7/3. Each row reports the median value and standard deviation (in brackets). NA count (if applicable) is the number of simulations that yield NA as a R_0 value. * (if applicable) denotes a standard deviation greater than one million.

		Week														
	SI	**1**	**2**	**3**	**4**	**5**	**6**	7	8	9	10	11	12	13	14	15
WP	2 days	3.1 (2.0)	2 (0.9)	1.8 (0.6)	1.8 (0.4)	1.7 (0.3)	1.7 (0.3)	1.6 (0.2)	1.6 (0.2)	1.6 (0.2)	1.6 (0.1)	1.5 (0.1)	1.5 (0.1)	1.4 (0.1)	1.4 (0.1)	1.3 (0.1)
	5 days	4.0 (2.5)	2.5 (1.1)	2.1 (0.7)	2 (0.5)	1.9 (0.4)	1.9 (0.3)	1.8 (0.3)	1.8 (0.2)	1.8 (0.2)	1.7 (0.2)	1.7 (0.2)	1.6 (0.2)	1.6 (0.2)	1.5 (0.2)	1.4 (0.2)
	8 days	5.1 (3.3)	3 (1.4)	2.5 (0.9)	2.4 (0.6)	2.2 (0.5)	2.2 (0.4)	2.1 (0.4)	2.1 (0.3)	2 (0.3)	2 (0.2)	1.9 (0.2)	1.8 (0.2)	1.8 (0.2)	1.7 (0.2)	1.5 (0.2)
seqB	2 days	1.3 (0.2)	1.2 (0.1)	1.2 (0.1)	1.2 (0.1)	1.1 (0.1)	1.1 (0.0)	1.1 (0.0)	1.1 (0.0)	1.1 (0.0)	1.1 (0.0)	1.1 (0.0)	1.1 (0.0)	1.1 (0.0)	1.1 (0.0)	1.1 (0.0)
	5 days	1.7 (0.4)	1.4 (0.3)	1.4 (0.2)	1.4 (0.2)	1.4 (0.1)	1.4 (0.1)	1.3 (0.1)	1.3 (0.1)	1.3 (0.1)	1.3 (0.1)	1.3 (0.1)	1.3 (0.1)	1.3 (0.1)	1.2 (0.1)	1.2 (0.1)
	8 days	2.1 (0.6)	1.7 (0.5)	1.6 (0.4)	1.6 (0.3)	1.6 (0.2)	1.6 (0.2)	1.5 (0.2)	1.5 (0.1)	1.5 (0.1)	1.5 (0.1)	1.5 (0.1)	1.4 (0.1)	1.4 (0.1)	1.3 (0.1)	1.3 (0.1)
ID	2 days	1.1 (0.0)	1.2 (0.1)	1.2 (0.1)	1.2 (0.1)	1.2 (0.1)	1.2 (0.1)	1.2 (0.1)	1.2 (0.1)	1.2 (0.0)	1.2 (0.0)	1.2 (0.0)	1.2 (0.0)	1.2 (0.0)	1.2 (0.0)	1.2 (0.0)
	5 days	1.4 (0.1)	1.5 (0.2)	1.6 (0.2)	1.6 (0.2)	1.6 (0.2)	1.6 (0.2)	1.7 (0.2)	1.7 (0.2)	1.7 (0.2)	1.7 (0.1)	1.6 (0.1)	1.6 (0.1)	1.6 (0.1)	1.6 (0.1)	1.6 (0.1)
	8 days	1.7 (0.3)	1.9 (0.4)	2.0 (0.4)	2.1 (0.4)	2.2 (0.4)	2.2 (0.4)	2.3 (0.4)	2.3 (0.4)	2.3 (0.3)	2.3 (0.3)	2.2 (0.3)	2.2 (0.2)	2.1 (0.2)	2.1 (0.2)	2.0 (0.2)
	NA count	0	2	3	3	3	3	3	3	3	3	3	3	3	3	3
IDEA	2 days	0.9 (0.1)	1 (0.0)	1 (0.0)	1 (0.0)	1.1 (0.0)	1.1 (0.0)	1.1 (0.1)	1.1 (0.1)	1.1 (0.1)	1.1 (0.1)	1.1 (0.1)	1.1 (0.1)	1.1 (0.1)	1.1 (0.1)	1.1 (0.1)
	5 days	0.7 (0.1)	0.9 (0.1)	1 (0.1)	1.1 (0.1)	1.2 (0.1)	1.2 (0.1)	1.2 (0.1)	1.2 (0.1)	1.3 (0.2)	1.3 (0.2)	1.3 (0.2)	1.3 (0.2)	1.3 (0.2)	1.3 (0.2)	1.3 (0.2)
	8 days	0.5 (0.2)	0.8 (0.2)	1 (0.2)	1.2 (0.2)	1.3 (0.2)	1.3 (0.2)	1.4 (0.3)	1.4 (0.3)	1.5 (0.3)	1.5 (0.3)	1.5 (0.3)	1.5 (0.3)	1.6 (0.3)	1.6 (0.3)	1.6 (0.3)

Table 6: Influenza example 1: WP, seq B, ID and IDEA methods applied to SEAIR data with known SI of 2, 5, 8 (true value) days. The true R_0 value is 7/3. Each row reports the median value and standard deviation (in brackets). NA count (if applicable) is the number of simulations that yield NA as a R_0 value. * (if applicable) denotes a standard deviation greater than one million.

		Week														
	SI	**1**	**2**	**3**	**4**	**5**	**6**	7	8	9	10	11	12	13	14	15
WP	2 days	2.1	2.0	2.3	2.3	2.3	2.3	2.2	2.1	2	1.8	1.5	1.3	1.1	1.1	1.0
		(1.5)	(1.3)	(0.8)	(0.6)	(0.5)	(0.3)	(0.3)	(0.2)	(0.2)	(0.3)	(0.3)	(0.3)	(0.2)	(0.2)	(0.1)
	5 days	2.7	2.5	2.6	2.7	2.7	2.7	2.6	2.5	2.3	2	1.7	1.4	1.2	1.1	1.1
		(1.9)	(1.6)	(1)	(0.8)	(0.6)	(0.4)	(0.3)	(0.3)	(0.3)	(0.3)	(0.4)	(0.3)	(0.3)	(0.2)	(0.2)
	8 days	3.4	3	3.2	3.2	3.2	3.1	3.1	2.9	2.7	2.4	1.9	1.6	1.3	1.2	1.1
		(2.5)	(1.9)	(1.2)	(0.9)	(0.7)	(0.5)	(0.4)	(0.3)	(0.3)	(0.4)	(0.5)	(0.4)	(0.4)	(0.3)	(0.2)
seqB	NA count	12	0	0	0	0	0	0	0	0	0	0	0	0	0	0
	2 days	1.1	1.2	1.2	1.2	1.2	1.2	1.2	1.2	1.2	1.2	1.1	1.1	1.0	1.0	1.0
		(0.2)	(0.2)	(0.1)	(0.1)	(0.1)	(0)	(0)	(0)	(0)	(0)	(0.1)	(0.1)	(0.0)	(0.0)	(0.0)
	5 days	1.3	1.4	1.5	1.6	1.6	1.6	1.6	1.5	1.5	1.4	1.3	1.2	1.1	1.0	1.0
		(0.4)	(0.4)	(0.3)	(0.2)	(0.2)	(0.1)	(0.1)	(0.1)	(0.1)	(0.1)	(0.1)	(0.1)	(0.1)	(0.1)	(0.1)
	8 days	1.6	1.7	1.9	1.9	1.9	1.9	1.9	1.8	1.8	1.6	1.4	1.3	1.1	1.1	1
		(0.5)	(0.5)	(0.4)	(0.3)	(0.3)	(0.2)	(0.1)	(0.1)	(0.1)	(0.2)	(0.2)	(0.2)	(0.2)	(0.1)	(0.1)
ID	2 days	1.1	1.1	1.2	1.2	1.2	1.2	1.2	1.2	1.2	1.2	1.2	1.2	1.2	1.2	1.2
		(0.0)	(0.1)	(0.1)	(0.1)	(0.1)	(0.1)	(0.1)	(0.0)	(0.0)	(0.0)	(0.0)	(0.0)	(0.0)	(0.0)	(0.0)
	5 days	1.3	1.4	1.4	1.5	1.5	1.5	1.5	1.5	1.6	1.6	1.6	1.5	1.5	1.5	1.5
		(0.1)	(0.2)	(0.2)	(0.2)	(0.2)	(0.2)	(0.2)	(0.2)	(0.1)	(0.1)	(0.1)	(0.1)	(0.1)	(0.1)	(0.1)
	8 days	1.5	1.7	1.8	1.9	1.9	2.0	2.0	2.0	2.0	2.0	2.0	2.0	2.0	2.0	1.9
		(0.2)	(0.3)	(0.3)	(0.4)	(0.4)	(0.3)	(0.3)	(0.3)	(0.3)	(0.3)	(0.3)	(0.2)	(0.2)	(0.2)	(0.2)
IDEA	NA count	12	53	69	74	74	76	76	76	76	76	76	76	76	76	76
	2 days	0.9	0.9	1	1	1	1	1	1	1.1	1.1	1.1	1.1	1.1	1.1	1.1
		(0.1)	(0.0)	(0.0)	(0.0)	(0.0)	(0.0)	(0.0)	(0.1)	(0.1)	(0.1)	(0.1)	(0.1)	(0.1)	(0.1)	(0.1)
	5 days	0.8	0.9	0.9	1.0	1.0	1.1	1.1	1.1	1.2	1.2	1.2	1.3	1.3	1.3	1.4
		(0.2)	(0.1)	(0.1)	(0.1)	(0.1)	(0.1)	(0.1)	(0.1)	(0.1)	(0.2)	(0.2)	(0.2)	(0.2)	(0.2)	(0.2)
	8 days	0.7	0.8	0.9	1.0	1.0	1.1	1.2	1.2	1.3	1.3	1.4	1.4	1.5	1.6	1.7
		(0.2)	(0.2)	(0.1)	(0.2)	(0.2)	(0.2)	(0.2)	(0.2)	(0.3)	(0.3)	(0.3)	(0.3)	(0.4)	(0.4)	(0.4)

Table 7: Influenza example 1: WP, fullBayes, and plug-n-play methods applied to SIR, SEIR, and SEAIR data, with unknown SI. The true R_0 value is 5/3 for the SIR model, and 7/3 for the SEIR and SEAIR models. fullBayes and plug-n-play methods assume SIR, SEIR, or SEAIR model structures. Each row reports the median value and standard deviation (in brackets). NA count (if applicable) is the number of simulations that yield NA as a R_0 value. * (if applicable) denotes a standard deviation greater than one million.

		Week														
	data	1	2	3	4	5	6	7	8	9	10	11	12	13	14	15
WP	SIR	8.5 (639.5)	3 (28294.5)	2.8 (1503.2)	2.8 (2463.7)	2.6 (4223.2)	2.5 (5352.7)	2.1 (53949.8)	1.7 (1883.7)	1.4 (1129.7)	1.2 (295.7)	1.1 (7990.3)	1.1 (1908)	1.0 (0.2)	1.0 (16.2)	1.0 (1855.2)
	SEIR	3.0 (2.7)	2.0 (292.9)	2.1 (525)	2.1 (164.9)	2.0 (371)	2.0 (488.7)	2.0 (191.5)	2.0 (138.6)	1.9 (8.9)	1.9 (5.0)	1.8 (5.7)	1.7 (28.4)	1.5 (38.6)	1.4 (2.4)	1.3 (3.5)
	NA count	12	0	0	0	0	0	0	0	0	0	0	0	0	0	0
WP	SEAIR	2 (2.0)	2.6 (*)	5.6 (2940.1)	4.8 (23618.6)	5.3 (10979.6)	5.8 (31111.3)	5 (32063.5)	3.8 (24478)	2.4 (156130.2)	1.8 (493020.7)	1.5 (763945.7)	1.3 (143997.8)	1.1 (*)	1.1 (473645.2)	1 (60289.9)
plug-n-play (SIR)	SIR	1.7 (0.9)	1.7 (0.9)	1.7 (0.9)	1.7 (0.9)	1.7 (0.9)	1.6 (0.9)	1.7 (0.9)	1.7 (0.9)	1.7 (0.9)	1.6 (0.9)	1.7 (0.9)	1.7 (0.9)	1.7 (1)	1.6 (0.9)	1.7 (0.9)
	SEIR	1.7 (0.9)	1.7 (0.9)	1.7 (0.9)	1.7 (0.9)	1.7 (0.9)	1.7 (0.9)	1.7 (0.9)	1.7 (1)	1.7 (0.9)	1.7 (0.9)	1.7 (0.9)	1.7 (0.9)	1.7 (0.9)	1.7 (0.9)	1.7 (0.9)
	SEAIR	1.7 (0.9)	1.7 (1)	1.7 (1)	1.7 (0.9)	1.7 (0.9)	1.6 (0.9)	1.6 (0.9)	1.7 (0.9)	1.7 (0.9)	1.7 (0.9)	1.7 (0.8)	1.7 (1)	1.7 (0.9)	1.7 (0.9)	1.7 (0.9)
plug-n-play (SEIR)	SEIR	1.7 (0.9)	1.7 (0.8)	1.7 (0.9)	1.7 (1.0)	1.7 (0.9)	1.7 (0.8)	1.7 (1)	1.7 (0.9)	1.7 (0.9)	1.6 (0.9)	1.7 (1.0)	1.6 (0.9)	1.7 (0.9)	1.7 (1.0)	1.7 (0.9)
	SEAIR	2.7 (0.5)	2.7 (0.4)	2.7 (0.5)	2.7 (0.4)	2.7 (0.5)	2.7 (0.5)	2.7 (0.5)	2.7 (0.4)	2.7 (0.5)	2.7 (0.4)	2.7 (0.5)	2.7 (0.5)	2.7 (0.5)	2.7 (0.5)	2.7 (0.5)
plug-n-play (SEAIR)	SEAIR	1.7 (0.9)	1.7 (0.9)	1.7 (0.9)	1.7 (0.9)	1.6 (1)	1.7 (0.9)	1.7 (0.9)	1.7 (0.9)	1.7 (0.9)	1.7 (1)	1.7 (0.9)	1.7 (0.9)	1.7 (0.9)	1.7 (0.9)	1.7 (0.9)
fullBayes (SIR)	SIR	7.9 (6.3)	3.3 (2.2)	2.7 (0.9)	2.5 (0.6)	2.4 (0.4)	2.3 (0.3)	2.2 (0.3)	2 (0.2)	1.8 (0.2)	1.7 (0.2)	1.7 (0.1)	1.6 (0.1)	1.6 (0.1)	1.6 (0.0)	1.6 (0.0)
	SEIR	2.9 (1.9)	2.3 (1.4)	1.9 (0.7)	1.8 (0.5)	1.7 (0.4)	1.7 (0.3)	1.7 (0.2)	1.6 (0.2)	1.6 (0.2)	1.6 (0.1)	1.6 (0.1)	1.6 (0.1)	1.6 (0.1)	1.6 (0.1)	1.6 (0.1)
	SEAIR	1.9 (1.5)	2.6 (2.2)	2.5 (1.0)	2.3 (0.7)	2.3 (0.5)	2.3 (0.3)	2.3 (0.3)	2.3 (0.2)	2.2 (0.1)	2.1 (0.1)	2.1 (0.1)	2.0 (0.1)	2.0 (0.1)	2.0 (0.0)	2.0 (0.0)
fullBayes (SEIR)	SEIR	2.9 (1.9)	2.3 (1.4)	1.9 (0.7)	1.8 (0.5)	1.7 (0.4)	1.7 (0.3)	1.7 (0.2)	1.7 (0.2)	1.7 (0.2)	1.7 (0.2)	1.7 (0.1)	1.7 (0.1)	1.7 (0.1)	1.7 (0.1)	1.7 (0.1)
	SEAIR	1.9 (1.5)	2.6 (2.2)	2.5 (1.1)	2.3 (0.7)	2.3 (0.5)	2.3 (0.4)	2.4 (0.3)	2.4 (0.3)	2.4 (0.3)	2.5 (0.3)	2.5 (0.2)	2.5 (0.2)	2.5 (0.2)	2.5 (0.2)	2.5 (0.2)
fullBayes (SEAIR)	SEAIR	1.6 (0.7)	1.9 (1.1)	1.8 (0.5)	1.7 (0.3)	1.7 (0.3)	1.8 (0.2)	1.9 (0.2)	1.9 (0.2)	2.0 (0.1)	2.0 (0.1)	2.1 (0.1)	2.2 (0.1)	2.2 (0.1)	2.2 (0.1)	2.2 (0.0)

3 Influenza 2 Example

Parameter values are listed in Table 2. Influenza 2 parameters are such that $R_0 = 5/3$ and the serial interval is 5 days for the SIR, SEIR, and SEAIR models.

For the WP method, we obtain values of p_t, representing the serial distribution. The serial distribution values, discretized to weeks, from the SIR/SEIR/SEAIR model epidemic data for influenza 1 are

- 0.75 0.19 0.05 0.01
- 0.61 0.29 0.08 0.02
- 0.58 0.31 0.08 0.02 0.01 .

Given a known serial interval μ, we obtain the following serial distributions, discretized to weeks:

- ($\mu = 5$) 0.75 0.19 0.05 0.01
- ($\mu = 2$) 0.97 0.03
- ($\mu = 8$) 0.58 0.24 0.10 0.04 0.02 0.01

When the serial distribution is unknown, the WP method determines serial distributions. fullBayes and plug-n-play also return estimates of the serial distribution, for the SIR, SEIR, and SEAIR examples. Estimates of the serial distribution are shown in Table 8. Note that for the WP method, we make the standard actuarial mid-week assumption. That is, we assume that within a week, an event occurs on average in the middle of the week.

Table 8: Influenza example 2 - estimating SI (value in weeks): WP, fullBayes, and plug-n-play methods applied ot SIR, SEIR, and SEAIR data, with SI unknown. fullBayes and plug-n-play methods assume SIR, SEIR, or SEAIR model structures. Each row reports the median value. The true SI (in weeks) is for 0.71 for all models.

	data	Week 1	2	3	4	5	6	7	8	9	10	11	12	13	14	15
WP (SI unknown)	SIR	0.5 (27.7)	0.5 (4.1)	0.5 (5.8)	0.7 (5)	0.8 (4.6)	0.9 (4.8)	0.5 (5)	0.5 (5.3)	0.5 (3.8)	0.5 (3.9)	0.5 (4.8)	0.5 (2.4)	0.5 (1.7)	0.5 (0.2)	0.5 (1.8)
	SEIR	0.5 (27.9)	0.5 (3.6)	0.5 (5.6)	0.5 (4.7)	0.7 (4.6)	0.7 (4.2)	0.6 (4.2)	0.5 (3.7)	0.5 (3.8)	0.5 (3.5)	0.5 (2.6)	0.5 (2.6)	0.5 (1.5)	0.5 (0.3)	0.5 (0.1)
	SEAIR	0.5 (25.2)	0.5 (5.3)	0.7 (5.9)	0.9 (5.3)	1 (5.2)	1.1 (5.4)	1.1 (5.2)	0.8 (4.4)	0.5 (4)	0.5 (3.5)	0.5 (2.6)	0.5 (2.8)	0.5 (1.4)	0.5 (1.8)	0.5 (0.3)
plug-n-play (SIR)	SIR	0.7 (0.3)	0.7 (0.3)	0.7 (0.3)	0.7 (0.3)	0.8 (0.3)	0.7 (0.3)	0.7 (0.3)	0.7 (0.3)	0.7 (0.3)	0.7 (0.3)	0.7 (0.3)	0.7 (0.3)	0.7 (0.3)	0.7 (0.3)	0.7 (0.3)
	SEIR	0.7 (0.3)	0.7 (0.3)	0.7 (0.3)	0.7 (0.3)	0.7 (0.3)	0.7 (0.3)	0.7 (0.3)	0.7 (0.3)	0.7 (0.3)	0.7 (0.3)	0.8 (0.3)	0.7 (0.3)	0.7 (0.3)	0.7 (0.3)	0.7 (0.3)
	SEAIR	0.7 (0.3)	0.7 (0.3)	0.7 (0.3)	0.7 (0.3)	0.7 (0.3)	0.7 (0.3)	0.7 (0.2)	0.7 (0.3)	0.7 (0.3)	0.7 (0.3)	0.7 (0.3)	0.8 (0.3)	0.7 (0.3)	0.7 (0.3)	0.7 (0.3)
plug-n-play (SEIR)	SEIR	0.8 (0.2)	0.8 (0.2)	0.8 (0.2)	0.8 (0.2)	0.8 (0.2)	0.8 (0.2)	0.8 (0.2)	0.8 (0.2)	0.8 (0.2)	0.8 (0.2)	0.8 (0.2)	0.8 (0.2)	0.8 (0.2)	0.8 (0.2)	0.8 (0.2)
	SEAIR	0.8 (0.2)	0.8 (0.2)	0.8 (0.2)	0.8 (0.2)	0.8 (0.2)	0.8 (0.2)	0.7 (0.2)	0.8 (0.2)	0.7 (0.2)	0.8 (0.2)	0.8 (0.2)	0.8 (0.2)	0.7 (0.2)	0.8 (0.2)	0.8 (0.2)
plug-n-play (SEAIR)	SEAIR	0.8 (0.2)	0.8 (0.2)	0.8 (0.2)	0.8 (0.2)	0.8 (0.2)	0.8 (0.2)	0.8 (0.2)	0.8 (0.2)	0.8 (0.2)	0.8 (0.2)	0.8 (0.2)	0.8 (0.2)	0.8 (0.2)	0.8 (0.2)	0.8 (0.2)
fullBayes (SIR)	SIR	3.1 (2)	2.2 (0.8)	2 (0.4)	1.8 (0.3)	1.7 (0.2)	1.6 (0.2)	1.5 (0.2)	1.3 (0.2)	1.2 (0.2)	1.1 (0.1)	1 (0.1)	1 (0.1)	1 (0.1)	1 (0)	1 (0)
	SEIR	2.3 (1.2)	2.2 (0.7)	1.9 (0.4)	1.8 (0.3)	1.7 (0.2)	1.6 (0.1)	1.5 (0.1)	1.4 (0.1)	1.3 (0.1)	1.2 (0.1)	1.1 (0.1)	1.1 (0.1)	1 (0.1)	1 (0.1)	1 (0)
	SEAIR	1.9 (0.7)	2.1 (0.7)	1.9 (0.4)	1.8 (0.3)	1.7 (0.2)	1.6 (0.2)	1.5 (0.1)	1.5 (0.1)	1.4 (0.1)	1.3 (0.1)	1.2 (0.1)	1.1 (0.1)	1.1 (0.1)	1 (0.1)	1 (0)
fullBayes (SEIR)	SEIR	3.7 (1.5)	2.8 (0.7)	2.9 (0.3)	3 (0.2)	3 (0.2)	2.9 (0.3)	2.9 (0.3)	3 (0.2)	3.1 (0.3)	3.3 (0.4)	3.8 (0.6)	4.4 (0.8)	5.2 (1)	6.1 (1.2)	7 (1.3)
	SEAIR	2.4 (0.7)	2.8 (0.6)	2.9 (0.3)	3 (0.2)	3 (0.2)	3 (0.2)	3 (0.2)	3 (0.2)	3.1 (0.2)	3.2 (0.3)	3.4 (0.4)	3.9 (0.6)	4.5 (0.8)	5.3 (1)	6.1 (1.1)
fullBayes (SEAIR)	SEAIR	1.2 (0.4)	1.1 (0.4)	1 (0.2)	1 (0.1)	1 (0.2)	1.2 (0.2)	1.3 (0.1)	1.4 (0.1)	1.4 (0.1)	1.4 (0.1)	1.3 (0)	1.3 (0)	1.3 (0)	1.2 (0)	1.2 (0)

3.1 MSE - Influenza Example 2

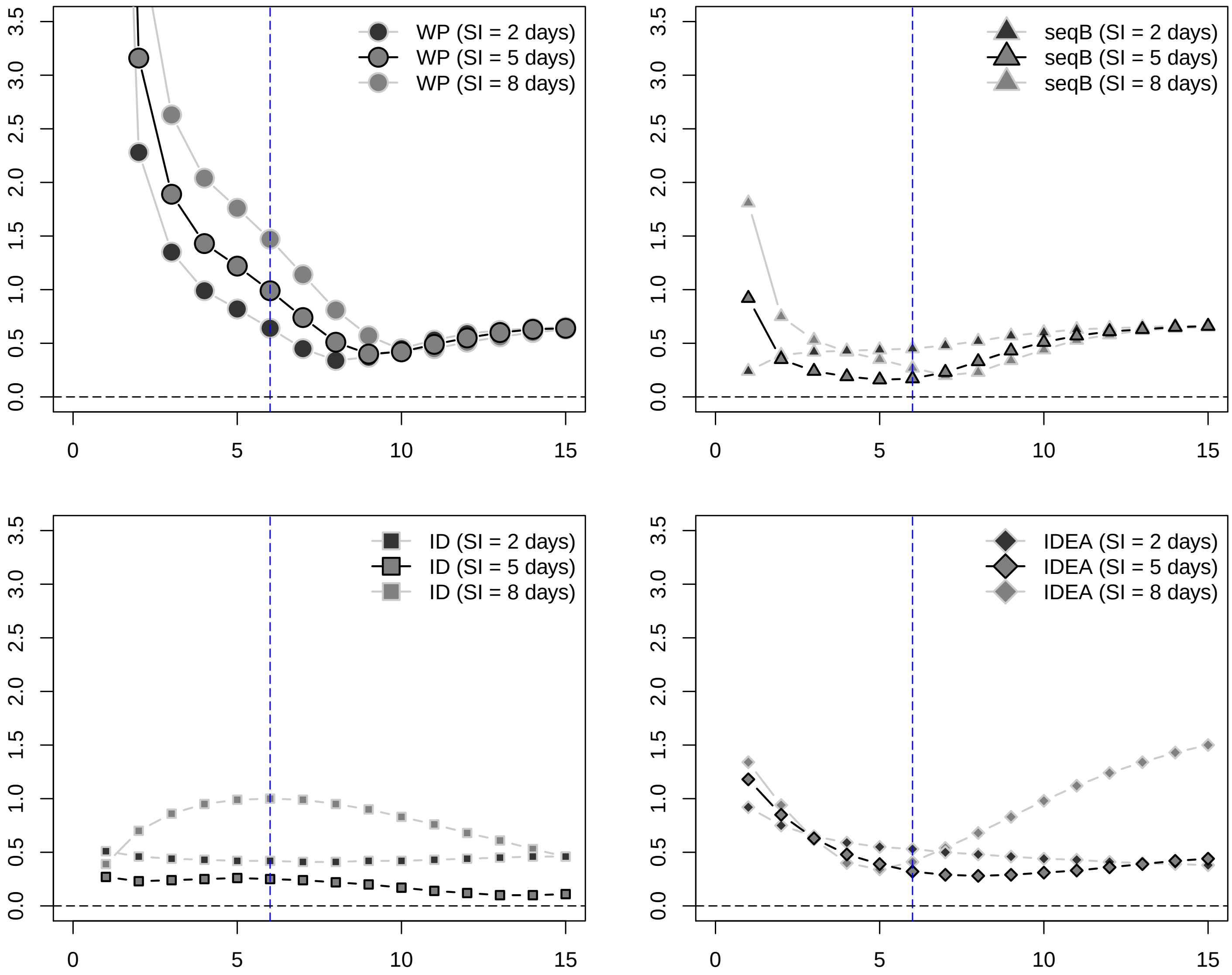

Figure 10: Influenza example 2 estimated MSE of WP, seqB, ID and IDEA R_0 estimators assuming known SI with SIR data (week on x-axis) under well-specified and misspecified cases of the serial interval, where the true serial interval is 5 days. The inflection point related to the epidemic data curve of the SIR model is indicated by the blue dashed vertical line.

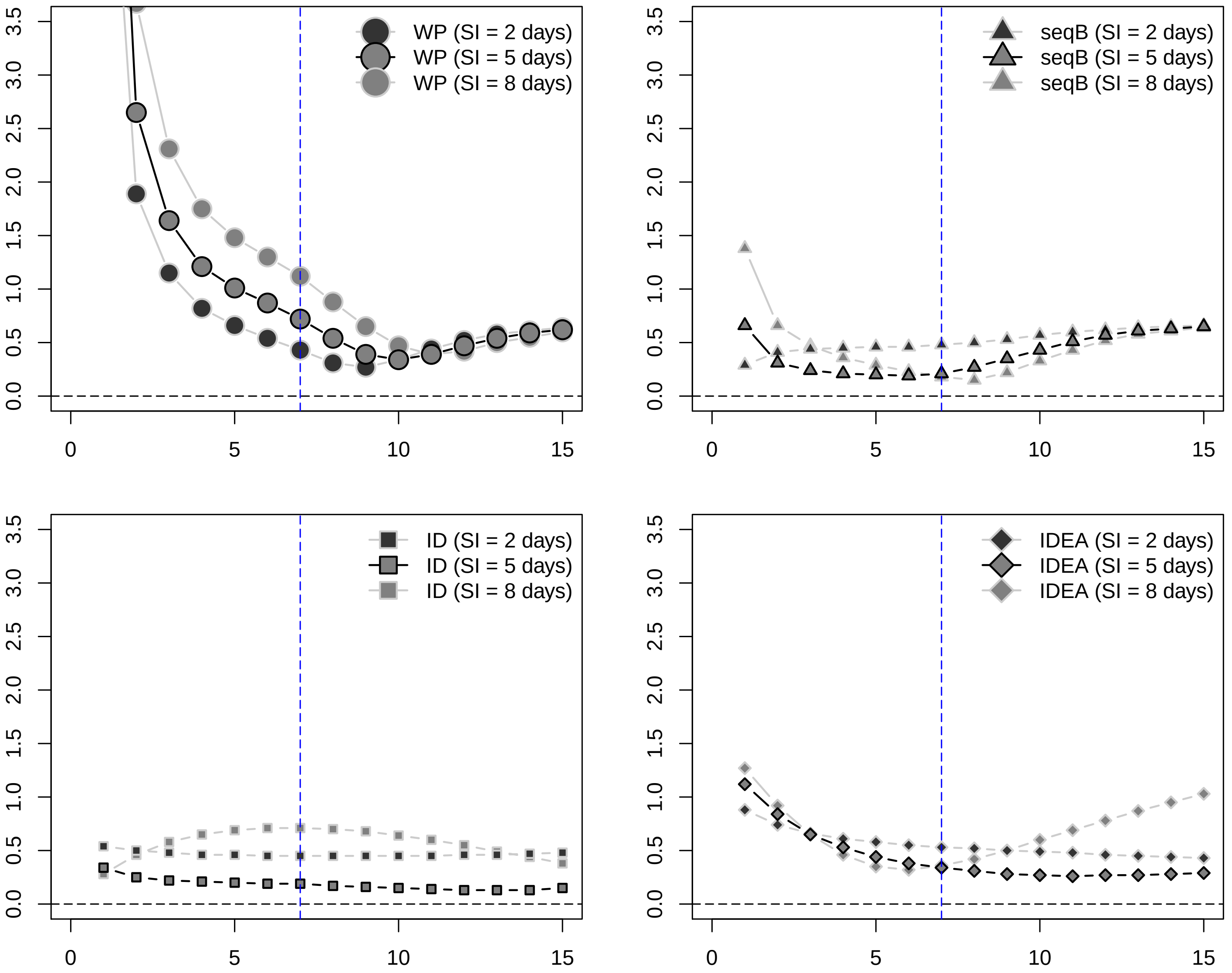

Figure 11: Influenza example 2 estimated MSE of WP, seqB, ID and IDEA R_0 estimators assuming known SI with SEIR data (week on x-axis) under well-specified and misspecified cases of the serial interval, where the true serial interval is 5 days. The inflection point related to the epidemic data curve of the SEIR model is indicated by the blue dashed vertical line.

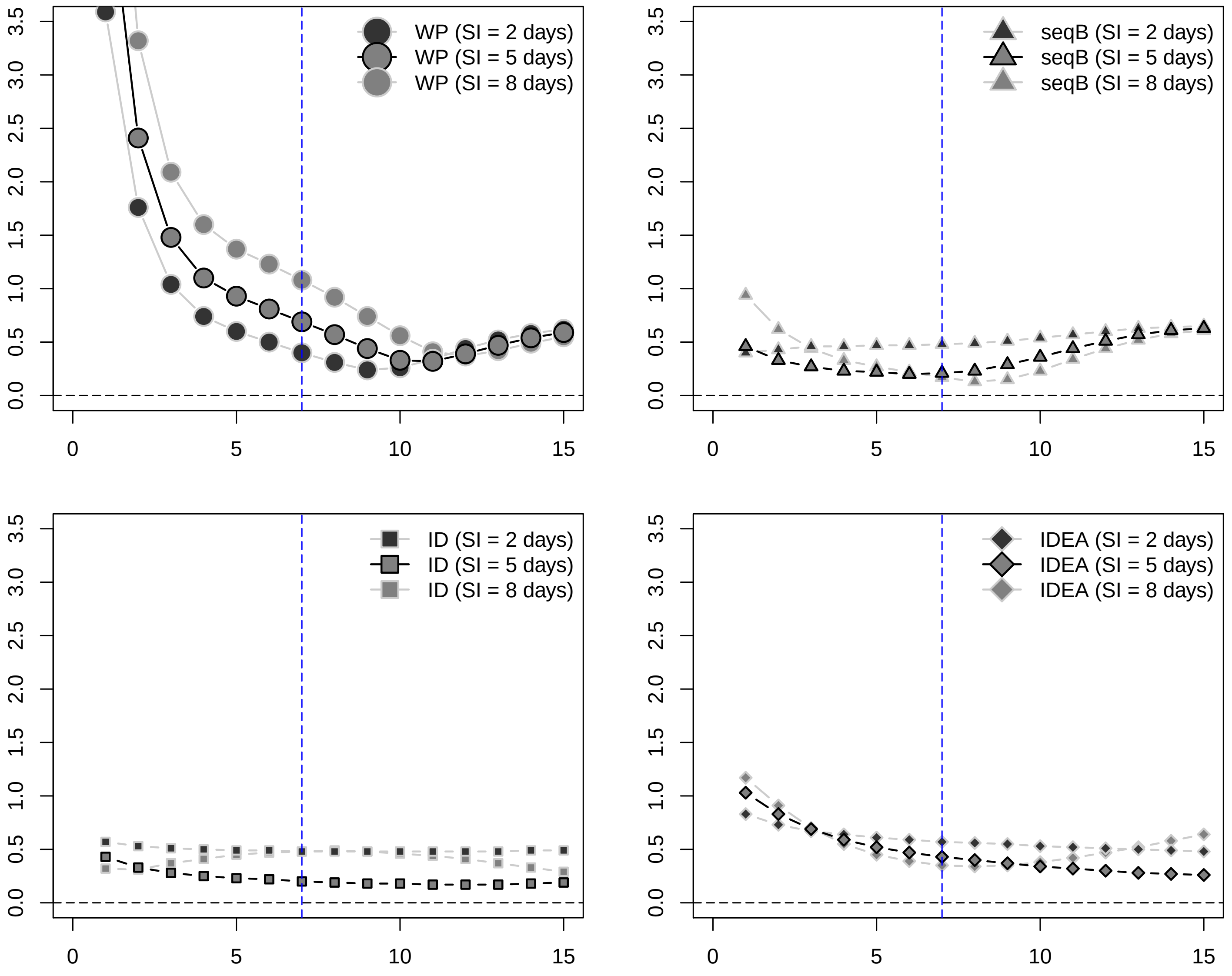

Figure 12: Influenza example 2 estimated MSE of WP, seqB, ID and IDEA R_0 estimators assuming known SI with SEAIR data (week on x-axis) under well-specified and misspecified cases of the serial interval, where the true serial interval is 5 days. The inflection point related to the epidemic data curve of the SEAIR model is indicated by the blue dashed vertical line.

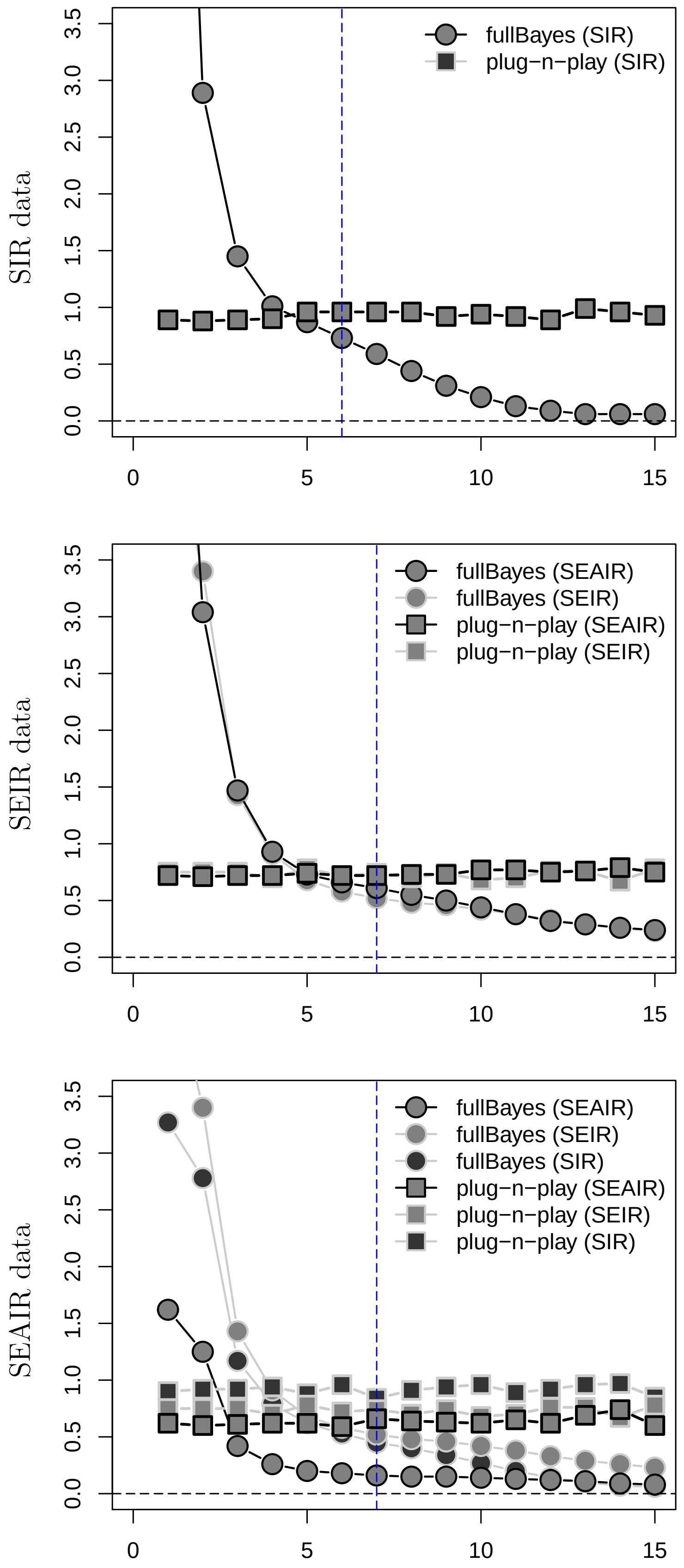

Figure 13: Influenza example 2 estimated MSE of fullBayes and plug-n-play R_0 estimators assuming unknown SI with SIR (top), SEIR (middle), SEAIR (bottom) data (week on x-axis). The inflection points related to the epidemic curves of the SIR, SEIR, and SEAIR models are indicated by the blue dashed vertical lines.

3.2 Boxplots - Influenza Example 2

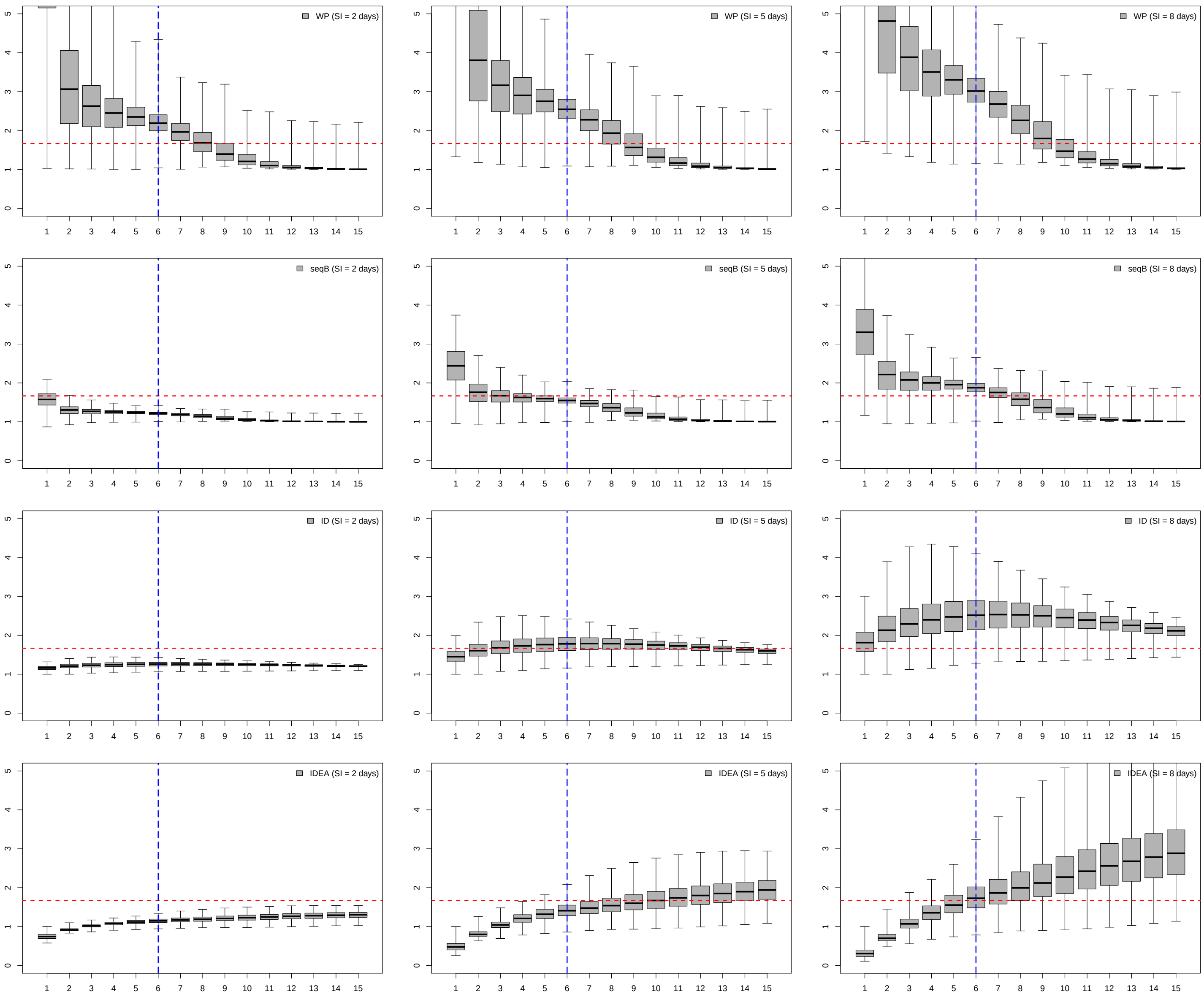

Figure 14: Influenza example 2 R_0 estimates assuming known SI with SIR data (week on x-axis) for the WP (top row), seqB (second), ID (third), IDEA (bottom) methods for known SI of 2 (left column), 5 (middle, true value), and 8 (right) days. the true R_0 is given by the red dashed horizontal line. The inflection point related to the epidemic data curve of the SIR model is indicated by the blue dashed vertical line.

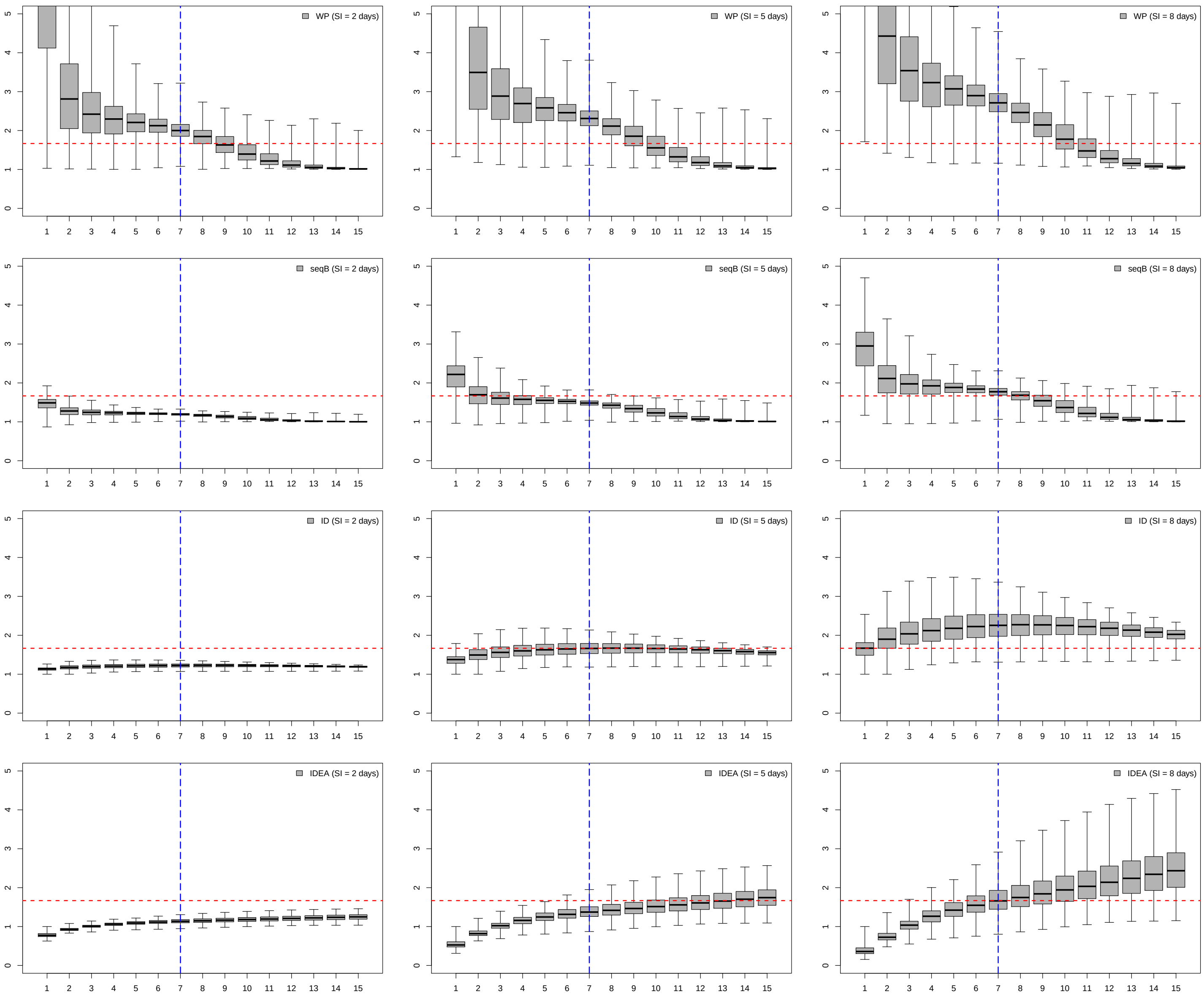

Figure 15: Influenza example 2 R_0 estimates assuming known SI with SEIR data (week on x-axis) for the WP (top row), seqB (second), ID (third), IDEA (bottom) methods for known SI of 2 (left column), 5 (middle, true value), and 8 (right) days. the true R_0 is given by the red dashed horizontal line. The inflection point related to the epidemic data curve of the SEIR model is indicated by the blue dashed vertical line.

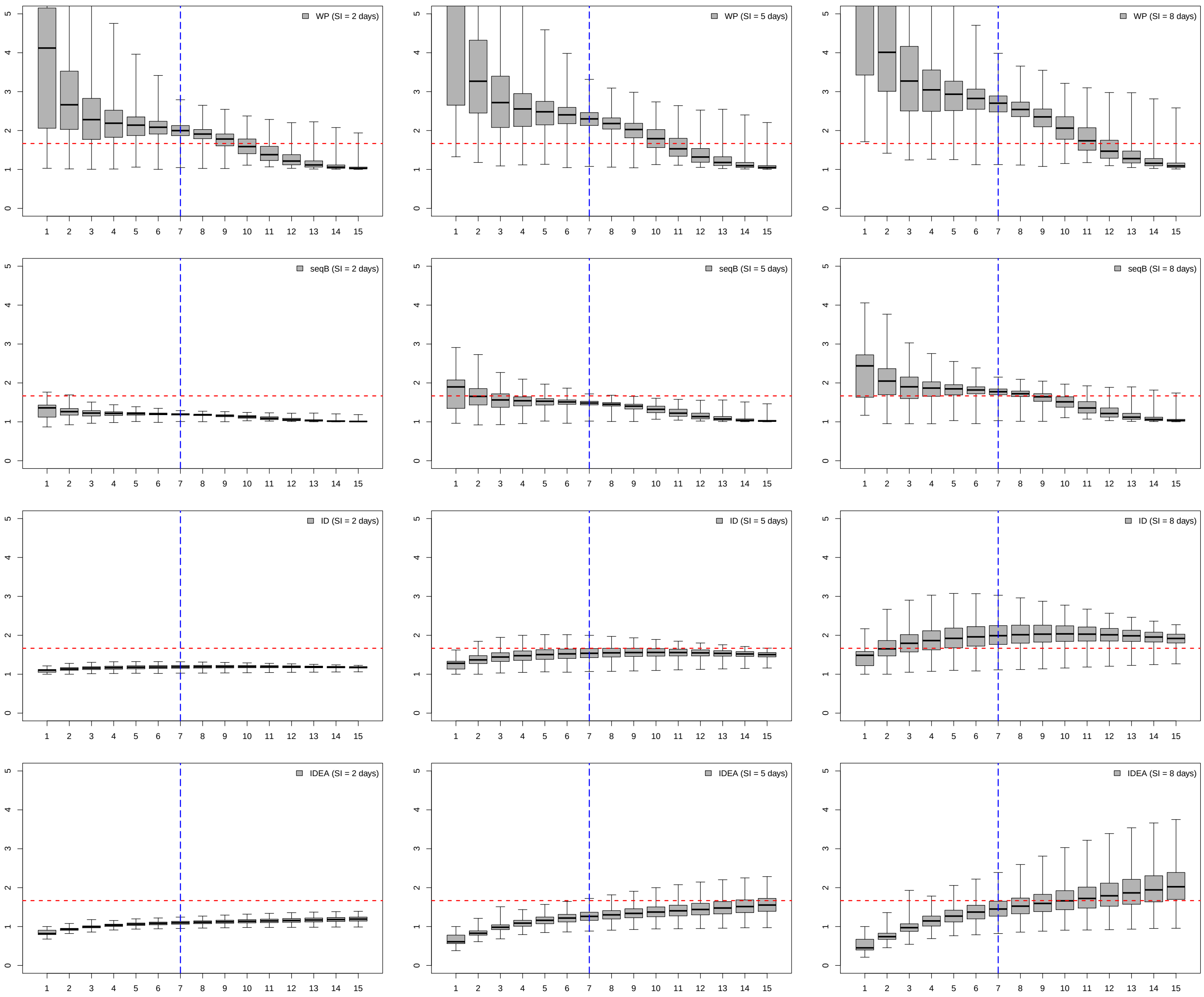

Figure 16: Influenza example 2 R_0 estimates assuming known SI with SEAIR data (week on x-axis) for the WP (top row), seqB (second), ID (third), IDEA (bottom) methods for known SI of 2 (left column), 5 (middle, true value), and 8 (right) days. the true R_0 is given by the red dashed horizontal line. The inflection point related to the epidemic data curve of the SEAIR model is indicated by the blue dashed vertical line.

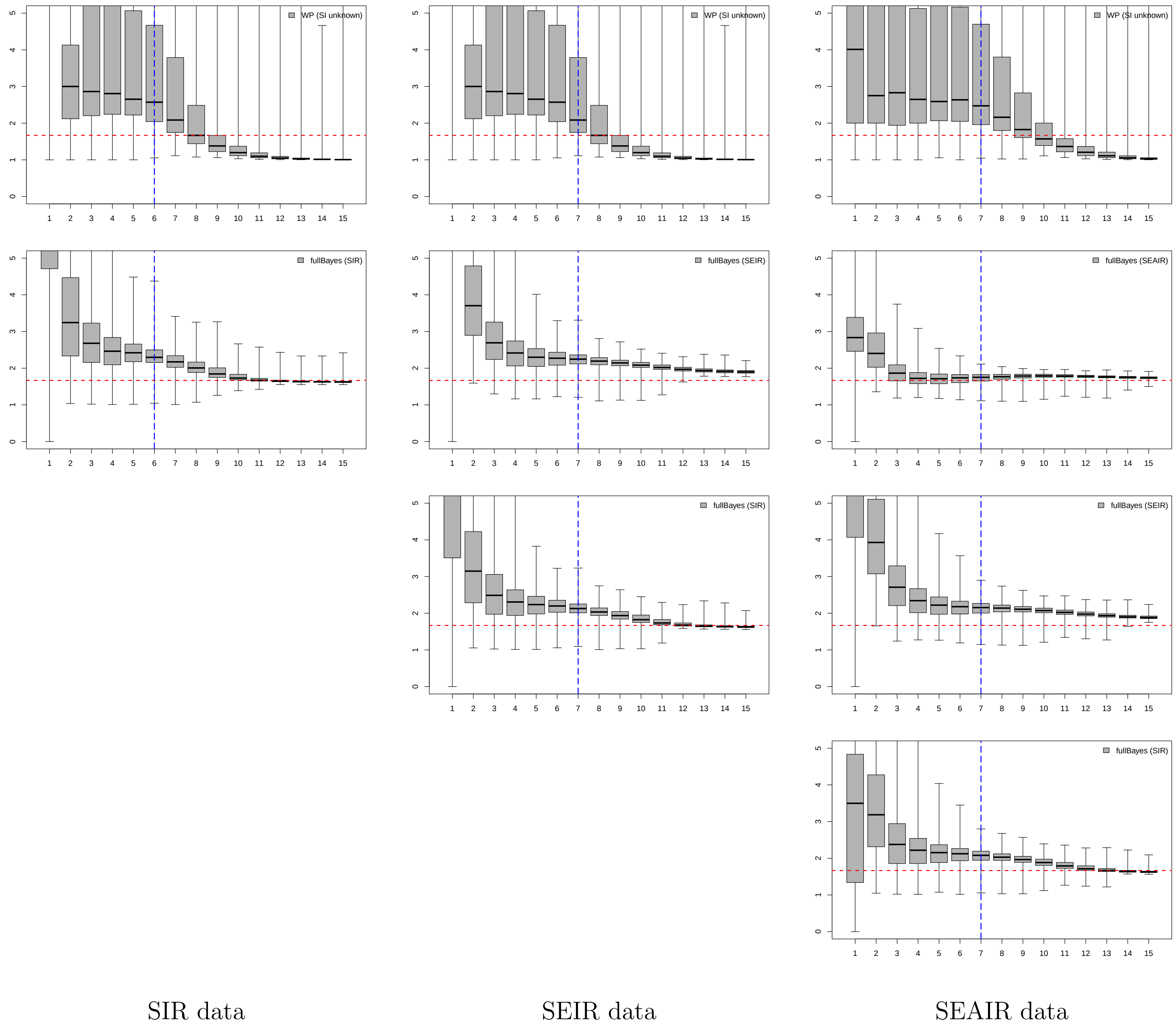

Figure 17: Influenza example 2 R_0 estimates assuming unknown SI for the WP and fullBayes methods (week on x-axis). The WP method is applied to SIR, SEIR, and SEAIR data. The fullBayes method assumes SIR, SEIR and SEAIR structures (rows), and is applied to SIR, SEIR, and SEAIR data (columns) with increasing dimension. The true R_0 is given by the red dashed horizontal line. The inflection points related to the epidemic data curves of the SIR, SEIR, and SEAIR models are indicated by the blue dashed vertical lines.

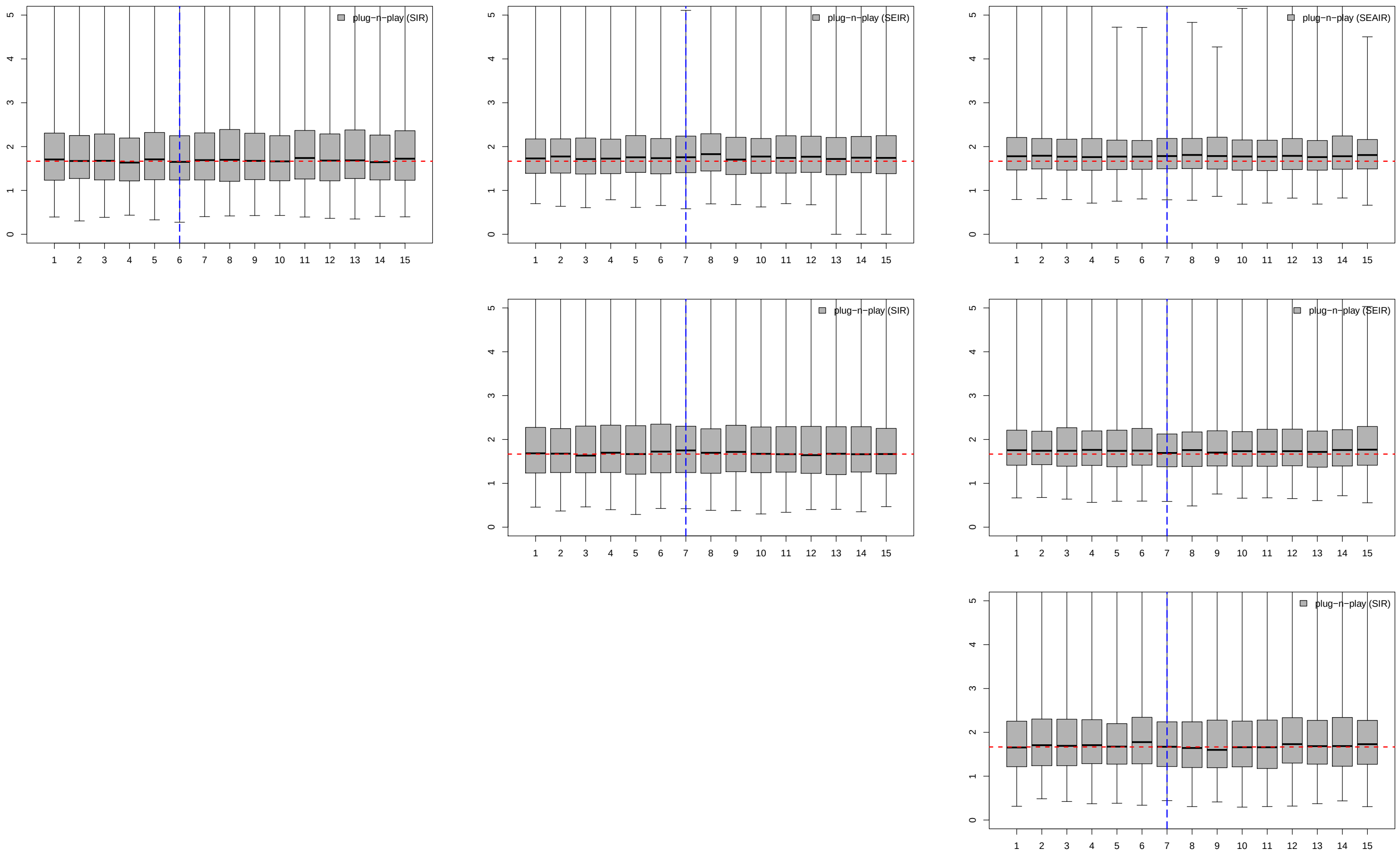

SIR data SEIR data SEAIR data

Figure 18: Influenza example 2 R_0 estimates assuming unknown SI for the plug-n-play method (week on x-axis), assuming SIR, SEIR and SEAIR model structures (rows), applied to SIR, SEIR, and SEAIR data (columns) with increasing dimension. The true R_0 is given by the red dashed horizontal line. The inflection points related to the epidemic data curve of the SIR, SEIR, and SEAIR models are indicated by the blue dashed vertical lines.

3.3 Tables - Influenza Example 2

Table 9: Influenza example 2: WP, seqB, ID and IDEA methods applied to SIR data with known SI of 2, 5 (true value), 8 days. The true R_0 value is 5/3. Each row reports the median value and standard deviation (in brackets). NA count (if applicable) is the number of simulations that yield NA as a R_0 value. * (if applicable) denotes a standard deviation greater than one million.

	SI	Week 1	2	3	4	5	6	7	8	9	10	11	12	13	14	15
WP	2 days	8.2 (6.5)	3.1 (1.6)	2.6 (0.9)	2.4 (0.6)	2.3 (0.4)	2.2 (0.3)	2 (0.3)	1.7 (0.3)	1.4 (0.3)	1.2 (0.3)	1.1 (0.2)	1.1 (0.1)	1.0 (0.1)	1.0 (0.1)	1.0 (0.1)
	5 days	10.6 (8.4)	3.8 (2)	3.2 (1.1)	2.9 (0.7)	2.8 (0.5)	2.5 (0.4)	2.3 (0.4)	1.9 (0.4)	1.6 (0.4)	1.3 (0.3)	1.2 (0.3)	1.1 (0.2)	1.0 (0.1)	1.0 (0.1)	1.0 (0.1)
	8 days	13.7 (10.9)	4.8 (2.5)	3.9 (1.3)	3.5 (0.9)	3.3 (0.7)	3 (0.5)	2.7 (0.5)	2.3 (0.5)	1.8 (0.5)	1.5 (0.4)	1.3 (0.3)	1.1 (0.3)	1.1 (0.2)	1.0 (0.1)	1.0 (0.1)
seqB	2 days	1.6 (0.2)	1.3 (0.1)	1.3 (0.1)	1.2 (0.1)	1.2 (0.1)	1.2 (0)	1.2 (0)	1.1 (0.1)	1.1 (0.1)	1.1 (0.1)	1.0 (0.0)	1.0 (0.0)	1.0 (0.0)	1.0 (0.0)	1.0 (0.0)
	5 days	2.4 (0.5)	1.8 (0.3)	1.7 (0.2)	1.6 (0.2)	1.6 (0.1)	1.5 (0.1)	1.5 (0.1)	1.4 (0.1)	1.2 (0.1)	1.1 (0.1)	1.1 (0.1)	1.0 (0.1)	1.0 (0.1)	1.0 (0.0)	1.0 (0.0)
	8 days	3.3 (0.8)	2.2 (0.5)	2.1 (0.4)	2.0 (0.3)	2.0 (0.2)	1.9 (0.2)	1.8 (0.2)	1.6 (0.2)	1.4 (0.2)	1.2 (0.2)	1.1 (0.2)	1.1 (0.1)	1.0 (0.1)	1.0 (0.1)	1.0 (0.0)
ID	2 days	1.2 (0.1)	1.2 (0.1)	1.2 (0.1)	1.2 (0.1)	1.3 (0.1)	1.3 (0.1)	1.3 (0.1)	1.3 (0.1)	1.3 (00)	1.3 (00)	1.2 (00)	1.2 (00)	1.2 (00)	1.2 (00)	1.2 (0.0)
	5 days	1.4 (0.2)	1.6 (0.2)	1.7 (0.2)	1.7 (0.2)	1.8 (0.2)	1.8 (0.2)	1.8 (0.2)	1.8 (0.2)	1.8 (0.2)	1.8 (0.2)	1.7 (0.1)	1.7 (0.1)	1.7 (0.1)	1.6 (0.1)	1.6 (0.1)
	8 days	1.8 (0.4)	2.1 (0.5)	2.3 (0.5)	2.4 (0.5)	2.5 (0.5)	2.5 (0.5)	2.5 (0.5)	2.5 (0.4)	2.5 (0.4)	2.5 (0.3)	2.4 (0.3)	2.3 (0.3)	2.3 (0.2)	2.2 (0.2)	2.1 (0.2)
IDEA	2 days	0.7 (0.1)	0.9 (0)	1 (0)	1.1 (0.1)	1.1 (0.1)	1.1 (0.1)	1.2 (0.1)	1.2 (0.1)	1.2 (0.1)	1.2 (0.1)	1.2 (0.1)	1.3 (0.1)	1.3 (0.1)	1.3 (0.1)	1.3 (0.1)
	5 days	0.5 (0.1)	0.8 (0.1)	1.0 (0.1)	1.2 (0.1)	1.3 (0.2)	1.4 (0.2)	1.5 (0.2)	1.5 (0.3)	1.6 (0.3)	1.7 (0.3)	1.7 (0.3)	1.8 (0.3)	1.9 (0.3)	1.9 (0.3)	1.9 (0.3)
	8 days	0.3 (0.2)	0.7 (0.1)	1.1 (0.2)	1.4 (0.2)	1.6 (0.3)	1.7 (0.4)	1.9 (0.5)	2 (0.6)	2.1 (0.6)	2.3 (0.7)	2.4 (0.7)	2.6 (0.8)	2.7 (0.8)	2.8 (0.8)	2.9 (0.8)

Table 10: Influenza example 2: WP, seqB, ID and IDEA methods applied to SEIR data with known SI of 2, 5 (true value), 8 days. The true R_0 value is 5/3. Each row reports the median value and standard deviation (in brackets). NA count (if applicable) is the number of simulations that yield NA as a R_0 value. * (if applicable) denotes a standard deviation greater than one million.

		Week														
	SI	**1**	**2**	**3**	**4**	**5**	**6**	**7**	8	9	10	11	12	13	14	15
WP	2 days	6.2<br>(3.9)	2.8<br>(1.3)	2.4<br>(0.8)	2.3<br>(0.5)	2.2<br>(0.4)	2.1<br>(0.3)	2<br>(0.3)	1.8<br>(0.2)	1.6<br>(0.3)	1.4<br>(0.3)	1.2<br>(0.2)	1.1<br>(0.2)	1.1<br>(0.2)	1.0<br>(0.1)	1.0<br>(0.1)
	5 days	8.0<br>(5.1)	3.5<br>(1.6)	2.9<br>(1)	2.7<br>(0.7)	2.6<br>(0.5)	2.5<br>(0.4)	2.3<br>(0.3)	2.1<br>(0.3)	1.9<br>(0.3)	1.6<br>(0.3)	1.3<br>(0.3)	1.2<br>(0.3)	1.1<br>(0.2)	1.0<br>(0.2)	1.0<br>(0.1)
	8 days	10.3<br>(6.5)	4.4<br>(2)	3.5<br>(1.2)	3.2<br>(0.8)	3.1<br>(0.6)	2.9<br>(0.5)	2.7<br>(0.4)	2.5<br>(0.4)	2.1<br>(0.4)	1.8<br>(0.4)	1.5<br>(0.4)	1.3<br>(0.3)	1.2<br>(0.3)	1.1<br>(0.2)	1.0<br>(0.2)
seqB	2 days	1.5<br>(0.2)	1.3<br>(0.1)	1.2<br>(0.1)	1.2<br>(0.1)	1.2<br>(0.1)	1.2<br>(0)	1.2<br>(0)	1.2<br>(0)	1.1<br>(0)	1.1<br>(0.1)	1.1<br>(0.0)	1.0<br>(0.0)	1.0<br>(0.0)	1.0<br>(0.0)	1.0<br>(0.0)
	5 days	2.2<br>(0.5)	1.7<br>(0.3)	1.6<br>(0.2)	1.6<br>(0.2)	1.6<br>(0.1)	1.5<br>(0.1)	1.5<br>(0.1)	1.4<br>(0.1)	1.3<br>(0.1)	1.2<br>(0.1)	1.1<br>(0.1)	1.1<br>(0.1)	1.0<br>(0.1)	1.0<br>(0.1)	1.0<br>(0.0)
	8 days	2.9<br>(0.7)	2.1<br>(0.5)	2<br>(0.4)	1.9<br>(0.3)	1.9<br>(0.2)	1.8<br>(0.2)	1.8<br>(0.1)	1.7<br>(0.2)	1.5<br>(0.2)	1.4<br>(0.2)	1.2<br>(0.2)	1.1<br>(0.2)	1.1<br>(0.1)	1.0<br>(0.1)	1.0<br>(0.1)
ID	2 days	1.1<br>(0.0)	1.2<br>(0.1)	1.2<br>(0.1)	1.2<br>(0.1)	1.2<br>(0.1)	1.2<br>(0.1)	1.2<br>(0.1)	1.2<br>(0.1)	1.2<br>(0.0)	1.2<br>(0.0)	1.2<br>(0.0)	1.2<br>(0.0)	1.2<br>(0.0)	1.2<br>(0.0)	1.2<br>(0.0)
	5 days	1.4<br>(0.1)	1.5<br>(0.2)	1.6<br>(0.2)	1.6<br>(0.2)	1.6<br>(0.2)	1.6<br>(0.2)	1.7<br>(0.2)	1.7<br>(0.2)	1.7<br>(0.2)	1.7<br>(0.1)	1.6<br>(0.1)	1.6<br>(0.1)	1.6<br>(0.1)	1.6<br>(0.1)	1.6<br>(0.1)
	8 days	1.7<br>(0.3)	1.9<br>(0.4)	2<br>(0.4)	2.1<br>(0.4)	2.2<br>(0.4)	2.2<br>(0.4)	2.3<br>(0.4)	2.3<br>(0.4)	2.3<br>(0.3)	2.3<br>(0.3)	2.2<br>(0.3)	2.2<br>(0.2)	2.1<br>(0.2)	2.1<br>(0.2)	2<br>(0.2)
IDEA	2 days	0.8<br>(0.1)	0.9<br>(0.0)	1<br>(0.0)	1.1<br>(0.0)	1.1<br>(0.1)	1.1<br>(0.1)	1.1<br>(0.1)	1.1<br>(0.1)	1.2<br>(0.1)	1.2<br>(0.1)	1.2<br>(0.1)	1.2<br>(0.1)	1.2<br>(0.1)	1.2<br>(0.1)	1.2<br>(0.1)
	5 days	0.5<br>(0.1)	0.8<br>(0.1)	1<br>(0.1)	1.2<br>(0.1)	1.2<br>(0.1)	1.3<br>(0.2)	1.4<br>(0.2)	1.4<br>(0.2)	1.5<br>(0.2)	1.5<br>(0.2)	1.6<br>(0.2)	1.6<br>(0.3)	1.7<br>(0.3)	1.7<br>(0.3)	1.7<br>(0.3)
	8 days	0.4<br>(0.2)	0.7<br>(0.1)	1<br>(0.2)	1.3<br>(0.2)	1.4<br>(0.3)	1.5<br>(0.3)	1.7<br>(0.4)	1.7<br>(0.4)	1.8<br>(0.4)	1.9<br>(0.5)	2<br>(0.5)	2.1<br>(0.6)	2.2<br>(0.6)	2.3<br>(0.6)	2.4<br>(0.6)

Table 11: Influenza example 2: WP, seqB, ID and IDEA methods applied to SEAIR data with known SI of 2, 5 (true value), 8 days. The true R_0 value is 5/3. Each row reports the median value and standard deviation (in brackets). NA count (if applicable) is the number of simulations that yield NA as a R_0 value. * (if applicable) denotes a standard deviation greater than one million.

		Week														
	SI	**1**	**2**	**3**	**4**	**5**	**6**	**7**	8	9	10	11	12	13	14	15
WP	2 days	4.1 (2.4)	2.7 (1.3)	2.3 (0.8)	2.2 (0.5)	2.1 (0.4)	2.1 (0.3)	2 (0.2)	1.9 (0.2)	1.8 (0.2)	1.6 (0.2)	1.4 (0.2)	1.2 (0.2)	1.1 (0.2)	1.1 (0.1)	1.0 (0.1)
	5 days	5.3 (3.1)	3.3 (1.6)	2.7 (0.9)	2.6 (0.6)	2.5 (0.5)	2.4 (0.4)	2.3 (0.3)	2.2 (0.2)	2.0 (0.3)	1.8 (0.3)	1.5 (0.3)	1.3 (0.3)	1.2 (0.2)	1.1 (0.2)	1.1 (0.1)
	8 days	6.9 (4.1)	4 (1.9)	3.3 (1.2)	3 (0.8)	2.9 (0.6)	2.8 (0.5)	2.7 (0.4)	2.5 (0.3)	2.4 (0.3)	2.1 (0.4)	1.7 (0.4)	1.5 (0.4)	1.3 (0.3)	1.2 (0.2)	1.1 (0.2)
seqB	2 days	1.4 (0.2)	1.3 (0.1)	1.2 (0.1)	1.2 (0.1)	1.2 (0.1)	1.2 (0.0)	1.2 (.00)	1.2 (0.0)	1.2 (0.0)	1.1 (0.0)	1.1 (0.0)	1.1 (0.0)	1.0 (0.0)	1.0 (0.0)	1.0 (0.0)
	5 days	1.9 (0.4)	1.7 (0.3)	1.6 (0.2)	1.5 (0.2)	1.5 (0.1)	1.5 (0.1)	1.5 (0.1)	1.5 (0.1)	1.4 (0.1)	1.3 (0.1)	1.2 (0.1)	1.1 (0.1)	1.1 (0.1)	1.0 (0.1)	1.0 (0.1)
	8 days	2.4 (0.7)	2 (0.5)	1.9 (0.4)	1.9 (0.3)	1.8 (0.2)	1.8 (0.2)	1.8 (0.1)	1.7 (0.1)	1.6 (0.1)	1.5 (0.2)	1.4 (0.2)	1.2 (0.2)	1.1 (0.1)	1.1 (0.1)	1.0 (0.1)
ID	2 days	1.1 (0.0)	1.1 (0.1)	1.2 (0.1)	1.2 (0.1)	1.2 (0.1)	1.2 (0.1)	1.2 (0.1)	1.2 (0.0)	1.2 (0.0)	1.2 (0.0)	1.2 (0.0)	1.2 (0.0)	1.2 (0.0)	1.2 (0.0)	1.2 (0.0)
	5 days	1.3 (0.1)	1.4 (0.2)	1.4 (0.2)	1.5 (0.2)	1.5 (0.2)	1.5 (0.2)	1.5 (0.2)	1.5 (0.2)	1.6 (0.1)	1.6 (0.1)	1.6 (0.1)	1.5 (0.1)	1.5 (0.1)	1.5 (0.1)	1.5 (0.1)
	8 days	1.5 (0.2)	1.7 (0.3)	1.8 (0.3)	1.9 (0.4)	1.9 (0.4)	2.0 (0.3)	2.0 (0.3)	2.0 (0.3)	2.0 (0.3)	2.0 (0.3)	2.0 (0.3)	2.0 (0.2)	2.0 (0.2)	2.0 (0.2)	1.9 (0.2)
IDEA	2 days	0.8 (0.1)	0.9 (0)	1 (0)	1 (0)	1.1 (0)	1.1 (0.1)	1.1 (0.1)	1.1 (0.1)	1.1 (0.1)	1.1 (0.1)	1.1 (0.1)	1.2 (0.1)	1.2 (0.1)	1.2 (0.1)	1.2 (0.1)
	5 days	0.6 (0.1)	0.8 (0.1)	1 (0.1)	1.1 (0.1)	1.2 (0.1)	1.2 (0.1)	1.3 (0.2)	1.3 (0.2)	1.3 (0.2)	1.4 (0.2)	1.4 (0.2)	1.4 (0.2)	1.5 (0.2)	1.5 (0.2)	1.6 (0.2)
	8 days	0.5 (0.2)	0.7 (0.1)	1 (0.2)	1.1 (0.2)	1.3 (0.2)	1.4 (0.3)	1.5 (0.3)	1.5 (0.3)	1.6 (0.3)	1.7 (0.4)	1.7 (0.4)	1.8 (0.4)	1.9 (0.5)	1.9 (0.5)	2.0 (0.5)

Table 12: Influenza example 2: WP, fullBayes, and plug-n-play methods applied ot SIR, SEIR, and SEAIR data, with SI unknown. The true R_0 values is 5/3. fullBayes and plug-n-play methods assume SIR, SEIR, or SEAIR model structures. Each row reports the median value and standard deviation (in brackets). NA count (if applicable) is the number of simulations that yield NA as a R_0 value. * (if applicable) denotes a standard deviation greater than one million.

		Week														
	data	1	2	3	4	5	6	7	8	9	10	11	12	13	14	15
WP	SIR	8.5 (639.4)	3.0 (4476.2)	2.9 (1516.9)	2.8 (1587.8)	2.7 (1402.7)	2.6 (808.9)	2.1 (83609.9)	1.7 (2933.4)	1.4 (990.6)	1.2 (212767.6)	1.1 (9826.7)	1.0 (1832.9)	1.0 (1278.9)	1.0 (0.1)	1.0 (1855.2)
	SEIR	8.1 (329.7)	2.8 (2092.9)	2.7 (16638.1)	2.5 (2229)	2.4 (2258.5)	2.4 (323.2)	2.2 (386.1)	1.9 (146.9)	1.6 (20234.5)	1.4 (5544.6)	1.2 (307.8)	1.1 (849)	1.1 (349.9)	1.0 (0.2)	1.0 (0.1)
	SEAIR	4.0 (3.0)	2.8 (38390.5)	2.8 (3818.9)	2.6 (348.7)	2.6 (665.2)	2.6 (838.7)	2.5 (326.7)	2.2 (85.1)	1.8 (2097)	1.6 (20.8)	1.4 (193.4)	1.2 (528.3)	1.1 (188)	1.1 (315.6)	1.0 (0.2)
plug-n-play (SIR)	SIR	1.7 (0.9)	1.7 (0.9)	1.7 (0.9)	1.6 (0.9)	1.7 (0.9)	1.7 (0.9)	1.7 (0.9)	1.7 (0.9)	1.7 (0.9)	1.7 (0.9)	1.7 (0.9)	1.7 (0.9)	1.7 (1.0)	1.6 (0.9)	1.7 (0.9)
	SEIR	1.7 (0.9)	1.7 (0.9)	1.6 (1)	1.7 (0.9)	1.7 (0.9)	1.7 (0.9)	1.7 (0.9)	1.7 (0.9)	1.7 (0.9)	1.7 (0.8)	1.7 (0.9)	1.6 (0.9)	1.7 (1)	1.7 (0.9)	1.7 (0.9)
	SEAIR	1.7 (0.9)	1.7 (0.9)	1.7 (0.9)	1.7 (0.9)	1.7 (0.9)	1.8 (0.9)	1.7 (0.8)	1.6 (0.9)	1.6 (0.9)	1.7 (0.9)	1.7 (0.9)	1.7 (0.9)	1.7 (0.9)	1.7 (0.9)	1.7 (0.8)
plug-n-play (SEIR)	SEIR	1.7 (0.7)	1.8 (0.7)	1.7 (0.7)	1.7 (0.7)	1.8 (0.7)	1.7 (0.7)	1.8 (0.7)	1.8 (0.7)	1.7 (0.7)	1.8 (0.7)	1.7 (0.7)	1.8 (0.7)	1.7 (0.7)	1.7 (0.7)	1.7 (0.7)
	SEAIR	1.8 (0.7)	1.7 (0.7)	1.7 (0.7)	1.8 (0.7)	1.7 (0.7)	1.7 (0.7)	1.7 (0.7)	1.8 (0.7)	1.7 (0.7)	1.7 (0.7)	1.7 (0.7)	1.7 (0.7)	1.7 (0.7)	1.8 (0.6)	1.8 (0.7)
plug-n-play (SEAIR)	SEAIR	1.8 0.6)	1.8 (0.6)	1.8 (0.6)	1.8 (0.6)	1.8 (0.6)	1.8 (0.6)	1.8 (0.6)	1.8 (0.6)	1.8 (0.6)	1.8 (0.6)	1.8 (0.6)	1.8 (0.6)	1.8 (0.7)	1.8 (0.7)	1.8 (0.6
fullBayes (SIR)	SIR	7.7 (6.5)	3.2 (2.1)	2.7 (0.9)	2.5 (0.6)	2.4 (0.4)	2.3 (0.3)	2.2 (0.3)	2 (0.2)	1.8 (0.2)	1.7 (0.2)	1.7 (0.1)	1.6 (0.1)	1.6 (0.1)	1.6 (0.0)	1.6 (0.0)
	SEIR	5.6 (4.0)	3.1 (1.8)	2.5 (0.9)	2.3 (0.5)	2.2 (0.4)	2.2 (0.3)	2.1 (0.2)	2 (0.2)	1.9 (0.2)	1.8 (0.2)	1.7 (0.1)	1.7 (0.1)	1.7 (0.1)	1.6 (0.1)	1. (0.0)
	SEAIR	3.5 (2.6)	3.2 (2)	2.4 (0.8)	2.2 (0.5)	2.2 (0.4)	2.1 (0.3)	2.1 (0.2)	2 (0.2)	2 (0.1)	1.9 (0.1)	1.8 (0.1)	1.7 (0.1)	1.7 (0.1)	1.6 (0.1)	1.6 (0.1)
fullBayes (SEIR)	SEIR	7.9 (3.9)	3.7 (1.8)	2.7 (0.9)	2.4 (0.5)	2.3 (0.4)	2.3 (0.3)	2.2 (0.3)	2.2 (0.2)	2.1 (0.1)	2.1 (0.1)	2 (0.1)	2 (0.1)	1.9 (0.1)	1.9 (0.1)	1.9 (0.1)
	SEAIR	5.3 (2.7)	3.9 (2.1)	2.7 (0.8)	2.3 (0.5)	2.2 (0.4)	2.2 (0.3)	2.2 (0.2)	2.1 (0.2)	2.1 (0.1)	2.1 (0.1)	2 (0.1)	2 (0.1)	1.9 (0.1)	1.9 (0.1)	1.9 (0.1)
fullBayes (SEAIR)	SEAIR	2.8 (1.1)	2.4 (0.8)	1.9 (0.4)	1.7 (0.2)	1.7 (0.2)	1.7 (0.2)	1.8 (0.1)	1.8 (0.1)	1.8 (0.1)	1.8 (0.1)	1.8 (0.1)	1.8 (0.0)	1.8 (0.0)	1.7 (0.0)	1.7 (0.0)

4 COVID-19 Example

Parameter values are listed in Table 2. COVID-19 parameters are such that $R_0 = 2.6$ and the serial interval is 5.2 days for the SIR, SEIR, and SEAIR models.

For the WP method, we obtain values of p_t, representing the serial distribution. The serial distribution values, discretized to weeks, from the SIR/SEIR/SEAIR model epidemic data for COVID-19 are

- 0.74 0.19 0.05 0.01
- 0.52 0.34 0.11 0.03 0.01
- 0.78 0.19 0.02

Given a known serial interval μ, we obtain the following serial distributions, discretized to weeks:

- ($\mu = 5.2$) 0.74 0.19 0.05 0.01
- ($\mu = 4.2$) 0.81 0.15 0.03 0.01
- ($\mu = 7.5$) 0.61 0.24 0.09 0.04 0.01 0.01

When the serial distribution is unknown, the WP method determines serial distributions. fullBayes and plug-n-play also return estimates of the serial distribution, for the SIR, SEIR, and SEAIR examples. Estimates of the serial distribution are shown in Table 13. Note that for the WP method, we make the standard actuarial mid-week assumption. That is, we assume that within a week, an event occurs on average in the middle of the week.

Table 13: COVID-19 example - estimating SI (value in weeks): WP, fullBayes, and plug-n-play methods applied ot SIR, SEIR, and SEAIR data, with SI unknown. fullBayes and plug-n-play methods assume SIR, SEIR, or SEAIR model structures. Each row reports the median value with standard deviation below in brackets. The true SI (in weeks) is for $5.2/7 = 0.74$ for all models.

		Week														
	data	1	2	3	4	5	6	7	8	9	10	11	12	13	14	15
WP (SI unknown)	SIR	0.5<br>(25.3)	0.5<br>(6.3)	9<br>(14.3)	1.9<br>(19.2)	0.5<br>(17.3)	0.5<br>(12.6)	0.5<br>(4)	0.5<br>(4.2)	0.5<br>(0.8)	0.5<br>(0.4)	0.5<br>(0)	0.5<br>(0)	0.5<br>(0)	0.5<br>(0)	0.5<br>(0)
	SEIR	0.5<br>(25.7)	0.5<br>(4.8)	3.1<br>(10.5)	8.9<br>(13.4)	3.1<br>(16.9)	0.7<br>(18.9)	0.5<br>(20.4)	0.5<br>(20.1)	0.5<br>(15)	0.5<br>(9.9)	0.5<br>(6.4)	0.5<br>(4.5)	0.5<br>(1.1)	0.5<br>(0)	0.5<br>(0)
	SEAIR	0.5<br>(14.5)	1.4<br>(7.5)	2.6<br>(10.9)	2.7<br>(11.4)	3.8<br>(11.2)	2.7<br>(11)	1.2<br>(10.5)	0.5<br>(10.5)	0.5<br>(10.5)	0.5<br>(9.6)	0.5<br>(8.2)	0.5<br>(4.9)	0.5<br>(4.9)	0.5<br>(1.3)	0.<br>(0)
plug-n-play (SIR)	SIR	0.8<br>(0.3)	0.8<br>(0.3)	0.8<br>(0.3)	0.8<br>(0.3)	0.8<br>(0.3)	0.8<br>(0.3)	0.8<br>(0.3)	0.8<br>(0.3)	0.8<br>(0.3)	0.8<br>(0.3)	0.8<br>(0.3)	0.7<br>(0.3)	0.8<br>(0.3)	0.8<br>(0.3)	0.8<br>(0.3)
	SEIR	0.8<br>(0.3)	0.8<br>(0.3)	0.8<br>(0.3)	0.8<br>(0.3)	0.8<br>(0.3)	0.8<br>(0.3)	0.7<br>(0.3)	0.8<br>(0.3)	0.8<br>(0.3)	0.8<br>(0.3)	0.8<br>(0.3)	0.8<br>(0.3)	0.8<br>(0.3)	0.8<br>(0.3)	0.8<br>(0.3)
	SEAIR	0.8<br>(0.3)	0.8<br>(0.3)	0.8<br>(0.3)	0.8<br>(0.3)	0.8<br>(0.3)	0.8<br>(0.3)	0.8<br>(0.3)	0.8<br>(0.3)	0.7<br>(0.3)	0.8<br>(0.3)	0.8<br>(0.3)	0.8<br>(0.3)	0.8<br>(0.3)	0.8<br>(0.3)	0.8<br>(0.3)
plug-n-play (SEIR)	SEIR	0.8<br>(0.2)	0.8<br>(0.2)	0.8<br>(0.2)	0.8<br>(0.2)	0.8<br>(0.2)	0.8<br>(0.2)	0.8<br>(0.2)	0.8<br>(0.2)	0.8<br>(0.2)	0.8<br>(0.2)	0.8<br>(0.2)	0.8<br>(0.2)	0.8<br>(0.2)	0.8<br>(0.2)	0.8<br>(0.2)
	SEAIR	0.8<br>(0.2)	0.8<br>(0.2)	0.8<br>(0.2)	0.8<br>(0.2)	0.8<br>(0.2)	0.8<br>(0.2)	0.8<br>(0.2)	0.8<br>(0.2)	0.8<br>(0.2)	0.8<br>(0.2)	0.8<br>(0.2)	0.8<br>(0.2)	0.8<br>(0.2)	0.8<br>(0.2)	0.8<br>(0.2)
plug-n-play (SEAIR)	SEAIR	0.8<br>(0.2)	0.8<br>(0.2)	0.8<br>(0.2)	0.8<br>(0.2)	0.8<br>(0.2)	0.8<br>(0.2)	0.8<br>(0.2)	0.8<br>(0.2)	0.8<br>(0.2)	0.8<br>(0.2)	0.8<br>(0.2)	0.8<br>(0.2)	0.8<br>(0.2)	0.8<br>(0.2)	0.8<br>(0.2)
fullBayes (SIR)	SIR	3.8<br>(3.7)	3.5<br>(2)	3.9<br>(1.2)	3.1<br>(0.9)	1.6<br>(0.9)	1.1<br>(0.5)	1<br>(0.2)	1<br>(0.1)	1<br>(0)	1<br>(0)	1<br>(0)	1<br>(0)	1<br>(0)	1<br>(0)	1<br>(0)
	SEIR	1.9<br>(1.3)	2.1<br>(1.1)	2.4<br>(0.8)	2.6<br>(0.6)	2.5<br>(0.5)	2.1<br>(0.6)	1.5<br>(0.6)	1.1<br>(0.4)	1<br>(0.3)	1<br>(0.2)	1<br>(0.1)	1<br>(0)	1<br>(0)	1<br>(0)	1<br>(0)
	SEAIR	1.6<br>(0.5)	2.2<br>(0.9)	2.1<br>(0.6)	2.1<br>(0.3)	2<br>(0.2)	1.9<br>(0.2)	1.8<br>(0.2)	1.5<br>(0.2)	1.2<br>(0.2)	1.1<br>(0.2)	1<br>(0.1)	1<br>(0.1)	1<br>(0)	1<br>(0)	1<br>(0)
fullBayes (SEIR)	SEIR	1<br>(0.3)	1.8<br>(0.8)	2.7<br>(0.7)	3.3<br>(0.5)	3.3<br>(0.3)	3.1<br>(0.4)	2.9<br>(0.3)	3.1<br>(0.5)	3.7<br>(0.8)	4.6<br>(1)	5.6<br>(1.1)	6.6<br>(1.2)	7.5<br>(1.2)	8.3<br>(1.1)	9.3<br>(1.2)
	SEAIR	0.8<br>(0.1)	1.6<br>(0.4)	2.5<br>(0.4)	2.9<br>(0.3)	3<br>(0.1)	2.9<br>(0.1)	2.9<br>(0.1)	2.8<br>(0.1)	3<br>(0.2)	3.4<br>(0.4)	4.2<br>(0.6)	5<br>(0.8)	6<br>(0.9)	6.9<br>(0.9)	7.9<br>(0.9)
fullBayes (SEAIR)	SEAIR	0.8<br>(0.1)	0.9<br>(0.2)	0.9<br>(0.1)	1<br>(0.2)	1.4<br>(0.2)	1.5<br>(0.2)	1.5<br>(0.1)	1.5<br>(0.1)	1.4<br>(0.1)	1.4<br>(0.1)	1.3<br>(0)	1.3<br>(0)	1.3<br>(0)	1.3<br>(0)	1.3<br>(0)

4.1 MSE - COVID-19 Example

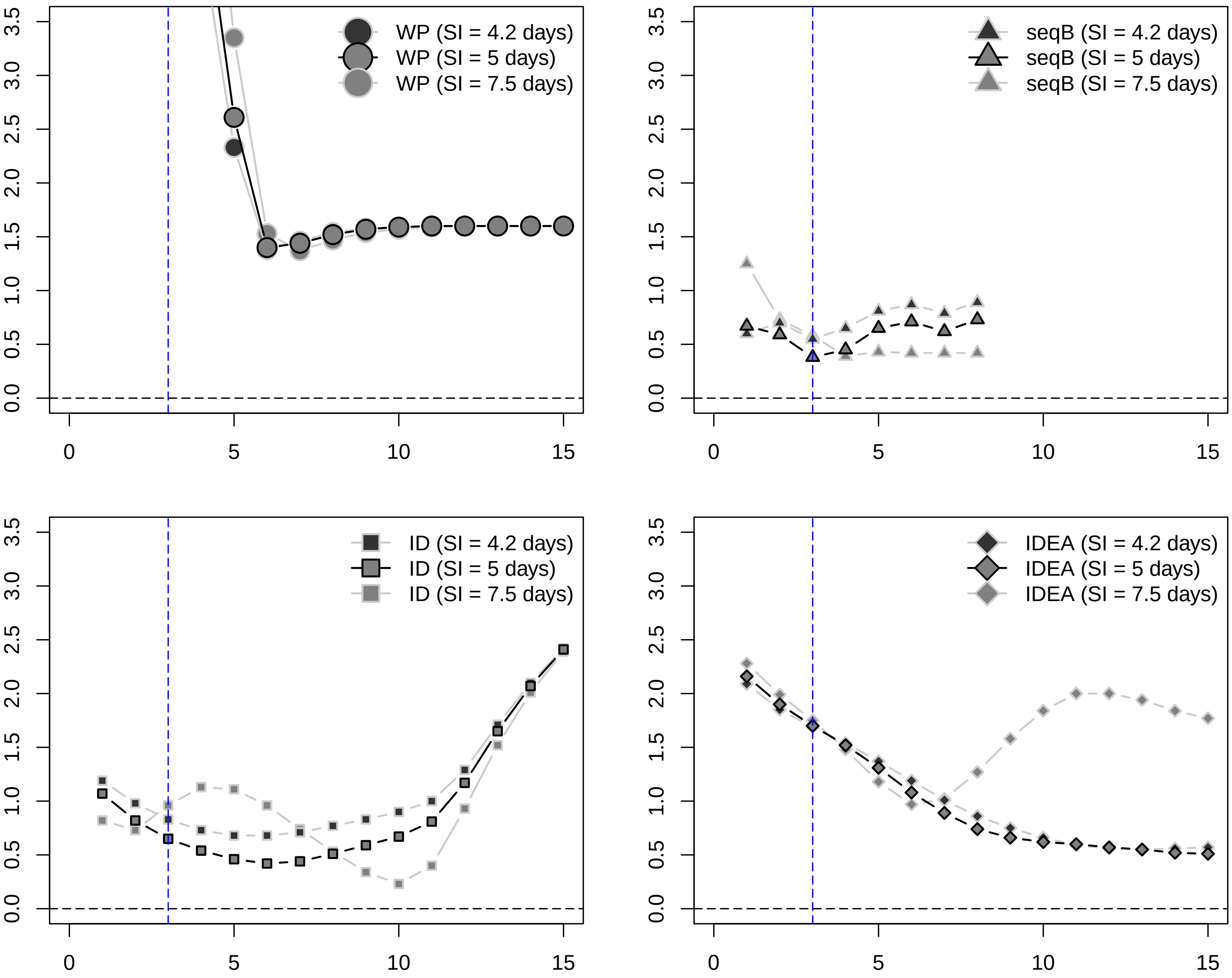

Figure 19: COVID-19 estimated MSE of WP, seqB, ID, and IDEA R0 estimators assuming known SI with SIR data (week on x-axis) under well-specified and misspecified cases of the serial interval, where the true serial interval is 5 days. The inflection point related to the epidemic data curve of the SIR model is indicated by the blue dashed vertical line.

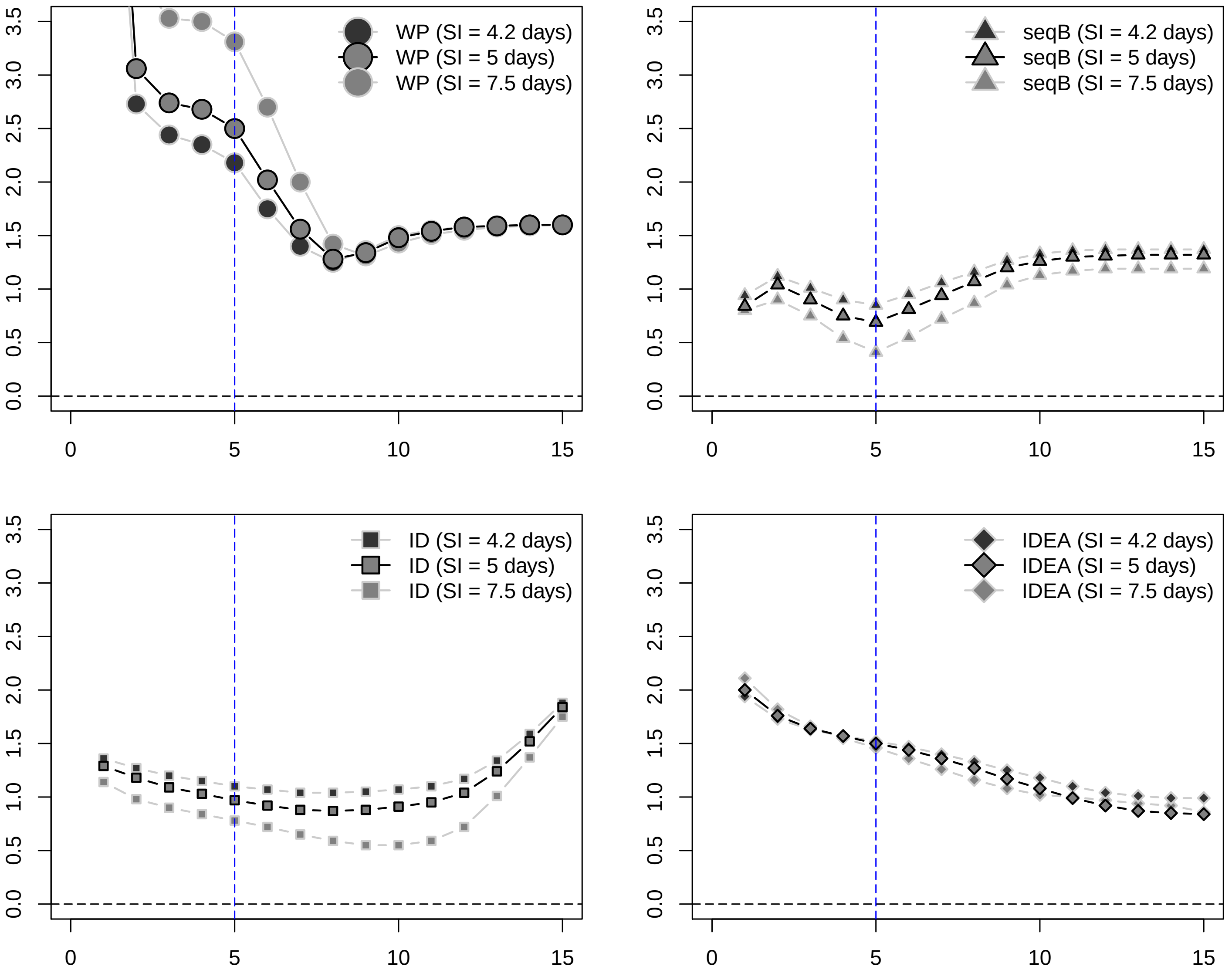

Figure 20: COVID-19 estimated MSE of WP, seqB, ID, and IDEA R0 estimators assuming known SI with SEIR data (week on x-axis) under well-specified and misspecified cases of the serial interval, where the true serial interval is 5 days. The inflection point related to the epidemic data curve of the SEIR model is indicated by the blue dashed vertical line.

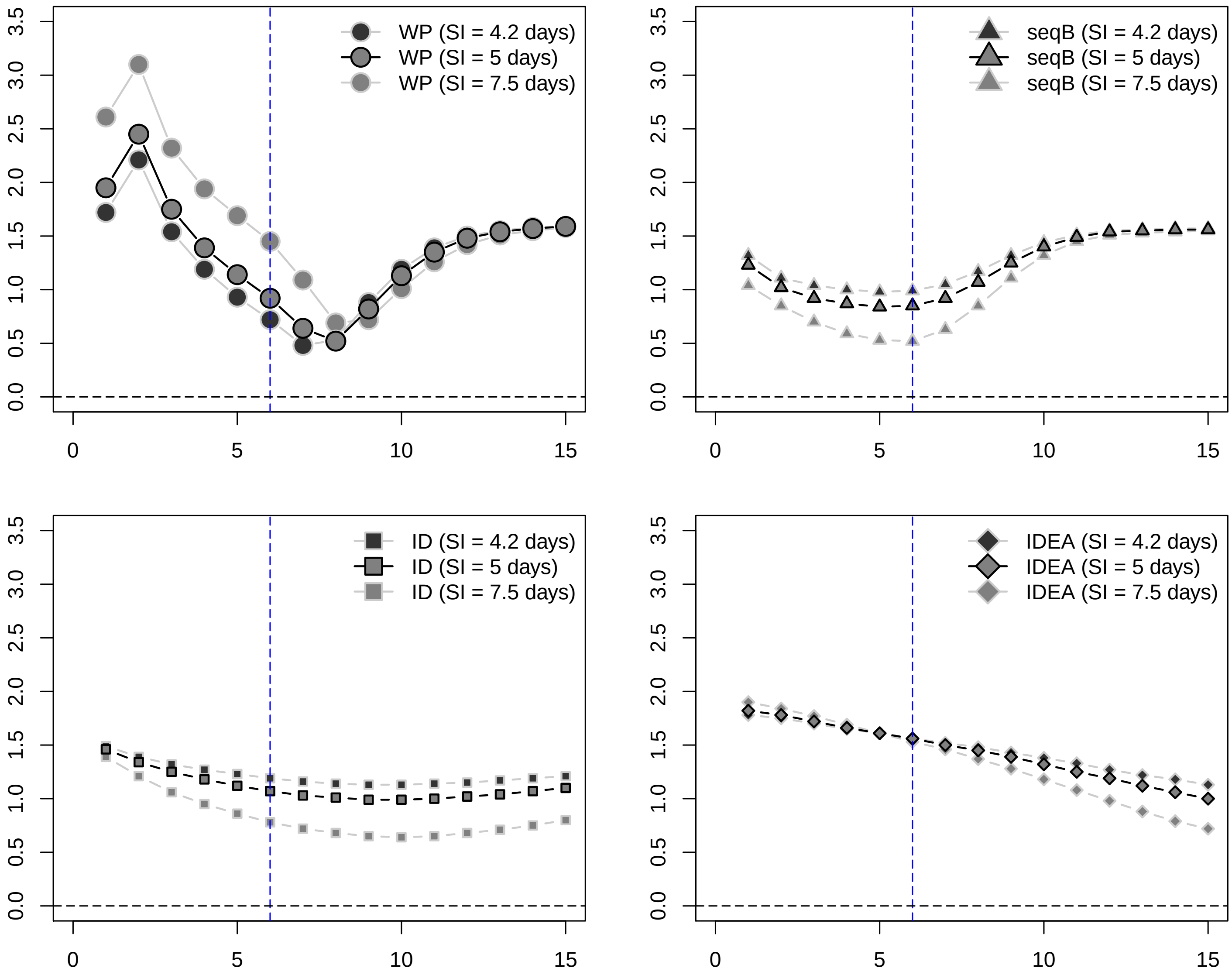

Figure 21: COVID-19 estimated MSE of WP, seqB, ID, and IDEA R0 estimators assuming known SI with SEAIR data (week on x-axis) under well-specified and misspecified cases of the serial interval, where the true serial interval is 5 days. The inflection point related to the epidemic data curve of the SEAIR model is indicated by the blue dashed vertical line.

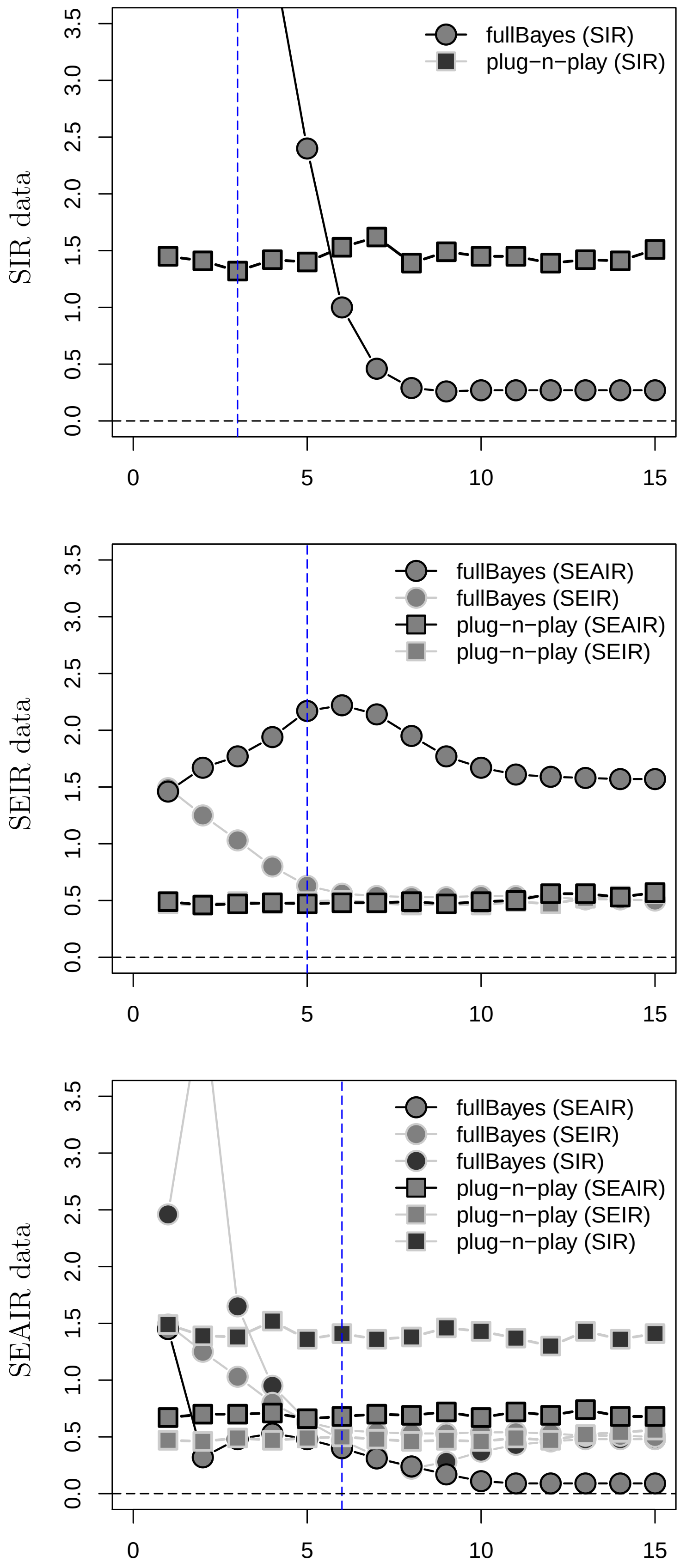

Figure 22: COVID-19 estimated MSE of fullBayes and plug-n-play R0 estimators assuming unknown SI with SIR (top), SEIR (middle) and SEAIR (bottom) data (week on x-axis). The inflection points related to the epidemic data curve of the SIR, SEIR and SEAIR models are indicated by the blue dashed vertical lines.

4.2 Boxplots - COVID-19 Example

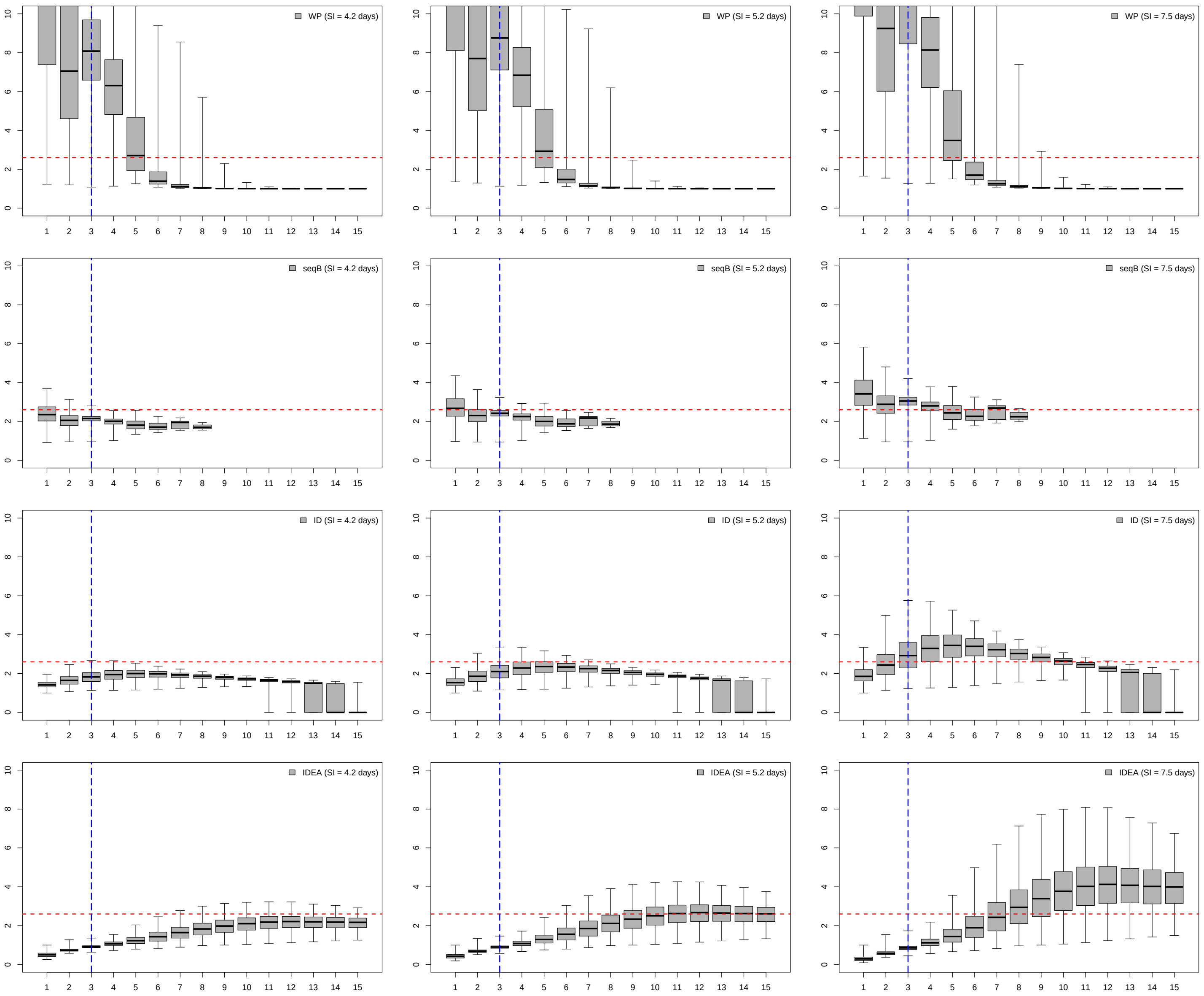

Figure 23: COVID-19 R0 estimates assuming known SI with SIR data (week on x-axis) for the WP (top row), seqB (second), ID (third), and IDEA (bottom) methods for known SI of 4.2 (left column), 5.2 (middle, true value), and 7.5 (right) days. The true R0 is given by the red dashed horizontal line. The inflection point related to the epidemic data curve of the SIR model is indicated by the blue dashed vertical lines.

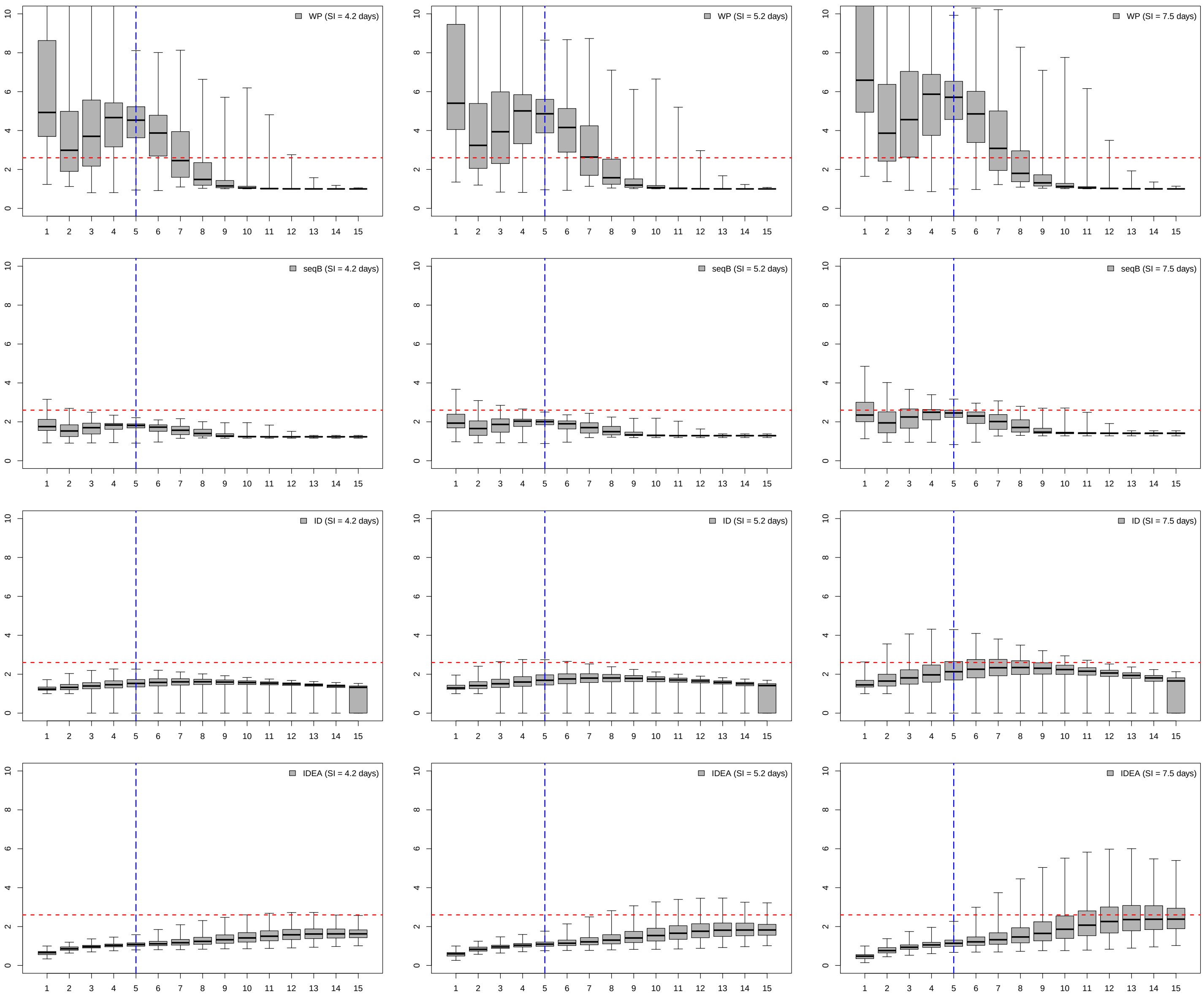

Figure 24: COVID-19 R0 estimates assuming known SI with SEIR data (week on x-axis) for the WP (top row), seqB (second), ID (third), and IDEA (bottom) methods for known SI of 4.2 (left column), 5.2 (middle, true value), and 7.5 (right) days. The true R0 is given by the red dashed horizontal line. The inflection point related to the epidemic data curve of the SEIR model is indicated by the blue dashed vertical lines.

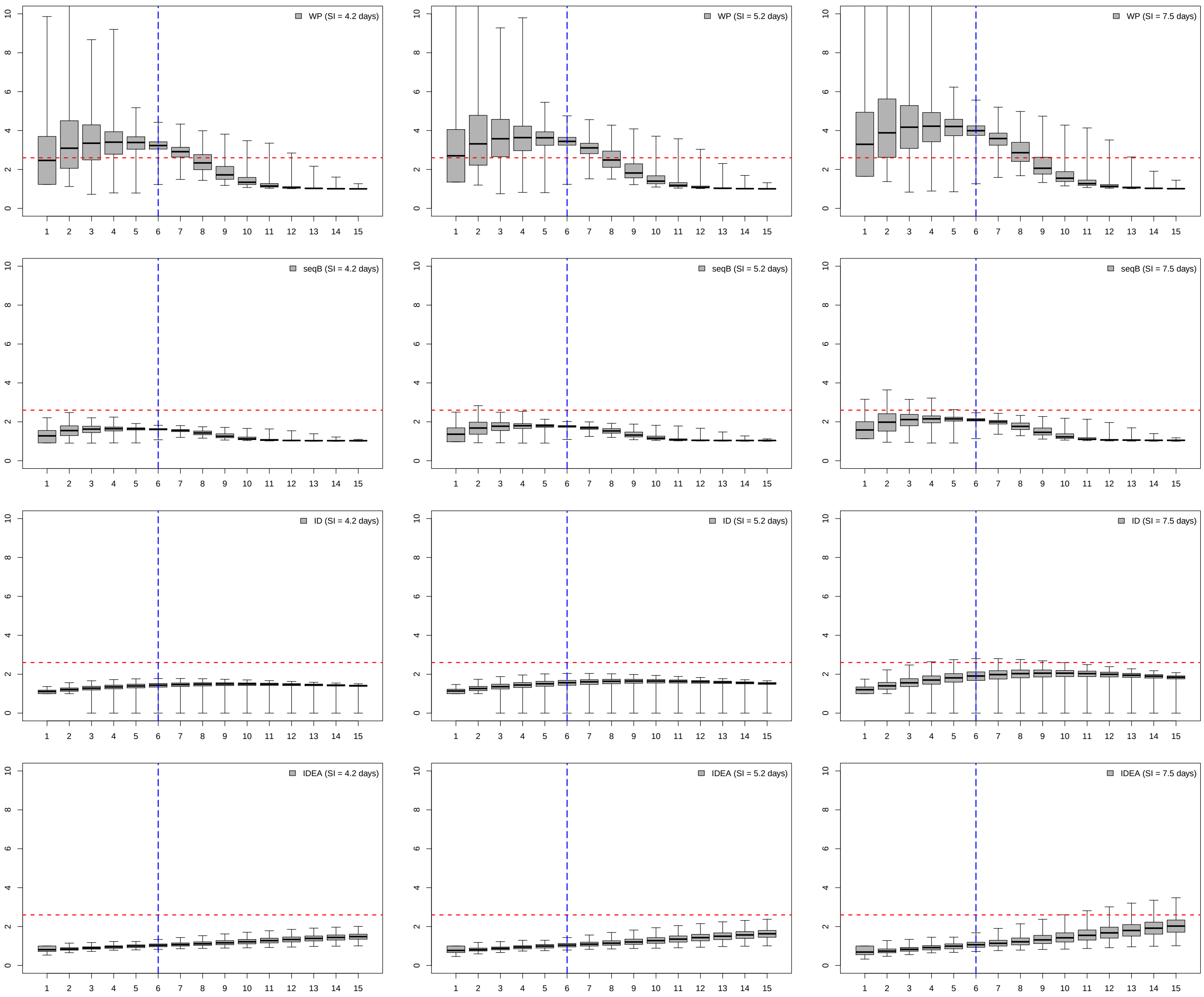

Figure 25: COVID-19 R0 estimates assuming known SI with SEAIR data (week on x-axis) for the WP (top row), seqB (second), ID (third), and IDEA (bottom) methods for known SI of 4.2 (left column), 5.2 (middle, true value), and 7.5 (right) days. The true R0 is given by the red dashed horizontal line. The inflection point related to the epidemic data curve of the SEAIR model is indicated by the blue dashed vertical lines.

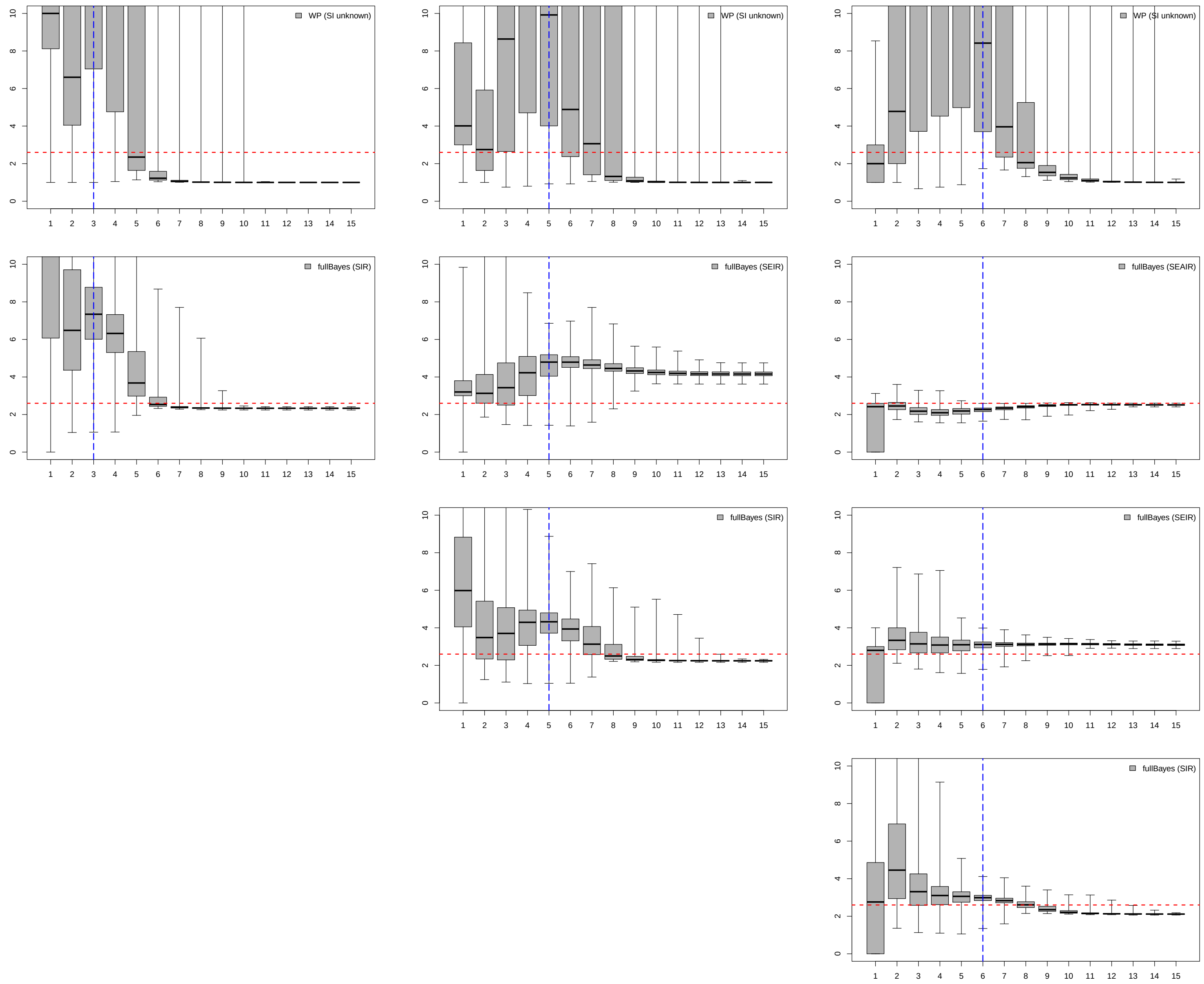

SIR data | SEIR data | SEAIR data

Figure 26: COVID-19 R0 estimates assuming unknown SI for the WP and fullBayes methods (week on x-axis). The WP method is applied to SIR, SEIR, and SEAIR data. The fullBayes method assumes SIR, SEIR and SEAIR structures (rows), and is applied to SIR, SEIR, and SEAIR data (columns) with increasing dimension. The true R0 is given by the red dashed horizontal line. The inflection points related to the epidemic data curves of the SIR, SEIR, and SEAIR models are indicated by the blue dashed vertical lines.

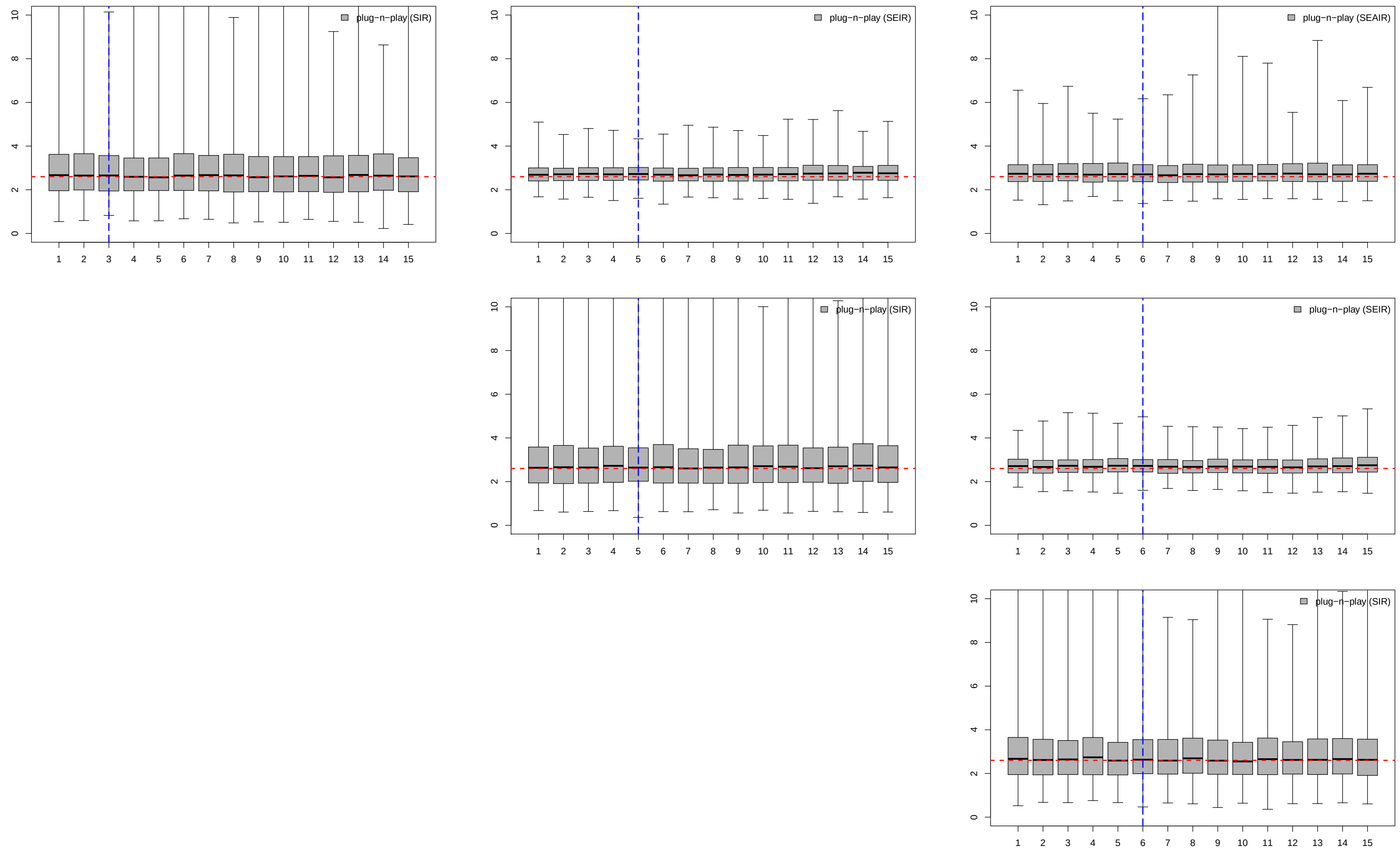

SIR data SEIR data SEAIR data

Figure 27: COVID-19 R0 estimates assuming unknown SI for the plug-n-play method (week on x-axis), assuming SIR, SEIR and SEAIR structures (rows), applied to SIR, SEIR, and SEAIR data (columns) with increasing dimension. The true R0 is given by the red dashed horizontal line. The inflection points related to the epidemic data curves of the SIR, SEIR, and SEAIR models are indicated by the blue dashed vertical lines.

4.3 Tables - COVID-19 Example

Table 14: COVID-19 example: WP, seqB, ID, and IDEA methods applied to SIR data with known SI of 4.2, 5.2 (true value), and 7.5 days. The true R_0 value is 2.6. Each row reports the median value and standard deviation (in brackets). NA count (if applicable) is the number of simulations that yield NA as a R_0 value. * (if applicable) denotes a standard deviation greater than one million.

		Week														
	SI	**1**	**2**	**3**	4	5	6	7	8	9	10	11	12	13	14	15
WP	4.2 days	12.3 (15.5)	7.1 (4.8)	8.1 (2.9)	6.3 (2.1)	2.7 (2.1)	1.4 (1.1)	1.1 (0.6)	1.0 (0.2)	1.0 (0.1)	1.0 (0.0)	1.0 (0.0)	1.0 (0.0)	1.0 (0.0)	1.0 (0.0)	1.0 (0.0)
	5.2 days	13.5 (17.0)	7.7 (5.2)	8.8 (3.1)	6.8 (2.3)	2.9 (2.3)	1.5 (1.2)	1.1 (0.6)	1.1 (0.2)	1.0 (0.1)	1.0 (0.0)	1.0 (0.0)	1.0 (0.0)	1.0 (0.0)	1.0 (0.0)	1.0 (0.0)
	7.5 days	16.5 (20.7)	9.2 (6.3)	10.5 (3.8)	8.1 (2.7)	3.5 (2.7)	1.7 (1.5)	1.3 (0.7)	1.1 (0.3)	1.0 (0.1)	1.0 (0.0)	1.0 (0.0)	1.0 (0.0)	1.0 (0.0)	1.0 (0.0)	1.0 (0.0)
seqB	NA count	0	0	0	40	481	875	987	997	1000	1000	1000	1000	1000	1000	1000
	4.2 days	2.4 (0.5)	2.1 (0.4)	2.1 (0.2)	2 (0.2)	1.8 (0.2)	1.7 (0.2)	1.9 (0.2)	1.7 (0.2)	NA	NA	NA	NA	NA	NA	NA
	5.2 days	2.7 (0.7)	2.3 (0.5)	2.4 (0.3)	2.2 (0.2)	2 (0.3)	1.9 (0.2)	2.2 (0.3)	1.9 (0.2)	NA	NA	NA	NA	NA	NA	NA
	7.5 days	3.4 (0.9)	2.9 (0.7)	3.0 (0.4)	2.8 (0.4)	2.4 (0.4)	2.3 (0.3)	2.7 (0.4)	2.2 (0.3)	NA	NA	NA	NA	NA	NA	NA
ID	4.2 days	1.4 (0.2)	1.6 (0.3)	1.8 (0.3)	1.9 (0.3)	2 (0.3)	2 (0.2)	1.9 (0.2)	1.9 (0.1)	1.8 (0.1)	1.7 (0.1)	1.7 (0.2)	1.6 (0.5)	1.5 (0.7)	0.0 (0.7)	0.0 (0.5)
	5.2 days	1.5 (0.2)	1.9 (0.4)	2.1 (0.4)	2.3 (0.4)	2.4 (0.4)	2.3 (0.3)	2.3 (0.2)	2.2 (0.2)	2.1 (0.1)	2.0 (0.1)	1.9 (0.2)	1.8 (0.6)	1.6 (0.8)	0.0 (0.8)	0.0 (0.6)
	7.5 days	1.9 (0.4)	2.4 (0.7)	2.9 (0.9)	3.3 (0.9)	3.4 (0.8)	3.4 (0.6)	3.2 (0.5)	3 (0.4)	2.8 (0.3)	2.6 (0.2)	2.5 (0.3)	2.3 (0.7)	2.1 (1)	0.0 (1)	0.0 (0.7)
IDEA	NA count	0	0	0	0	0	0	0	0	0	0	15	111	319	574	831
	4.2 days	0.7 (0.1)	0.9 (0.1)	1 (0.1)	1 (0.1)	1.1 (0.1)	1.1 (0.2)	1.2 (0.2)	1.2 (0.3)	1.3 (0.3)	1.4 (0.3)	1.5 (0.4)	1.6 (0.4)	1.6 (0.3)	1.6 (0.3)	1.6 (0.3)
	5.2 days	0.4 (0.2)	0.7 (0.1)	0.9 (0.1)	1.1 (0.2)	1.3 (0.3)	1.6 (0.4)	1.8 (0.5)	2.1 (0.6)	2.3 (0.6)	2.5 (0.6)	2.6 (0.6)	2.7 (0.6)	2.6 (0.5)	2.6 (0.5)	2.6 (0.5)
	7.5 days	0.5 (0.2)	0.8 (0.2)	0.9 (0.2)	1.1 (0.2)	1.1 (0.3)	1.2 (0.4)	1.3 (0.5)	1.5 (0.6)	1.6 (0.8)	1.9 (0.9)	2.1 (0.9)	2.3 (1)	2.4 (0.9)	2.4 (0.9)	2.4 (0.9)

Table 15: COVID-19 example: WP, seqB, ID, and IDEA methods applied to SEIR data with known SI of 4.2, 5.2 (true value), and 7.5 days. The true R_0 value is 2.6. Each row reports the median value and standard deviation (in brackets). NA count (if applicable) is the number of simulations that yield NA as a R_0 value. * (if applicable) denotes a standard deviation greater than one million.

		Week														
	SI	**1**	**2**	**3**	**4**	**5**	6	7	8	9	10	11	12	13	14	15
WP	4.2 days	4.9 (5.5)	3 (2.5)	3.7 (2)	4.7 (1.6)	4.5 (1.2)	3.9 (1.3)	2.5 (1.4)	1.5 (1.1)	1.2 (0.7)	1 (0.5)	1 (0.2)	1.0 (0.1)	1.0 (0.0)	1.0 (0.0)	1.0 (0.0)
	5.2 days	5.4 (6.0)	3.2 (2.7)	3.9 (2.2)	5 (1.7)	4.9 (1.3)	4.2 (1.4)	2.6 (1.5)	1.6 (1.2)	1.2 (0.8)	1.1 (0.5)	1.0 (0.3)	1.0 (0.1)	1.0 (0.0)	1.0 (0.0)	1.0 (0.0)
	7.5 days	6.6 (7.3)	3.9 (3.2)	4.6 (2.6)	5.9 (2.1)	5.7 (1.6)	4.9 (1.7)	3.1 (1.8)	1.8 (1.4)	1.3 (0.9)	1.1 (0.6)	1.1 (0.3)	1.0 (0.1)	1.0 (0.1)	1.0 (0.0)	1.0 (0.0)
	NA count	0	0	0	0	0	27	211	464	643	758	779	788	792	792	792
seqB	4.2 days	1.8 (0.5)	1.5 (0.4)	1.7 (0.3)	1.8 (0.3)	1.8 (0.2)	1.7 (0.2)	1.6 (0.2)	1.4 (0.2)	1.3 (0.2)	1.2 (0.1)	1.2 (0.1)	1.2 (0.0)	1.2 (0.0)	1.2 (0.0)	1.2 (0.0)
	5.2 days	1.9 (0.6)	1.7 (0.5)	1.9 (0.4)	2.0 (0.3)	2.0 (0.2)	1.9 (0.3)	1.7 (0.3)	1.5 (0.3)	1.3 (0.2)	1.3 (0.2)	1.3 (0.1)	1.3 (0.0)	1.3 (0.0)	1.3 (0.0)	1.3 (0.0)
	7.5 days	2.3 (0.8)	1.9 (0.7)	2.2 (0.6)	2.5 (0.5)	2.5 (0.3)	2.3 (0.4)	2 (0.4)	1.7 (0.4)	1.5 (0.3)	1.4 (0.2)	1.4 (0.1)	1.4 (0.1)	1.4 (0.0)	1.4 (0.0)	1.4 (0.0)
ID	4.2 days	1.2 (0.1)	1.3 (0.2)	1.4 (0.2)	1.5 (0.3)	1.5 (0.3)	1.6 (0.3)	1.6 (0.3)	1.6 (0.2)	1.6 (0.2)	1.6 (0.2)	1.5 (0.2)	1.5 (0.3)	1.4 (0.4)	1.4 (0.6)	1.3 (0.7)
	5.2 days	1.3 (0.2)	1.4 (0.3)	1.5 (0.3)	1.6 (0.4)	1.7 (0.4)	1.8 (0.4)	1.8 (0.3)	1.8 (0.3)	1.8 (0.3)	1.7 (0.2)	1.7 (0.2)	1.7 (0.3)	1.6 (0.5)	1.5 (0.6)	1.4 (0.7)
	7.5 days	1.4 (0.3)	1.6 (0.5)	1.8 (0.6)	2.0 (0.7)	2.1 (0.7)	2.3 (0.7)	2.3 (0.6)	2.3 (0.5)	2.3 (0.4)	2.2 (0.4)	2.2 (0.3)	2.1 (0.4)	1.9 (0.6)	1.8 (0.8)	1.7 (0.9)
	NA count	0	0	3	9	10	11	11	11	11	11	12	27	90	211	390
IDEA	4.2 days	0.7 (0.1)	0.9 (0.1)	1 (0.1)	1.1 (0.1)	1.1 (0.1)	1.2 (0.1)	1.2 (0.1)	1.2 (0.1)	1.3 (0.1)	1.3 (0.1)	1.3 (0.1)	1.3 (0.1)	1.4 (0.1)	1.4 (0.1)	1.4 (0.1)
	5.2 days	0.6 (0.2)	0.8 (0.1)	1 (0.1)	1 (0.1)	1.1 (0.2)	1.1 (0.2)	1.2 (0.3)	1.3 (0.4)	1.4 (0.4)	1.5 (0.5)	1.7 (0.5)	1.8 (0.5)	1.8 (0.5)	1.8 (0.5)	1.8 (0.4)
	7.5 days	0.7 (0.1)	0.9 (0.1)	1 (0.1)	1.1 (0.1)	1.1 (0.1)	1.2 (0.1)	1.2 (0.1)	1.2 (0.1)	1.3 (0.1)	1.3 (0.1)	1.3 (0.1)	1.3 (0.1)	1.4 (0.1)	1.4 (0.1)	1.4 (0.1)

Table 16: COVID-19 example: WP, seqB, ID, and IDEA methods applied to SEAIR data with known SI of 4.2, 5.2 (true value), and 7.5 days. The true R_0 value is 2.6. Each row reports the median value and standard deviation (in brackets). NA count (if applicable) is the number of simulations that yield NA as a R_0 value. * (if applicable) denotes a standard deviation greater than one million.

		Week														
	SI	**1**	**2**	**3**	**4**	**5**	**6**	7	8	9	10	11	12	13	14	15
WP	4.2 days	2.5 (1.7)	3.1 (2)	3.3 (1.3)	3.4 (0.9)	3.4 (0.6)	3.2 (0.4)	2.9 (0.4)	2.3 (0.5)	1.7 (0.5)	1.3 (0.4)	1.2 (0.3)	1.1 (0.2)	1.0 (0.1)	1.0 (0.0)	1.0 (0.0)
	5.2 days	2.7 (1.9)	3.3 (2.1)	3.6 (1.4)	3.6 (1.0)	3.6 (0.6)	3.4 (0.4)	3.1 (0.4)	2.5 (0.5)	1.8 (0.5)	1.4 (0.4)	1.2 (0.3)	1.1 (0.2)	1.0 (0.1)	1.0 (0.1)	1.0 (0.0)
	7.5 days	3.3 (2.3)	3.9 (2.5)	4.2 (1.6)	4.2 (1.2)	4.2 (0.8)	4 (0.5)	3.6 (0.5)	2.9 (0.6)	2.1 (0.6)	1.6 (0.5)	1.3 (0.4)	1.1 (0.2)	1.1 (0.1)	1.0 (0.1)	1.0 (0.0)
seqB	4.2 days	1.4 (0.4)	1.7 (0.4)	1.8 (0.3)	1.8 (0.2)	1.8 (0.1)	1.8 (0.1)	1.7 (0.1)	1.5 (0.1)	1.3 (0.2)	1.2 (0.1)	1.1 (0.1)	1.1 (0.1)	1.0 (0.0)	1.0 (0.0)	1.0 (0.0)
	5.2 days	1.3 (0.3)	1.6 (0.3)	1.6 (0.2)	1.6 (0.2)	1.6 (0.1)	1.6 (0.1)	1.6 (0.1)	1.4 (0.1)	1.3 (0.1)	1.1 (0.1)	1.1 (0.1)	1.0 (0.1)	1.0 (0.0)	1.0 (0.0)	1.0 (0.0)
	7.5 days	1.6 (0.5)	2.0 (0.6)	2.1 (0.4)	2.2 (0.3)	2.1 (0.2)	2.1 (0.1)	2 (0.1)	1.8 (0.2)	1.5 (0.2)	1.2 (0.2)	1.1 (0.2)	1.1 (0.1)	1.1 (0.1)	1.1 (0.0)	1.1 (0.0)
ID	4.2 days	1.1 (0.1)	1.2 (0.1)	1.3 (0.1)	1.3 (0.2)	1.4 (0.2)	1.4 (0.2)	1.5 (0.2)	1.5 (0.2)	1.5 (0.2)	1.5 (0.1)	1.5 (0.1)	1.5 (0.1)	1.5 (0.1)	1.4 (0.1)	1.4 (0.1)
	5.2 days	1.1 (0.1)	1.3 (0.1)	1.4 (0.2)	1.4 (0.2)	1.5 (0.2)	1.6 (0.2)	1.6 (0.2)	1.6 (0.2)	1.6 (0.2)	1.6 (0.2)	1.6 (0.2)	1.6 (0.2)	1.6 (0.1)	1.6 (0.1)	1.5 (0.1)
	7.5 days	1.2 (0.2)	1.4 (0.2)	1.6 (0.3)	1.7 (0.3)	1.8 (0.3)	1.9 (0.3)	2.0 (0.3)	2.0 (0.3)	2.1 (0.3)	2.1 (0.3)	2.0 (0.3)	2.0 (0.2)	1.9 (0.2)	1.9 (0.2)	1.8 (0.2)
	NA count	0	0	2	5	6	6	6	6	6	6	6	6	6	6	7
IDEA	4.2 days	0.8 (0.1)	0.8 (0.1)	0.9 (0.1)	1 (0.1)	1 (0.1)	1 (0.1)	1.1 (0.1)	1.1 (0.1)	1.2 (0.1)	1.2 (0.1)	1.3 (0.2)	1.3 (0.2)	1.4 (0.2)	1.4 (0.2)	1.5 (0.2)
	5.2 days	0.8 (0.2)	0.8 (0.1)	0.9 (0.1)	0.9 (0.1)	1.0 (0.1)	1.0 (0.1)	1.1 (0.1)	1.1 (0.2)	1.2 (0.2)	1.3 (0.2)	1.4 (0.2)	1.4 (0.2)	1.5 (0.2)	1.6 (0.2)	1.6 (0.2)
	7.5 days	0.7 (0.2)	0.7 (0.1)	0.8 (0.1)	0.9 (0.1)	1 (0.2)	1.1 (0.2)	1.1 (0.2)	1.2 (0.2)	1.3 (0.3)	1.4 (0.3)	1.5 (0.4)	1.7 (0.4)	1.8 (0.4)	1.9 (0.4)	2 (0.4)

Table 17: COVID-19 example: WP, fullBayes, and plug-n-play methods applied to SIR data, with SI unknown. The true R_0 value is 2.6. fullBayes and plug-n-play methods assume SIR, SEIR, and SEAIR model structures. Each row reports the median value and standard deviation (in brackets). NA count (if applicable) is the number of simulations that yield NA as a R_0 value. * (if applicable) denotes a standard deviation greater than one million.

		Week														
	data	1	2	3	4	5	6	7	8	9	10	11	12	13	14	15
WP	SIR	10.0 (*)	6.6 (*)	93.4 (*)	32.8 (*)	2.3 (*)	1.2 (*)	1.1 (*)	1.0 (*)	1.0 (1979.3)	1.0 (265.7)	1.0 (0.0)	1.0 (0.0)	1.0 (0.0)	1.0 (0.0)	1.0 (0.0)
	SEIR	4.0 (190.8)	2.8 (3643.9)	8.6 (*)	25.2 (*)	9.9 (*)	4.9 (*)	3.1 (*)	1.3 (*)	1.1 (*)	1.0 (*)	1.0 (*)	1.0 (*)	1.0 (2351.8)	1.0 (0.0)	1.0 (0.0)
	SEAIR	2.0 (1.9)	4.8 (20601.2)	13.8 (22345.2)	11.1 (827170.8)	11.8 (142423.5)	8.4 (146802.3)	4 (885706.2)	2.1 (*)	1.5 (*)	1.2 (*)	1.1 (*)	1.0 (*)	1.0 (*)	1.0 (1198.3)	1.0 (0.0)
plug-n-play (SIR)	SIR	2.7 (1.4)	2.7 (1.4)	2.7 (1.3)	2.6 (1.4)	2.6 (1.4)	2.7 (1.5)	2.7 (1.6)	2.7 (1.4)	2.6 (1.5)	2.6 (1.4)	2.6 (1.4)	2.6 (1.4)	2.7 (1.4)	2.6 (1.4)	2.6 (1.5)
	SEIR	2.6 (1.5)	2.7 (1.4)	2.6 (1.5)	2.7 (1.4)	2.6 (1.4)	2.7 (1.4)	2.6 (1.4)	2.6 (1.4)	2.7 (1.4)	2.7 (1.3)	2.7 (1.5)	2.6 (1.5)	2.7 (1.3)	2.7 (1.5)	2.6 (1.4)
	SEAIR	2.7 (1.4)	2.6 (1.4)	2.6 (1.3)	2.7 (1.5)	2.6 (1.3)	2.6 (1.4)	2.6 (1.3)	2.7 (1.3)	2.6 (1.4)	2.6 (1.4)	2.7 (1.3)	2.6 (1.3)	2.6 (1.4)	2.7 (1.3)	2.6 (1.4)
plug-n-play (SEIR)	SEIR	2.7 (0.5)	2.7 (0.4)	2.7 (0.5)	2.7 (0.5)	2.7 (0.4)	2.7 (0.5)	2.7 (0.5)	2.7 (0.5)	2.7 (0.5)	2.7 (0.5)	2.7 (0.5)	2.7 (0.5)	2.7 (0.5)	2.8 (0.5)	2.8 (0.5)
	SEAIR	2.7 (0.5)	2.7 (0.4)	2.7 (0.5)	2.7 (0.4)	2.7 (0.5)	2.7 (0.5)	2.7 (0.5)	2.7 (0.4)	2.7 (0.5)	2.7 (0.4)	2.7 (0.5)	2.7 (0.5)	2.7 (0.5)	2.7 (0.5)	2.7 (0.5)
plug-n-play (SEAIR)	SEAIR	1.8 0.6)	1.8 (0.6)	1.8 (0.6)	1.8 (0.6)	1.8 (0.6)	1.8 (0.6)	1.8 (0.6)	1.8 (0.6)	1.8 (0.6)	1.8 (0.6)	1.8 (0.6)	1.8 (0.6)	1.8 (0.7)	1.8 (0.7)	1.8 (0.6
fullBayes (SIR)	SIR	10.7 (12.8)	6.5 (5)	7.3 (2.6)	6.3 (1.6)	3.7 (1.7)	2.5 (0.9)	2.4 (0.4)	2.3 (0.2)	2.3 (0.0)	2.3 (0.0)	2.3 (0.0)	2.3 (0.0)	2.3 (0.0)	2.3 (0.0)	2.3 (0.0)
	SEIR	6.0 (4.8)	3.5 (2.7)	3.7 (1.8)	4.3 (1.3)	4.3 (1)	3.9 (0.9)	3.1 (0.9)	2.5 (0.7)	2.3 (0.4)	2.3 (0.3)	2.3 (0.2)	2.2 (0.1)	2.2 (0.0)	2.2 (0.0)	2.2 (0.0)
	SEAIR	2.8 (2.4)	4.5 (3.3)	3.3 (1.4)	3.1 (0.8)	3.1 (0.5)	3 (0.3)	2.8 (0.2)	2.6 (0.2)	2.4 (0.2)	2.2 (0.2)	2.1 (0.1)	2.1 (0.1)	2.1 (0.0)	2.1 (0.0)	2.1 (0.0)
fullBayes (SEIR)	SEIR	3.2 (1.3)	3.1 (1.4)	3.4 (1.4)	4.2 (1.3)	4.8 (1)	4.8 (0.7)	4.6 (0.5)	4.5 (0.4)	4.3 (0.3)	4.2 (0.2)	4.2 (0.2)	4.2 (0.2)	4.2 (0.2)	4.2 (0.2)	4.2 (0.2)
	SEAIR	2.8 (1.4)	3.3 (0.9)	3.1 (0.8)	3.1 (0.6)	3.1 (0.4)	3.1 (0.3)	3.1 (0.2)	3.1 (0.1)	3.1 (0.1)	3.1 (0.1)	3.1 (0.1)	3.1 (0.1)	3.1 (0.1)	3.1 (0.1)	3.1 (0.1)
fullBayes (SEAIR)	SEAIR	2.4 (1.2)	2.5 (0.3)	2.2 (0.3)	2.1 (0.2)	2.2 (0.2)	2.3 (0.2)	2.3 (0.1)	2.4 (0.1)	2.5 (0.1)	2.5 (0.1)	2.5 (0.0)	2.5 (0.0)	2.5 (0.0)	2.5 (0.0)	2.5 (0.0)

4.4 Real World COVID-19 Example

Community transmission started in week 5 of the pandemic (13). Lockdowns were inititated as early as week 7 (13). We present results using data from weeks 5 to 10 of the pandemic, for the provinces of BC, Ontario and Quebec, and also for the entire Canadian population. Estimates of the serial distribution (SI) from the WP, fullBayes and plug-n-play methods for each region of study are also provided.

Table 18: Covid19 example results BC; lockdown mid week 7

		Week									
	SI	1	2	3	4	5	6	7	8	9	10
WP	2					0.00	14.42	3.91	6.01	2.35	1.51
	5					0.00	18.57	4.96	7.32	2.91	1.77
	8					0.00	23.99	6.33	9.07	3.65	2.13
	unknown					0.00	16.55	3.80	403.87	12.17	1.48
seqB	2					NA	1.74	1.38	1.51	1.24	1.11
	5					NA	2.86	1.95	2.26	1.59	1.28
	8					NA	3.97	2.52	3.02	1.95	1.45
ID	2					1.00	1.21	1.28	1.33	1.35	1.34
	5					1.00	1.60	1.85	2.04	2.10	2.09
	8					1.00	2.13	2.67	3.12	3.29	3.25
IDEA	2					NA	0.69	0.89	0.99	1.08	1.17
	5					NA	0.39	0.75	0.97	1.22	1.48
	8					NA	0.22	0.64	0.95	1.38	1.88
	model										
plug-n-play	SIR					2.11	1.93	2.60	6.70	2.60	2.03
	SEIR					2.18	2.36	2.61	2.17	2.88	2.21
	SEAIR					2.23	2.48	3.39	1.89	3.20	2.92
fullBayes	SIR					0.00	4.08	3.27	5.59	2.28	1.48
	SEIR					0.00	5.40	3.68	5.71	2.31	1.49
	SEAIR					0.00	2.81	2.12	3.20	1.85	1.36

Table 19: Covid19 example SI estimation results BC; lockdown mid week 7

		Week									
		1	2	3	4	5	6	7	8	9	10
WP	SI unknown					NA	54.66	0.50	17.93	1.78	0.50
plug-n-play	SIR					0.64	0.93	0.76	0.89	0.82	0.68
	SEIR					0.73	0.51	0.68	0.70	0.86	0.70
	SEAIR					0.73	0.89	0.68	0.73	0.74	0.88
fullBayes	SIR					NA	2.20	2.17	3.31	1.66	1.24
	SEIR					NA	1.29	2.57	3.98	2.63	2.76
	SEAIR					NA	1.01	0.90	1.75	1.44	1.28

Table 20: Covid19 example R0 results ON; lockdown mid week 8

		Week									
	SI	1	2	3	4	5	6	7	8	9	10
WP	2					0.00	4.12	2.91	4.16	3.19	3.79
	5					0.00	5.30	3.59	4.98	3.89	4.55
	8					0.00	6.85	4.49	6.08	4.80	5.55
	unknown					0.00	4.00	2.84	108.44	8.47	9.98
seqB	2					NA	1.39	1.30	1.40	1.33	1.38
	5					NA	1.97	1.74	2.00	1.81	1.94
	8					NA	2.56	2.18	2.60	2.30	2.50
ID	2					1.58	1.56	1.52	1.51	1.50	1.49
	5					3.16	3.03	2.86	2.81	2.75	2.72
	8					6.29	5.90	5.38	5.22	5.06	4.96
IDEA	2					NA	1.64	1.66	1.61	1.60	1.58
	5					NA	3.42	3.54	3.31	3.23	3.14
	8					NA	7.15	7.57	6.77	6.52	6.23
	model										
plug-n-play	SIR					2.10	1.55	3.96	2.19	4.92	2.90
	SEIR					2.18	3.26	2.37	3.80	3.54	2.49
	SEAIR					2.23	3.29	2.31	2.55	2.95	2.34
fullBayes	SIR					3.07	3.48	2.76	3.99	3.11	3.71
	SEIR					3.40	4.11	3.03	4.09	3.14	3.72
	SEAIR					2.52	2.46	1.93	2.57	2.23	2.56

Table 21: Covid19 example SI estimation results ON; lockdown mid week 8

		Week									
		1	2	3	4	5	6	7	8	9	10
WP	SI unknown					NA	6.46	0.50	14.93	1.46	1.56
plug-n-play	SIR					0.64	0.55	0.82	0.78	0.96	0.65
	SEIR					0.73	0.77	1.03	0.74	0.69	0.69
	SEAIR					0.73	0.75	0.79	0.85	0.65	0.78
fullBayes	SIR					0.95	1.63	1.86	2.58	2.10	2.35
	SEIR					1.22	2.08	2.60	3.22	3.00	3.25
	SEAIR					0.82	0.96	0.88	1.54	1.57	1.82

Table 22: Covid19 example results QC; lockdown mid week 8

		Week									
	SI	1	2	3	4	5	6	7	8	9	10
WP	2					0.00	2.06	5.44	8.32	14.92	3.10
	5					0.00	2.65	6.54	10.28	18.68	3.93
	8					0.00	3.43	8.02	12.83	23.59	5.02
	unknown					0.00	2.00	3510.00	1.05e+06	9.24e+11	10300.00
seqB	2					NA	1.12	1.47	1.60	1.76	NA
	5					NA	1.35	2.17	2.49	2.91	NA
	8					NA	1.63	2.88	3.39	4.06	NA
ID	2					1.00	1.05	1.12	1.19	1.25	1.29
	5					1.00	1.13	1.34	1.54	1.75	1.88
	8					1.00	1.22	1.60	2.00	2.46	2.76
IDEA	2					NA	0.91	0.87	0.89	0.90	0.95
	5					NA	0.78	0.71	0.74	0.77	0.88
	8					NA	0.67	0.58	0.61	0.65	0.81
	model										
plug-n-play	SIR					2.11	1.93	2.60	6.70	2.60	2.03
	SEIR					2.18	2.36	2.61	2.17	2.88	2.21
	SEAIR					2.23	2.48	3.39	1.89	3.20	2.92
fullBayes	SIR					0.00	1.35	3.07	6.86	14.17	3.02
	SEIR					0.00	3.00	4.14	7.19	14.22	3.02
	SEAIR					0.00	2.52	2.22	3.28	6.19	2.28

References

[1] White LF, Pagano M. A likelihood-based method for real-time estimation of the serial interval and reproductive number of an epidemic. Statistics in Medicine. 2008;27:2999–3016.

Table 23: Covid19 example SI estimation results QC; lockdown mid week 8

		Week 1	2	3	4	5	6	7	8	9	10
WP	SI unknown					NA	0.50	24.49	43.90	85.87	6.99
plug-n-play	SIR					0.64	0.93	0.76	0.89	0.82	0.68
	SEIR					0.73	0.51	0.68	0.70	0.86	0.70
	SEAIR					0.73	0.89	0.68	0.73	0.74	0.88
fullBayes	SIR					NA	0.98	1.93	3.83	7.73	2.02
	SEIR					NA	1.33	1.93	4.43	8.11	2.87
	SEAIR					NA	0.82	0.86	1.43	3.30	1.83

Table 24: Covid19 example R0 results CANADA

	SI	Week 1	2	3	4	5	6	7	8	9	10
WP	2					0.00	5.59	4.15	5.80	4.51	2.79
	5					0.00	7.20	5.17	7.08	5.58	3.42
	8					0.00	9.30	6.51	8.78	6.97	4.24
	unknown					0.00	5.43	4.04	507.64	18.36	2.73
seqB	2					NA	1.48	1.40	1.50	1.42	1.29
	5					NA	2.20	2.00	2.24	2.06	1.72
	8					NA	2.92	2.59	2.98	2.69	2.15
ID	2					1.74	1.71	1.68	1.66	1.65	1.63
	5					4.01	3.84	3.64	3.57	3.50	3.39
	8					9.24	8.60	7.89	7.64	7.41	7.06
IDEA	2					NA	1.81	1.82	1.77	1.76	1.76
	5					NA	4.40	4.47	4.19	4.09	4.12
	8					NA	10.69	10.98	9.91	9.51	9.62
	model										
plug-n-play	SIR					2.10	1.55	3.57	1.55	6.09	1.45
	SEIR					2.18	3.26	2.37	3.80	3.31	2.67
	SEAIR					2.23	3.29	2.31	2.55	2.95	2.34
fullBayes	SIR					3.67	4.62	3.95	5.61	4.39	2.73
	SEIR					3.80	5.18	4.10	5.65	4.40	2.73
	SEAIR					2.49	2.70	2.39	3.24	2.91	2.13

Table 25: Covid19 example SI estimation results CANADA

		Week 1	2	3	4	5	6	7	8	9	10
WP	SI unknown					NA	0.50	0.50	18.92	1.64	0.50
plug-n-play	SIR					0.64	0.55	0.75	0.66	0.87	0.73
	SEIR					0.73	0.77	1.03	0.74	0.86	0.68
	SEAIR					0.73	0.75	0.79	0.85	0.65	0.78
fullBayes	SIR					1.05	2.34	2.44	3.32	2.70	1.88
	SEIR					1.35	2.73	3.13	4.03	3.50	2.84
	SEAIR					0.87	1.11	1.15	1.91	2.00	1.81

[2] Bettencourt LMA, Riberio RM. Real time Bayesian estimation of the epidemic potential of emerging infectious diseases. PLOS ONE. 2008;3.

[3] Fisman DN, Hauck TS, Tuite AR, Greer AL. An IDEA for Short Term Outbreak Projection: Nearcasting Using the Basic Reproduction Number. PLOS ONE. 2013;8.

[4] King AA, Ionides EL, Breto C. Statistical Inference for Partially Observed Markov Processes; 2017. R package version 1.12.

[5] O'Neill PD, Roberts GO. Bayesian inference for partially observed stochastic epidemics. Journal of the Royal Statistical Society Series A. 1999;162:121–129.

[6] Anderson RM, May RM. Infectious diseases of humans: dynamics and control. Oxford university press; 1992.

[7] Allen LJ, Brauer F, Van den Driessche P, Wu J. Mathematical epidemiology. vol. 1945. Springer; 2008.

[8] Keeling MJ, Rohani P. Modeling infectious diseases in humans and animals. Princeton university press; 2011.

[9] Chowell G, Nishiura H, Bettencourt LM. Comparative estimation of the reproduction number for pandemic influenza from daily case notification data. Journal of the Royal Society Interface. 2007;4(12):155–166.

[10] Biggerstaff M, Cauchemez S, Reed C, Gambhir M, Finelli L. Estimates of the reproduction number for seasonal, pandemic, and zoonotic influenza: a systematic review of the literature. BMC Infectious Diseases. 2014;14(1):1–20.

[11] Knight J, Mishra S. Estimating effective reproduction number using generation time versus serial interval, with application to COVID-19 in the Greater Toronto Area, Canada. Infectious Disease Modelling. 2020;5:889–896.

[12] Alene M, Yismaw L, Assemie MA, Ketema DB, Gietaneh W, Birhan TY. Serial interval and incubation period of COVID-19: a systematic review and meta-analysis. BMC Infectious Diseases. 2021;21(1):1–9.

[13] Akanteva A, Dick D, Heffernan JM. A database of public healthcare mitigation and relaxation during the COVID-19 pandemic, for all Canadian provinces; 2022. Database access - available on request.
