# Supplementary material for: Estimating the basic reproduction number at the beginning of an outbreak
Source: PLoS One. 2022 Jun 17;17(6):e0269306. doi: 10.1371/journal.pone.0269306 (PMC9205483; doi:10.1371/journal.pone.0269306)
Supplement: S1 Appendix — (PDF) [file pone.0269306.s002.pdf]

Appendix

Disease models

We employ SIR, SEIR and SEAIR model structures, where S, E, A, I and R denotes susceptible, exposed (infected, no symptoms, not infectious), asymptomatic infected (infected, no symptoms, infectious), symptomatic infected (infected, symptomatic, infectious), and recovered individuals, respectively. A flow diagram of each model is shown in Figure 7. The ODEs governing our models are given below.

SIR model

$$
\begin{aligned}
\frac{dS(t)}{dt} &= -\beta S(t)\frac{I(t)}{N} \\
\frac{dI(t)}{dt} &= \beta S(t)\frac{I(t)}{N} - \gamma I(t) \\
\frac{dR(t)}{dt} &= \gamma I(t) \\
N &= S(t) + I(t) + R(t)
\end{aligned}
$$

SEIR model

$$
\begin{aligned}
\frac{dS(t)}{dt} &= -\beta S(t)\frac{I(t)}{N} \\
\frac{dE(t)}{dt} &= \beta S(t)\frac{I(t)}{N} - \alpha E(t) \\
\frac{dI(t)}{dt} &= \alpha E(t) - \gamma I(t) \\
\frac{dR(t)}{dt} &= \gamma I(t) \\
N &= S(t) + E(t) + I(t) + R(t)
\end{aligned}
$$

SEAIR model

$$\begin{aligned}
\frac{dS(t)}{dt} &= -\beta S(t)\frac{I(t)+A(t)}{N} \\
\frac{dE(t)}{dt} &= \beta S(t)\frac{I(t)+A(t)}{N} - \alpha E(t) \\
\frac{dA(t)}{dt} &= \alpha E(t) - \rho A(t) \\
\frac{dI(t)}{dt} &= \rho A(t) - \gamma I(t) \\
\frac{dR(t)}{dt} &= \gamma I(t) \\
N &= S(t) + E(t) + A(t) + I(t) + R(t)
\end{aligned}$$

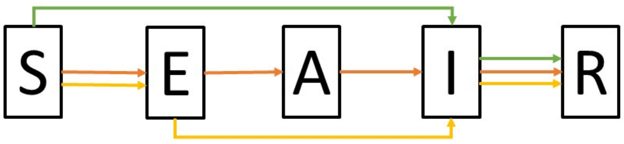

Fig 7. Flow diagrams of the SIR (green arrows), SEIR (yellow arrows), and SEAIR (orange arrows) models.

Least squares estimation for the IDEA method

From (2), our objective function is

$$Q = \sum_{j=1}^{k} \left(\log R_0 - \frac{1}{s_j} \log \widetilde{I}(s_j) - s_j \log(1+d) \right)^2 .$$

Let $\eta = \log R_0$ and $\xi = \log(1+d)$ and note that both of these transfromations are monotone increasing. Next, we take partial derivatives of $Q = Q(\eta, \xi)$ with respect to η and ξ.

$$\frac{\partial Q}{\partial \eta} = 2 \sum_{j=1}^{k} \left(\eta - \frac{1}{s_j} \log \widetilde{I}(s_j) - s_j \xi \right)$$

$$\frac{\partial Q}{\partial \xi} = 2 \sum_{j=1}^{k} s_j \left(\eta - \frac{1}{s_j} \log \widetilde{I}(s_j) - s_j \xi \right)$$

Finally, we minimize Q by setting $\frac{\partial Q}{\partial \eta} = 0$ and $\frac{\partial Q}{\partial \xi} = 0$. Solving these equations, we obtain

$$\xi = \frac{k\eta - \sum_{j=1}^{k} \frac{1}{s_j} \log \widetilde{I}(s_j)}{\sum_{j=1}^{k} s_j},$$

$$\xi = \frac{\eta \sum_{j=1}^{k} s_j - \sum_{j=1}^{k} \log \widetilde{I}(s_j)}{\sum_{j=1}^{k} s_j^2}.$$

In the last step, we solve for $\eta = \log R_0$ and thus find

$$\hat{R}_{IDEA} = \exp \left(\frac{\sum_{j=1}^{k} s_j^2 \sum_{j=1}^{k} \frac{1}{s_j} \log \widetilde{I}(s_j) - \sum_{j=1}^{k} s_j \sum_{j=1}^{k} \log \widetilde{I}(s_j)}{k \sum_{j=1}^{k} s_j^2 - (\sum_{j=1}^{k} s_j)^2} \right).$$

Posterior distributions

$$\begin{aligned} & L(\tau, m | \theta, \tau_1^I) \\ & = \left\{ \prod_{i=2}^{m_k} \frac{\beta S(\tau_i^E)}{N} \left(I(\tau_i^E) + A(\tau_i^E) \right) \right\} \left\{ \prod_{i=2}^{m_k} \sigma E(\tau_i^A) \right\} \left\{ \prod_{i=2}^{m_k} \rho A(\tau_i^I) \right\} \left\{ \prod_{i=1}^{m_{k-1}} \gamma I(\tau_i^R) \right\} \\ & \quad \times \exp \left\{ - \int_{\tau_1^I}^{t_k} \left[\beta S(t) \left(I(t) + A(t) \right) / N + \sigma E(t) + \rho A(t) + \gamma I(t) \right] dt \right\}. \end{aligned}$$

First, we calculate the posterior distributions of each of the elements of θ. For ease of exposition, in each calculation we drop the subscript on the scale parameter k in the priors.

$$\begin{aligned} & \pi(\beta | \sigma, \rho, \gamma, \tau_1^I, \tau) \\ & \propto L(\tau, m | \theta, \tau_1^I) \pi(\beta) \\ & \propto \left\{ \prod_{i=2}^{m_k} \frac{\beta S(\tau_i^E)}{N} \left(I(\tau_i^E) + A(\tau_i^E) \right) \right\} \exp \left\{ - \int_{\tau_1^I}^{t_k} \beta S(t) \left(I(t) + A(t) \right) / N dt \right\} \beta^{\alpha - 1} \exp(-k\beta) \\ & \propto \beta^{(\alpha + m_k) - 1} \exp \left\{ -\beta \left(k + \int_{\tau_1^I}^{t_k} S(t) \left(I(t) + A(t) \right) / N dt \right) \right\}. \end{aligned}$$

Hence the posterior distribution for β is gamma with shape parameter $\alpha + m_k$ and scale parameter $k + \int_{\tau_1^I}^{t_k} S(t)\left(I(t) + A(t)\right)/N dt$.

$$\begin{aligned}
\pi(\sigma|\beta,\rho,\gamma,\tau_1^I,\tau) &\propto L(\tau,m|\theta,\tau_1^I)\pi(\sigma) \\
&\propto \left\{\prod_{i=2}^{m_k} \sigma E(\tau_i^A)\right\} \exp\left\{-\int_{\tau_1^I}^{t_k} \sigma E(t)dt\right\} \sigma^{\alpha-1}\exp(-k\sigma) \\
&\propto \sigma^{(m_k+\alpha)-1}\exp\left\{-\sigma\left(k + \int_{\tau_1^I}^{t_k} E(t)dt\right)\right\}
\end{aligned}$$

Hence the posterior distribution for σ is gamma with shape parameter $\alpha + m_k$ and scale parameter $k + \int_{\tau_1^I}^{t_k} E(t)dt$.

$$\begin{aligned}
\pi(\rho|\beta,\sigma,\gamma,\tau_1^I,\tau) &\propto L(\tau,m|\theta,\tau_1^I)\pi(\rho) \\
&\propto \left\{\prod_{i=2}^{m_k} \rho A(\tau_i^I)\right\} \exp\left\{-\int_{\tau_1^I}^{t_k} \rho A(t)dt\right\} \rho^{\alpha-1}\exp(-k\rho) \\
&= \rho^{(\alpha+m_k)-1}\exp\left\{-\rho\left(k + \int_{\tau_1^I}^{t_k} A(t)dt\right)\right\}
\end{aligned}$$

Hence the posterior distribution for σ is gamma with shape parameter $\alpha + m_k$ and scale parameter $k + \int_{\tau_1^I}^{t_k} A(t)dt$.

$$\begin{aligned}
\pi(\gamma|\beta,\rho,\gamma,\tau_1^I,\tau) &\propto L(\tau,m|\theta,\tau_1^I)\pi(\gamma) \\
&\propto \left\{\prod_{i=1}^{m_{k-1}} \gamma I(\tau_i^R)\right\} \exp\left\{-\int_{\tau_1^I}^{t_k} \gamma I(t)dt\right\} \gamma^{\alpha-1}\exp(-k\gamma) \\
&\propto \gamma^{(\alpha+m_{k-1})-1}\exp\left\{-\gamma\left(k + \int_{\tau_1^I}^{t_k} I(t)dt\right)\right\}
\end{aligned}$$

Hence the posterior distribution for γ is gamma with shape parameter $\alpha + m_k$ and scale parameter $k + \int_{\tau_1^I}^{t_k} I(t)dt$.

Lastly, we calculate the posterior distribution of $-\tau_1^I$, with a prior distribution of exponential, rate one.

$$\begin{aligned}
\pi(-\tau_1^I|\theta,\tau) &\propto L(\tau,m|\theta,\tau_1^I)\pi(-\tau_1^I) \\
&\propto \exp\left(-\int_{\tau_1^I}^{\tau_2^I} \frac{\beta}{N}S(t)(I(t)+A(t)) + \sigma E(t) + \rho A(t) + \gamma I(t)dt\right)\exp(\tau_1^I).
\end{aligned}$$

For $\tau_1^I \leq t < \tau_2^I$, we have that $S(t) = N$, $I(t) = 1$, $E(t), A(t) = 0$, and hence

$$\int_{\tau_1^I}^{\tau_2^I} \frac{\beta}{N}S(t)(I(t)+A(t)) + \sigma E(t) + \rho A(t) + \gamma I(t)dt = (\beta+\gamma)(\tau_2^I - \tau_1^I),$$

from which it follows that

$$\begin{aligned}
\pi(-\tau_1^I|\beta,\sigma,\rho,\gamma,\tau) &\propto \exp\left(-(\beta+\gamma)(\tau_2^I - \tau_1^I) + \tau_1^I\right) \\
&\propto \exp\left((\beta+\gamma+1)\tau_1^I\right),
\end{aligned}$$

and hence the posterior of $-\tau_1^I$ is exponential with rate $\beta + \gamma + 1$. Note that this formula is the same for all models.

Symmetric Proposal

We need to show that $g(\tau|\tau_l)/g(\tau_l|\tau) = 1$. To do this, we use the fact that $g(\tau|\tau_l)$ does not depend on τ_l. Moreover, $g(\tau)$ is a product of uniform distributions, so $g(\tau)$ also does not depend on τ. Therefore $g(\tau|\tau_l) = c$ for some constant c, and hence $g(\tau|\tau_l) = g(\tau_l|\tau) = 1$.

Sensitivity to Prior

As mentioned previously, the joint prior distribution of the unknown rate parameters θ is made up of independent gamma distributions given by $\Gamma(\alpha, k)$ with mean k/α. In the main text, we assume that α is the same for the parameters $\beta, \sigma, \rho, \gamma$, while k varies and if appropriate will be denoted by $k_\beta, k_\sigma, k_\rho, k_\gamma$. In the simulations we took these to be $\alpha = 1$ and $k_\beta = k_\sigma = 3, k_\rho = 2, k_\gamma = 5$. The prior distribution on $-\tau_1^I$ is exponential with rate one, and this is independent from the θ vector. In Figure 8, we compare the results in the main text with results repeating the method with a different prior distribution for the SIR/SEIR/SEAIR data assuming SIR/SEIR/SEAIR models respectively. The modified prior for the comparison is $k_\beta = 9/4, k_\gamma = 3$. These were chosen as alternative reasonable parameters for the influenza. The plots show that there was very little change between the two versions.

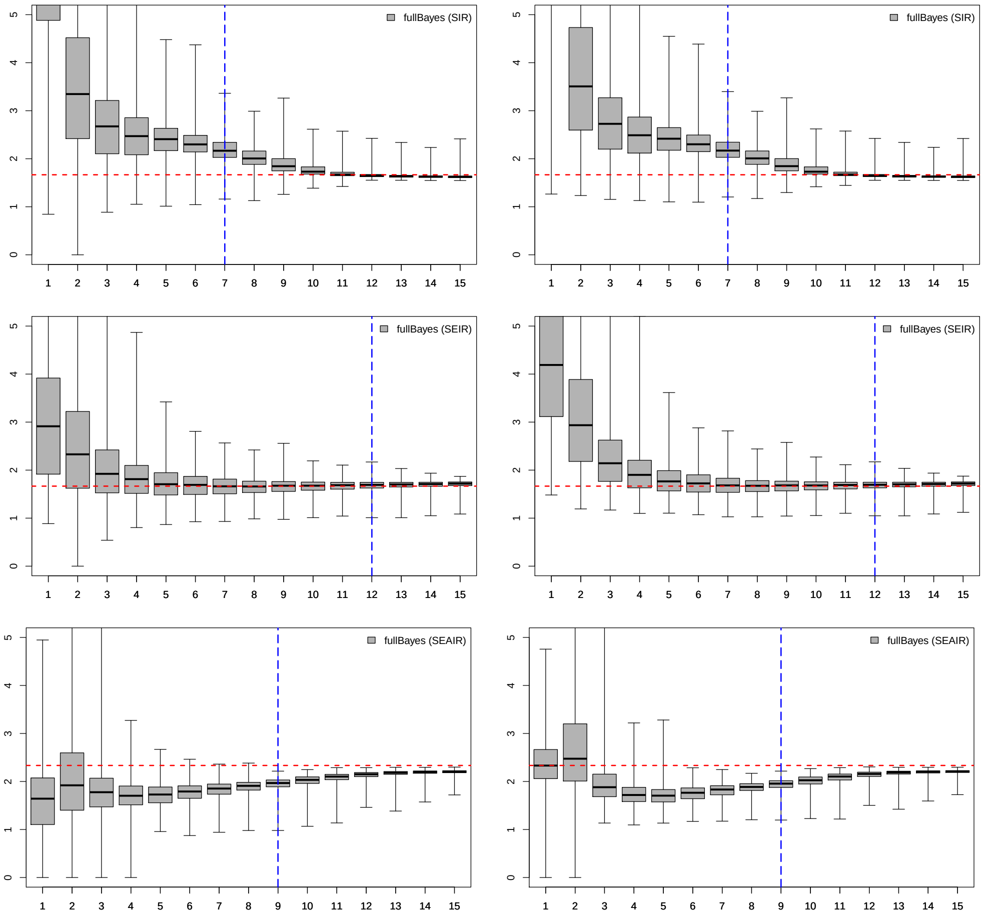

Fig 8. Comparison of the fullBayes method for SIR, SEIR, and SEAIR data with two different prior distributions: same as main text is on the right and the modified version is on the left. The inflection point for the epidemic is marked in blue, and the true R_0 for the data is marked as a horizontal red line.
