# Supplementary material for: Estimating the basic reproduction number at the beginning of an outbreak
Source: PLoS One. 2022 Jun 17;17(6):e0269306. doi: 10.1371/journal.pone.0269306 (PMC9205483; doi:10.1371/journal.pone.0269306)
Supplement: Attachment — Submitted filename: ProjectInc_revisions_replytoreviewers.pdf [file pone.0269306.s003.pdf]

Response to reviewers for PONE-D-21-22343: "Estimating the basic reproduction number at the beginning of an outbreak"

Sawitree Boonpatcharanon, Jane Heffernan, Hanna Jankowski

January 21, 2022

Dear Editor and Reviewers,

We thank the reviewers for their comments. We have revised our manuscript based on their comments, and feel the manuscript has improved. Please find below our point by point responses below.

We wanted to make one very important note to the reviewers and editor: Since our original simulations were done, the functions pomp *and* mif2 *in the POMP package have been modified on CRAN. These functions are required for the plug-n-play method that we consider. We have re-run all simulations based on the revised package functions. We note that this has changed the plug-n-play behaviour.*

Reviewer 2

This manuscript describes an interesting simulation study comparing 6 different methods of estimating the R0 coefficient (WP, secB, ID, IDEA, plug-n-play and fullBayes). The data are simulated via three different compartmental models, SIR, SEIR and SEAIR. Methods are intended to be tested both under the well-specified model and parameters and under the miss-specified ones. The quality of this work is the large range of methods tested, from the more classical and simplified models to the fully Bayesian ones. However, while the idea of comparing the performance of the methods is good and promising and the spectrum of methods compared is broad, the study and manuscript suffer from several weaknesses.

The biggest problem is a misunderstanding of two random duration variables involved in the epidemiological analysis of a pandemic: the infectious period and the serial interval. The first is the random length of time a subject remains infectious, the second is the random time between when the infector develops symptoms and when the infected develops symptoms in a chain of transmission (see for example: Zhou X-H, You C, et al, 2020, the Lancet). These two intervals are in general quite different in mean; for instance for COVID-19 infection the mean infectious period is around 8-10 days (He X, Lau EHY, et al 2020, Nature; Zhou X-H, You C, et al, 2020, the Lancet) while the mean serial interval is around 4-5 days (Nishiura et al 2020, IJID; Du et al, 2020, CDC; Zhou X-H, You C, et al, 2020, the Lancet). The mix-up between these two intervals (and distributions) is evident on page 4 when it says: "The serial distribution is the distribution of the random amount of time that an individual is infected..".

We first, for clarity, define serial interval, infection period and infectious period, as there seems to be some confusion in the comment above. The infection period is the total time infected, including the infectious and non-infectious periods. The infectious period is one stage of infection when the infected individual can transmit the pathogen. This stage can also be split into two, including an asymptomatic infectious period, and a symptomatic infectious period. The serial interval is the average time from symptom onset of the infected to symptom onset of all infectees of the infected individual. There is no misunderstanding of the infection period, infectious period and the serial interval by the authors. Please be reminded that individuals in the E and A classes have no symptoms. Only when an individual transitions into the I class do symptoms appear. Individuals in the I class are symptomatic and infectious. This is the general definition of the I class in Mathematical Epidemiology.

We refer the reviewer to [Ma J. Estimating epidemic exponential growth rate and basic reproduction number. Infect Dis Model. 2020;5:129-141. Published 2020 Jan 8. doi:10.1016/j.idm.2019.12.009]. This reference provides a clear explanation of the derivation of the serial interval, and shows how it relates to the infection and infectious periods for the SEIR and SIR models, respectively. We have added citations to this reference in the current version of the manuscript.

In the previous version of the manuscript, the only model that equates the infectious period and the serial interval is the SIR model. They are indeed the same quantity, $1/\gamma$.

For the SEIR model, the infection period and the serial interval are the same. The authors have never equated the serial interval and the infectious period for the SEIR model. The infection period and the serial interval are both given by $1/\alpha + 1\gamma$, which is the sum of the time in the exposed class and the infectious period. Consider an infected individual. The individual can infect someone at the beginning, middle, and end of their infectious period. Averaging the time of infection, and the time spent in the E class of each new infected (infectee) gives, $1/\alpha + 1/\gamma$.

The previous version of our manuscript had an error in the serial interval calculation for the SEAIR model. For the SEAIR model, the infectious period $= 1/\alpha + 1/\gamma$, the infection period $= 1/\alpha + 1/\rho + 1/\gamma$, and the serial interval is given by $1/\alpha + 1/\gamma$. Thus, the serial interval for the SEAIR model is the same as the serial interval for the SEIR model. Additionally, for the SEAIR model, the magnitude of the serial interval is less than the duration of the infection period. We have corrected the error in the current version of the manuscript.

This inaccuracy has consequences for the simulation study. In fact, data generated according SIR model of parameters beta and gamma have by construction mean infectious period of 1/gamma (fixed at 5 days for simulations). The problem arises when methods adopted for R0 estimation depend on the serial interval distribution, instead of the infectious period distribution, which is the case of the WP (White and Pagano 2007), ID and IDEA (Fisman 2013). In these cases models will not be well specified even when authors present them as being so. This can explain why in Fig 5, for example, WP, ID and IDEA methods (lines 1,3 and 4) seem to perform better when the gamma parameter is incorrect (right panel) than when it is correct (left panel). And comparing Fig 5 and 6 for the same methods, performance is improved when the model is miss-specified (R0 estimated assuming SIR with SEIR data). The authors need to address this point first.

Please see the reply to the comment above. Estimators that are based on the SIR model have, by definition, equality between the serial interval and the infectious period. If there is an exposed period, or an asymptomatic infectious period to the disease, application of a method based on an SIR model is an example of misspecification. If incorrect parameters are used in the estimator, it may perform better in a misspecified setting, but one error in parameter assumption would be accommodating the error in data structure. It is thus necessary to use a suite of estimator rather than rely on one. A sensitivity analysis is also always good to perform so that confidence in R_0 estimates can be gained. This is what we recommend in our manuscript.

A second point is inherent in the design of the simulation and the presentation of the results. The data are indeed simulated under a single choice of parameters, which may not be sufficient to draw general conclusions. Here, the parameters are chosen with respect to a given infection (influenza). It seems to me that adding other parameter choices would add value to the study. In addition, attention should again be paid to the fact that the gamma parameter do refer to the distribution of the infection period and not to the distribution of the serial interval.

We have revised our simulation study. We now have two influenza examples, and one COVID-19 example. Each are simulated under SIR, SEIR, and SEAIR modelling frameworks. We have also added an example using Canadian COVID-19 data.

The results are presented by boxplots, which is a good idea. However, on the one hand, some graphs are repeated several times (e.g. the WP case (SD = exp mean 5/7) with the SIR data is repeated 3 times in fig 2, 3 and 5), and I believe that a way could be found to avoid this. On the other hand, the results should also be presented numerically in tables, with for each setting the specification of the bias and variability of the simulated results at the inflection point, or with a summary of both (mean square error).

To not overwhelm the reader with all of the different example diseases, different simulations, and the real world COVID-19 case study, we have chosen to show results from a subset of our study in the main text. We have also modified our presentation to focus on mean-squared error (MSE) rather than show many box plots in the main manuscript. We have repeated the MSE plots in the Supplementary Material, but we also include all boxplots in the Supplementary Material as well.
There are many examples in the current manuscript. We have taken much time to add Tables for all of our results, as requested by the reviewer. These are included in the Supplementary Material as they are very large. They can also be difficult to read to see trends in results compared to, for example, MSE and boxplots, so they are not included in the main text of the manuscript.

An application to real data would also be interesting, in order to see how different R0 estimations the considered methods can produce on observed incidence data. I would personally be interested in seeing these results for COVID-19 outbreak.

We have included an application to real-world weekly COVID-19 data. The new example considers Canada as a whole, and also the three largest provinces (by population size), British Columbia, Ontario and Quebec. The new example is presented in the main manuscript. We note that the R_0 estimates using the real world data are presented using case data pre- and post-public health lockdown implementation. However, we only consider data after community transmission in Canada is confirmed, and only a short time after public health mitigation was imposed, to ensure that the estimates are not drastically affected by changes in the transmission rate as a result of the lockdown effects.

Finally, a thorough review of the English language is necessary.

We have revised the manuscript for grammatical errors.

Specific points:

- Page 2, line 26. ....”serial interval, infectious period... “. Please define all quantities when they are introduced

  We have checked our manuscript so that definitions appear at first introduction in the main manuscript.

- Page 3, line 74. Here gamma is set to 1/3, while in the Result section it is set to 1/5 (or 7/5 with weekly data).

  We have revised our simulation study so this statement no longer appears in the manuscript.

- Page 3, line 100. “ODE epidemiological model “. Please define

  ODE refers to ordinary differential equation. This definition is in the manuscript.

- Page 12, line 391: “Note that here the mean of the serial distribution was incorrect by only two days....”. Here authors don’t comment the fact that performance is better with the wrong serial distribution (see my comment above). In addition the amount of miss-specification (2 days) is chosen by the authors and they can modify it if it seems not enough to show some effect. I recommend testing a range of parameter choices.

  The revised manuscript includes parameter sets that are influenza-like, and covid-like.

- Page 15, line 490-91. “Asymptomatic infected (infected, no symptoms, not infection)”. Replace with : (infected, no symptoms, infection)

  We have revised the manuscript to clarify that E = infected, no symptoms, not infectious; A = infected, no symptoms, infectious; I = infected, symptomatic, infectious.

Reviewer 1:

In “Estimating the basic reproduction number at the beginning of an outbreak under incomplete data” by Boonpatcharanon and colleagues, different methods to estimate R0, the basic reproduction number, are compared considering the first 100 days of an epidemic. The authors apply frequentist and Bayesian approaches to estimate R0 under three different infection models: SIR, SEIR and SEAIR. These models differ in the allowed transitions between states of individuals (susceptible, exposed, (asymptomatic/symptomatic) infected, recovered). The authors conclude with a recommendation but also highlight that it always depend on the data which approach to choose; they recommend sensitivity analyses.

The aim of the study is described and motivated. The manuscript is well written. However, there are some issues the authors should consider to facilitate the readability of the manuscript.

Major issues:

1. “Incomplete data” sounds like missing data related to counts of, e.g., infected individuals. Should “incomplete” also comprise incomplete knowledge/information on transmission and course of infection? Please clarify (in the manuscript and probably in the title). Additionally, please add information on the required (observed) data underlying the R0 estimation/calculation.

   We have changed the title of the manuscript.

2. Please provide real data applications to support the assumptions in the simulation study and to illustrate the investigated methods on real data. For influenza, weekly case reports are published for several seasons, for example by the ECDC (European Centre for Disease Prevention and Control), the Government of Canada or the CDC (Centers for Disease Control and Prevention).

   Weekly case reports are common for influenza. The ECDC, US CDC and the Government of Canada all produce weekly reports (https://www.cdc.gov/flu/weekly/index.htm, https://www.canada.ca/en/public-health/services/diseases/flu-influenza/influenza-surveillance/weekly-influenza-reports.html). We have therefore chosen to work with weekly reported data from our simulations. We have added references to these weekly reports in the Introduction.

   In the revised manuscript, we have included a new study on a covid-like parameter set, and weekly COVID-19 reported data in Canada

3. Please provide the (documented) source code for the investigation to redo the analysis (including data simulation and figure/table preparation).

   We provide method implementation in the Supplementary Material. Source code and data examples are provided in an online github repository found at https://github.com/hannajankowski/$R0_e stimators_d ata$.

4. Introduction:

   (a) Could the authors elaborate more on data misspecification? Maybe through an own paragraph including examples of misspecifications and their possible influence on the R0 estimates? This issue is related to the reliability of an R0 estimation in the epidemic situation itself. The benefit/value of the R0 estimation depends heavily on the population under investigation, i.e. whether this population is a random sample of the total population or a for the total population not representative subpopulation (i.e. comprising, e.g., more or fewer infected individuals or different transmission probabilities than in the total population). Issues to be considered are for example the test strategy (which individuals are tested or must provide a test result; related to the number of unreported cases) and the test quality (reliable test results).

   Most real-time R_0 estimators rely on model approximation, and often this is derived from the SIR compartmental model. Our investigation focuses on what happens to these estimators when the data behaves according to one of the SIR, SEIR, and SEAIR compartmental models. This is the focus of our misspecification investigation. We also consider misspecification of the serial interval in R_0 estimators which assume this is known. The type of misspecification discussed above by the referee is therefore outside the scope of this investigation. However, we have revised our introduction to make our objectives and scope more clear.

   (b) As the study is about the early stage of an epidemic (first 15 weeks), could the authors additionally include this time frame into the considerations about misspecification? In case of a "new" disease, the knowledge on which, e.g., R0 estimation is based is limited in the early days. Could the authors please highlight the important issues unique to the beginning of an epidemic/pandemic – compared to the subsequent time? Besides in the beginning of an epidemic, is it also possible to consider a time point within an epidemic with a very low number of infected individuals, e.g. between two waves or two seasons (in case of seasonality as for influenza)? Please clarify "early stage". Please add a motivation for considering only the first 15 weeks.

   In the section describing the simulations, we have added a discussion on what we mean by the early stage. Again, we note that this is determined by the inflection point of the epidemic curve. In all settings considered here the inflection point occurs much earlier than 15 weeks, which is the full time period studied (this was chosen as it is more than sufficient to consider the early stage of an outbreak). Since the point of inflection is unknown at the beginning of an outbreak, we consider all 15 weeks, in essence studying what the referee refers to as "misspecification of the early stage". We also note that we are studying the basic reproduction number and not the effective reproduction number, thus we do not consider more than a single wave. The latter is of course also of great interest, it is simply out of the scope of this study.

   (c) Please add a motivation for the decision to consider SIR, SEIR and SEAIR only.

   Many R_0 estimators have been constructed to work within a Susceptible-Infectious-Recovered (SIR) disease modelling framework. Infectious diseases, however, can include periods of infection that are not infectious. The infectious period can also be split into various stages of asymptomatic and symptomatic infection, which ultimately affect the case reporting rate to public health. Therefore, methods that are based on the

SIR modelling framework can project erroneous estimates of R_0, and differences in R_0 estimates may simply reflect poor estimator structure or application to data that has been misspecified. These aspects make it difficult to compare R_0 estimates to gain increased certainty. This motivates the consideration of SIR, SEIR and SEAIR models. These models provide the basis for all infectious disease models. We therefore consider these three only. We have revised the manuscript to clarify this point.

5. Materials and Methods:

   (a) Please provide the underlying assumptions related to the data for the investigation (i.e. no unobserved infections, no reporting delay, . . .).

   The Discussion includes the text "In our current study we have assumed perfect data with no unobserved infections, no reporting delay, and no data collection bias. These issues are intuitively expected to affect R_0 estimates. We venture to continue our study of R_0 estimation while considering these aspects in our epidemiological data sets. ".

   (b) Please include a section about the simulation study. The approach description should not be part of the result section and the parameter choice should not be part of the method description. Please aggregate.

   We have modified the manuscript to add more detail regarding the simulation study. Detail regarding the method is in the Methods section. The epidemic curves produced by the simulations are in the Results section.

   (c) In some parts, methods are provided in the results section and vice versa. Please check and separate.

   We have modified the manuscript.

   (d) Lines 64-77: Please provide a supporting figure for illustration, if possible. Furthermore, please consider the inclusion of Table 1 in this figure and, if possible, remove Table 1.

   A flow diagram of the SIR, SEIR and SEAIR models has been added to the Appendix. The epidemic computer simulations are stochastic, agent-based simulations. Depending on the epidemic model, times of transition from E to I, E to A, A to I, I to R are assigned to individual infecteds when they enter the E, A, or I classes. The model records the number of individuals in the S, E, A, I, R classes at every time point. The epidemic data is then compiled into weekly case report data. The computer simulation is written in C++. The C++ simulation code is not included in the manuscript. It is merely used to generate data, which can be done using many different methods. The solution to the system of ordinary differential equations coincides with the mean of the simulation datasets (see black line in Figure 1 in the main manuscript). This shows that the simulations agree with the ODE system.

   Tables related to the simulation study are included in the main text to aid in understanding of the simulation method.

   (e) Line 97: Please introduce the methods briefly (including the reference to the respective subsection) and provide the abbreviations used throughout the manuscript. Then, refer to Table 3. Otherwise, the subsequent sections cannot be followed easily.

   We have revised the manuscript.

   (f) Line 103: Please consider to describe "serial distribution" earlier in the manuscript because it was already used earlier. Suggestion: Provide a section with definitions needed for the models (SIR, SEIR, SEAIR). Furthermore, please consider a summarisation of all parameters that are set to some selected values in the investigation. A table (or subheadings after re-ordering) might help.

   We have added a subsection on the serial distribution. We have revised the manuscript noting the first instance of the each abbreviation. We have added a table of parameters and refer to each example in the main text given the nomenclature in the tables.

   (g) Part 0.2.1

      i. Please check notations and definitions. For example:

         A. Line 133: "or" instead of ", or,".

B. Please unify kappa and k.

We have modified the manuscript.

C. Line 135: "both" does not fit to "the method", which is one method. Please check.

We have modified the manuscript.

D. Lines 137/138: Please add the origin for "number of days or weeks".

This is unclear. The line states $t_0 = 0$*, which is the origin.*

E. Line 139: Please clarify $min(\kappa, t)$. What is t?

We are confused by this request, as it is fair to assume that the reader understands the minimum function and that t represents time. The formula is for $\mu(t)$ *and in the formula the value* $\min(\kappa, t)$ *must be evaluated.*

F. Line 139: Please clarify the relation between $I(t–t_j)$ and $I(t)$, if there is one, otherwise please define I(time difference / interval).

We again find ourselves confused by the request, as $t - t_j$ *is a specific time and thus* $I(t - t_j)$ *and the entire formula is well-defined. Please see the work of White LF, Pagano M (2008) A likelihood-based method for real-time estimation of the serial interval and reproductive number of an epidemic. Statistics in Medicine 27: 2999–3016. Additionally,* $t - t_j$ *is common notation in the mathematical sciences.*

G. Line 159: Please clarify "built-in alternative optimisation". Where is it "built-in"?

After revisions, this line no longer appears in the manuscript.

ii. Please provide $p(t_j)$ for all models.

We have revised the paragraph. However, $p(t_j)$ *is too complex to report for SEIR and SEAIR models, as it would not have a nice closed-form expression. We feel that adding these formulas would detract from ease of reading.*

iii. Lines 149/150: Please explain the limitation.

There is no limitation referred to in lines 149/150. These lines reiterate that the WP method should be applied early in the epidemic as it assumes that the population size is constant.

iv. Lines 150: Please provide the section reference for the simulations.

The simulations are described in more detail in a separate section.

v. Instability issues (lines 156-165):

A. Might the instability be an indicator for non-adequateness of the applied method?

Not at all. The implementation in Obadia et al uses the general R optimization function called 'optim' which does not perform well to find the MLE when the likelihood is flat, as is the case for our data sets. This is why we based our implementation on a different method.

B. Please consider to include the observed instability issues in the result section to clearly separate methods and results (introduction of new subsection headings might help). Is it possible to quantify these issues?

The instability issues cannot be quantified; the optim function used by Obadia et al simply fails to provide a result for certain data sets. Secondly, we believe that discussion of implementation is more appropriate as part of discussion of the method.

C. Was the implementation of the grid search approach in comparison to the original implementation validated? If so, how?

We checked validity both visually (by plotting the likelihood function), by refining the grid, and by using also a stochastic search algorithm. This approach allowed us to determine the accuracy of the method under different conditions. The grid search does not guarantee to find the maximum, however, it is guaranteed to find the maximum on the grid. The inclined user can refine the grid as per their personal preference.

(h) Part 0.2.2:

i. As long intervals without new infections are problematic for this approach, this approach might be better suited for situations after the start of a new "wave" with rapidly increasing numbers of newly detected infections. Did the authors investigated scenarios, in which the numbers only increased slowly, or were the scenarios adapted to this method? In the latter case, a comparison in a non-adequate scenario would be of interest to guide future method applications. Especially in the beginning of a pandemic, such situations might occur.

The focus of the manuscript is on the basic reproduction number $R0$, not the effective reproduction number. We therefore do not consider new waves of infection as this would be inappropriate. As stated in the manuscript, the method will not work (ie. a numerical result is impossible) when there are zero cases in an interval. The goal of the manuscript is to compare existing methods, and not to create new ones. Therefore, the question of the referee is outside the scope of the manuscript.

ii. Lines 214/215: Please state the adaptations in more detail. Was the implementation in comparison to the original implementation validated? If so, how?

Validation is completely unnecessary in this case. The seqB method is Bayesian and we simply return the posterior mean and not the posterior mode due to preference. Our code returns also the full posterior distribution, and the interested user can calculate the posterior mode if they so wish.

(i) Part 0.2.3:

i. Lines 233/234: Please clarify "beginning of an outbreak". The authors state that the number of infectious individuals rapidly decreases in the beginning, but in the beginning of a new disease few individuals are infected/infectious and the number of infected/infectious people increase. Otherwise, I would expect that R0 is overestimated as the estimate does not decrease fast enough. Please clarify.

Thank you for pointing this out: we indeed meant to write "increase", and have changed this in the manuscript. Beginning of an outbreak means early on, and certainly before the inflection point.

ii. Lines 244/245: Please provide a reference to the specifications of the misspecification.

Misspecification is a broad and widely used term which means that the true model differs from the statistical model used in estimation. We have re-written some parts of the introduction so that what we are studying is more clear. However, we know of no appropriate references to add.

(j) Part 0.2.4:

i. Please provide (throughout the manuscript) names of R packages besides the reference.

We have added reference to R0 and pomp R packages in the main text.

ii. Line 275: Please explain "particle".

We have re-written this section.

iii. Equation after line 279: $R0$ is probably not a single value as δ_t is probably a sequence. Please check and adapt, if necessary.

We have clarified that Δt is a fixed value, not a sequence.

iv. Line 280: Please clarify where "regardless of the epidemiological model" relates to (and what is model-dependent).

We have removed this line.

v. Line 282: Please check the reference to the appendix. Appendix 1.3 is "Least square estimation for the IDEA method". Please provide more comments in the source code (Appendix 1.4) and please check line breaks to facilitate reading.

Source code and data examples are provided at $https://github.com/hannajankowski/R0_estimators_data$.

(k) Part 0.2.5:

i. Line 292: Please provide the respective simplifications in the subsequent derivations.

We have provided the derivation for the most complicated case. For the simpler cases, the reader can ignore information to pertaining to the E, and A classes, given an SIR or SEIR model.

ii. Lines 294/295: Please describe m more clearly. Please explain additionally (besides the equation) m_j in words. Definition of m0 should be provided with the definition of m_j.

Modified as requested. m_0 has not been added though as it is not in the vector, please note the subscripts on the sum.

iii. Line 295: Please clarify "epidemic" and "much more information".

This is what is done in subsequent lines, although we have added some clarifications here.

iv. Lines 296/297: Please check the conditions for i.

These are correct as written.

v. Lines 299/300: What is the impact, if an individual needs more than one week to recover? What is the motivation for one week? Please add.

To implement the method, we had to assume that all people who are infected in week j will recover in week j+1. Given the behaviour of influenza infections (especially given the short infection period (less than half a week)) this is a reasonable assumption. This is also reasonable for COVID-19.

vi. Line 334: "obtained" instead of "obtain".

Changed.

6. Results:

(a) Lines 351-353: Please additionally consider the case that the population studied is not a random sample of the target population. Alternatively, please clearly state (when defining the study design) the assumption that the populations studied is a random sample and discuss this assumption as limitation.

We consider a general population. A random sample from the general population considered here can be representative of any population. This comment is outside the scope of our study. The question that we are studying is 'can these methods estimate the parameters of the target population?'. We consider a fixed target population and use this population to compare all R_0 estimators.

(b) Lines 377 to 380: Does the results change if the other methods are also only applied to the subset of samples? Please comment.

The proportions are small and thus do not affect the results.

(c) Lines 380/381: Please define bias and variability. Did the authors also consider a joint measure of bias and variability?

We have added definitions of bias and variability. As to the second point, this is why we report the boxplots, as these provide very good visual summary to the reader of the interplay between bias and variability, skewness, etc. without overwhelming the reader with numerical summaries.

(d) Line 382: A figure cannot study. Please rephrase throughout the manuscript.

We have revised the text.

(e) Please consider to add further subsections to provide more guidance to the reader.

We have considered all reviewer comments and have revised the manuscript to be more clear in presentation.

(f) Line 405: Computation time is provided but the related section follows later-on. Please reorder.

We have moved the table.

(g) Part 1.1: Could the authors please provide computational aspects for all models?

We have added a sentence to comment on this.

7. Discussion:

(a) Please provide a paragraph about strength and limitations.

The entire study is about strengths and limitations for each method. We outline strengths and limitations in the description of each method, and we provide a comparison between all estimators in the results section.

(b) Please compare the results (at least in parts) with other studies.

We are not aware of any other study that considers model misspecification in a similar fashion.

8. Abbreviations, parameter, model names, methods names and other short forms:

   (a) Please introduce all in the main part of the manuscript. E.g. ODE, MCMC, IID, S0, I0, S, I, S(t), SD, ... are missing.

   We have reviewed the manuscript for consistency.

   (b) Please check the usage for consistency, e.g. S versus $S(t)$.

   We have reviewed the manuscript for consistency.

   (c) Please state which parameter are 0 at $t = 0$.

   No model parameters (β, γ, α, ρ) are 0 at $t = 0$. Parameters have constant values.

9. Figures:

   (a) Please provide axis titles at the respective axis and not in the description.

   We have revised the figures and the text to aid in understanding of the axis labels.

   (b) In case the legend only comprises one symbol/colour differing between figure panels, please consider providing this information as panel title above the respective plot panel. This also introduces shorter description.

   Please see point above.

   (c) Please introduce all abbreviations, parameter and model names in the figure description.

   We have reviewed figure descriptions.

   (d) In case of boxplots, please provide complete boxplots. In case of a needed zoomed-in boxplot, the complete one should be provided in the supplement.

   This would go against established best practice which requires that the axes be kept similar when comparing plots. However, we have added tables which include medians and standard deviations for all boxplots, which should sufficiently describe the results.

   (e) Please provide information in the description of the boxplots so that the reader is able to identify scenarios with misspecifications.

   We have included the simulation name in the Figure descriptions.

10. Tables:

    (a) Please introduce all abbreviations, parameter, model names and method names in the table description.

    We checked that sufficient information is given in all table descriptions.

    (b) Please provide a description that allows to understand the table without the part in the main manuscript where the table is cited for the first time.

    We checked that appropriate descriptions are given in all tables.

Minor issues:

1. Section numbering in the main part: Please remove the leading "0.". Please check the complete numbering and doubling of section headings, e.g. "Results" and "1. Results" and supporting information starts with 1.2.

   We have redone the mansucript in PLOS format, which does not allow section numbers.

2. Please consider to avoid "flu" and to use "influenza" throughout the manuscript.

   Changed.

3. Materials and methods:

   (a) Line 58: Please clarify "approximately".

   We are unsure about this request for clarification means.

   (b) Line 77: It should probably be $I(0) = 1$ (first round bracket is misplaced).

   This has been fixed.

   (c) Line 85: Please provide information on the meaning of "inflection" in lay terms (i.e. related to the course of infection/pandemic). *The inflection point refers to the point whereby the curve changes in concavity. We have added some description to the main text.*

   (d) Line 126: Please provide some additional information on the computer.

   We feel that information provided is sufficient for the reader.

   (e) Part 0.2.2:

   i. Equation after line 192: To stick to the notation throughout the manuscript, please consider replacing s by t, i.e. $S(t)$ and dt.

   We deliberately use s to facilitate reading of the equation as t (albeit with subscripts) appears as integral limits.

   ii. Line 194: Please consider to replace — by "given", i.e. "conditional distribution of $I(t_j + 1)$ given $I(t_j)$ and R_0". This would facilitate reading.

   We prefer to use the existing standard notation.

   iii. Line 196: Please introduce $N0$.
   N0 is the initial population size.

   (f) Part 0.2.3:

   i. Please introduce s and d.

   Added.

   ii. Line 230: Please delete "obvious".

   Completed.

   iii. Equation (4): Please consider to use additional brackets so that it is clear to which the sum sign belongs.

   Modified.

   iv. Lines 243/244: "However, ..." instead of "..., however.".

   This was a typo, and we have removed "however" entirely.

4. Figure 1:

(a) Lines 84/85: Please consider to remove parts of figure descriptions from the main text that should be part of the description accompanying the respective figure itself, i.e. below the figure panel(s).

The figure captions have been reviewed for clarity.

(b) Please introduce the meaning of "inflection".

The point of infection is the term used to identify the point in a curve where the concavity changes (from up to down, or down to up). The point of infection here is defined by the concavity of the infection curve. The description in the main text of the manuscript. Reiterating the definition of inflection point in figure captions will make the figure captions very lengthy, which detracts from readability of the important information related to the figures.

5. Table 2: Please clarify the meaning of Y_i (exponentially distributed with a mean of 1). Later-on, it is a mean of $1/\gamma$ (provided as an example). Or other natural numbers. Please consider a consistent notation.

Since Table 2 has been altered, this notation no longer appears.

6. Supporting information:

(a) a. Part 1.2:

i. Please provide references for the models and their chosen parametrisation.

Parameter values are informed by the literature. References are provided in the main text.

ii. Please introduce all parameter in more detail, even if they are introduced in the main text. Providing all definitions facilitates reading. The authors could consider to introduce a separate section within 1.2 for definitions. An alternative might be to provide the definitions in the main text, e.g. in a table.

The reader is referred to the appropriate sections in the manuscript. Reiterating description material detracts from readability.

(b) Part 1.3:

i. Please provide the partial derivatives and few more steps of the solving process.

We feel that this addition in unnecessary to understand the method.
