# Supplementary material for: Estimating the basic reproduction number at the beginning of an outbreak
Source: PLoS One. 2022 Jun 17;17(6):e0269306. doi: 10.1371/journal.pone.0269306 (PMC9205483; doi:10.1371/journal.pone.0269306)
Supplement: Attachment — Submitted filename: main_revrev.pdf [file pone.0269306.s004.pdf]

Response to reviewers for PONE-D-21-22343: "Estimating the basic reproduction number at the beginning of an outbreak"

Sawitree Boonpatcharanon, Jane Heffernan, Hanna Jankowski

April 18, 2022

Dear Editor and Reviewers,

We thank the Editor and both reviewers for their consideration and careful reading of our manuscript. Reviewer #2 is now completed satisfied with the manuscript and is not requesting a single change. Reviewer #1 has requested some very minor changes and has also provided some mild suggestions. We have carefully considered and addressed all comments. Detailed responses appear below.

1. If the authors have adequately addressed your comments raised in a previous round of review and you feel that this manuscript is now acceptable for publication, you may indicate that here to bypass the "Comments to the Author" section, enter your conflict of interest statement in the "Confidential to Editor" section, and submit your "Accept" recommendation.

   Reviewer #1: (No Response)

   Reviewer #2: All comments have been addressed

2. Is the manuscript technically sound, and do the data support the conclusions?

   The manuscript must describe a technically sound piece of scientific research with data that supports the conclusions. Experiments must have been conducted rigorously, with appropriate controls, replication, and sample sizes. The conclusions must be drawn appropriately based on the data presented.

   Reviewer #1: Yes

   Reviewer #2: Yes

3. Has the statistical analysis been performed appropriately and rigorously?

   Reviewer #1: Yes

   Reviewer #2: Yes

4. Have the authors made all data underlying the findings in their manuscript fully available?

   The PLOS Data policy requires authors to make all data underlying the findings described in their manuscript fully available without restriction, with rare exception (please refer to the Data Availability Statement in the manuscript PDF file). The data should be provided as part of the manuscript or its supporting information, or deposited to a public repository. For example, in addition to summary statistics, the data points behind means, medians and variance measures should be available. If there are restrictions on publicly sharing data—e.g. participant privacy or use of data from a third party—those must be specified.

   Reviewer #1: No

   Reviewer #2: Yes

   All data sets have been provided in a github repository which was shared with the reviewers. Reviewer #1 makes reference to the github repository in their comments, so we are slightly unsure why "no" was selected above.

5. Is the manuscript presented in an intelligible fashion and written in standard English?

   PLOS ONE does not copyedit accepted manuscripts, so the language in submitted articles must be clear, correct, and unambiguous. Any typographical or grammatical errors should be corrected at revision, so please note any specific errors here.

   Reviewer #1: Yes

   Reviewer #2: Yes

6. Review Comments to the Author

Please use the space provided to explain your answers to the questions above. You may also include additional comments for the author, including concerns about dual publication, research ethics, or publication ethics. (Please upload your review as an attachment if it exceeds 20,000 characters)

Reviewer #2: Thank you for having addressed all of my comments regarding the description of the method and presentation of the results. The manuscript is now easier to understand, thanks also to a revision of the English language.

Thank you kindly for your careful reading of our manuscript.

Reviewer #1: The authors have greatly improved their manuscript. The additional explanations facilitate reading very much.

However, some issues remain to be solved or should at least be considered:

We have considered all of the comments below, and have provided detailed responses

(a) Source code: For reasons of reproducibility, please make the complete source code including the C++ code available.

*We have included the output of the simulations on the github respository. The C++ code is also posted there. We note, however, that the C++ simulation has provided data, but **any** stochastic model can provide the data to be input into the estimators. The C++ code is not needed to use the estimators that we study here.*

(b) Abbreviations: a. Please introduce all abbreviations (e.g., SI in the text and IF in table 3). b. Please consider avoiding some abbreviations (e.g., SI or SD) for a better readability of the text. SI and SD are only used in some places and, hence, might be avoided. This also applies to IF in line 498.

We have revised the text so that SI appears as serial interval. SI is introduced in figure captions when the figure lists this in the legend. IF is included in the Table caption.

(c) Citations: a. Please introduce space between authors and "(Year)", e.g. "Anderson and May (1992)" or "Allen et al. (2008)". b. Please remove "[" and "]" if the citation is a real part of the text and not only a reference to the reference section. Examples: Lines 23/24 or line 54. The brackets should remain, e.g., in lines 80/81 or line 134.

The reference list is formatted as per the LATEX style file for PLOS One. We cannot modify the formatting, but are sure that any issues with formatting will be settled with the journal in the proof development stage.

(d) Reference to supplement: Could the authors please provide a more detailed reference to the supplement in the main text, e.g. "see supplemental section 1.1" or "Fig. 7"?

We have added specific references when appropriate.

(e) Supplement, lines 608-611: Please provide more calculation steps, such that the reader can follow the derivations more easily. This is not for understanding the method but for enabling an easy tracking of the derivations.

We assume that the reviewer means the Appendix and not the Supplement. We have added a few lines to the basic calculus derivation here.

(f) Please stick to one notation – either influenza 1 and influenza 2 or influenza one and influenza two (main part and supplement).

Influenza one and influenza two have been changed to influenza 1 and influenza 2 throughout.

(g) Abstract: Could the authors please add some results and a conclusion to the abstract?

This has been done.

(h) Introduction: The authors clarified "early stage". However, I would like to come back to a point I made on the previous manuscript version. The authors state, that they only consider a single wave. Does this imply that (i) the complete pandemic only runs for one wave, (ii) each wave is considered as a new pandemic or (iii) that only the first wave is considered in this manuscript? This question arises, as the authors seem to model influenza seasons as different pandemics (or as different waves). At least for me, a detail is missing. Is there a difference between "wave" and "season" in the application of methods? Please clarify.

*The reviewer's point is only relevant if we were studying the **effective** reproduction number (R_e), which we are not. We are studying the **basic** reproduction number, R_0. This has been clearly stated throughout the manuscript.*

*The basic reproduction number can only be determined when an infected is introduced into a totally susceptible population, which can only occur if there are no individuals in the population that have some pre-existing immunity. The **effective** reproduction number can be determined for the latter case mentioned by the reviewer. This is not what we study here. We study the basic reproduction number, and therefore are only concerned with the increase in infections early in the first wave (of one or many waves) of a pandemic/epidemic/outbreak. We do not model seasonal influenza, but model pandemic influenza, and all influenza parameter values are chosen from the pandemic influenza literature (as stated in the main manuscript).*

Yes, there is a difference between a wave, and a season. Waves can be produced due to public health interventions - as seen in COVID-19. Seasons are affected by changes in transmission by season. In the current manuscript, we do not consider waves or seasons, and only consider the early infection numbers for COVID-19 or pandemic influenza, which is the only time-frame within which the basic reproduction number applies.

(i) Methods:

- Language:
  - Line 105: "equal" instead of "equation"
  - Line 143: "for our models" or "for the models" instead of "for the our models"
  - Lines 156/157: Please rephrase "The main difficulty in estimation is that complete data is unavailable for the full epidemiological model is unavailable." Delete "is unavailable" at the end of the sentence or replace "for" by "if"?
  - Line 190: Please delete "is a".
  - Line 196: ")" missing after $t_{(j+1)}$.
  - Line 239: I would like to emphasize, that the word "given" instead of "—" would facilitate reading as it is provided in the text and not in a formula. Furthermore, there might be readers that are not so familiar with mathematical notations, but want to read the method section.
  - Line 353: "..." between "b+1" and "b+B" probably missing.
  - Line 357: "obtain" instead of "obtained"
  - Line 363: "obtained" instead of "obtain"
  - Lines 370-373: Is "..."necessary? *It is absolutely necessary since $m_i \neq m_{i-1} + 1$.*

  Thank you for your careful reading. All 'Language' items listed above have been corrected.

- Format: i. Lines 106-108: Please consider providing each equation in a separate line instead of in line with the remaining text. ii. Lines 153, 296, 348: Section number is missing. Please check throughout the manuscript.

  Our concern that separating these equations would detract from the flow of the manuscript by giving these equations too much weight. We have modified the script, however, and now the formulas fit more esthetically into the text. We have fixed the Section references. We remind the reviewer that Plos ONE does not allow for Section numbers.

- Definitions / settings: i. Line 105: Please consider "for all time points $t \geq 0$ instead of "for all $t \geq 0$". ii. Line 192: Please consider $t_0 = 0$ (beginning of the pandemic), $t_1, \ldots$" (or something similar) instead of "$t_0 = 0, t_1, \ldots$" to provide the time origin. iii. Equation between lines 237 and 238 as well as line 238: Above, $t \geq 0$ is time in the process. Maybe I am missing something, but I would expect an subscript at t as it is related to $I(t_(j+1))$ iv. Equation between lines 276 and 277: Probably, s_j is

similarly defined as t_j. Please state this briefly. v. Line 335: Please introduce theta (again). vi. Line 351: Please introduce B.

We have clarified definitions/settings in the main text when required. To clarify, we think that the reviewer is referring to R_t for point iii. R_t simplifies to the basic reproduction number at the beginning of the infection, as stated in the line below the equation. We are unclear to what the reviewer is referring to for point iv, but mention here that the equations in subsection ID and IDEA have been reviewed and are mathematically sound.

- Please consider providing the link to the github somewhere in the main manuscript.

  Done. Please see subsection Supplementary Material (currently just before Appendix).

(j) Results: a. A figure or lines cannot plot (e.g. lines 441-443). Information is, e.g., provided or indicated in a figure. Please rephrase and check throughout the manuscript. b. Line 449: The comma after the 9 is probably misplaced. Please check. c. Line 450, suggestion: Replace "5, 6" by "5 and 6" to be consistent with the previously provided numbers. d. Lines 502-506: Probably "if" instead of "when".

We have clarified and/or fixed these items in the Results section. For point d., we believe that "when" is appropriate.

(k) Discussion: a. Please provide a paragraph about strength and limitations (and approaches to mitigate them) of the investigation (not of the studied estimators) presented in this manuscript – maybe just by reordering of the paragraphs or by highlighting these issues. b. Line 549: In the discussion, results provided in the result section should be summarised and a reference to the supplementary material should not be necessary.

We are deeply confused by the referees request to add a paragraph about strength and limitations of the investigation but NOT of the studied estimators: This manuscript provides an investigation of the behaviour of estimators. What other investigation are we meant to discuss? We note that we have provided an appropriate discussion of strengths and limitations of our investigation in the Discussion section. We have summarized our results in the Discussion, highlighted what is important, and referred the reader to the Supplementary Material to point them to the proper place to find specific information.

(l) Figures (main part and supplement):

- In general: i. Please assure readability of all parts of the plot, including axes and legend. ii. Please provide axes names (including unit) on the respective axes and not only in the figure description below the plot. iii. Please avoid overlapping plot symbols in the legend. iv. Please introduce for each figure all used abbreviations, e.g. SI.

  (i) and (ii): The plots are most visible when they are larger; adding axes labels unnecessarily will make the plots smaller and thus less readable. The reviewer's request is therefore contradictory, and we cannot provide both. We strongly feel that stating that the x axis denotes time in our figures in the comments is more than sufficient; indeed, this is standard practice in the literature. (iii) We found three plots where the legend overlapped in the most minimal fashion. These have now been modified, and no further overlap was found. (iv) All abbreviations have been introduced as requested in point (b) of the referee.

- Additionally in Figure 1: The legends could be omitted, if the model is provided in the top of each column – similarly to the disease in front of the rows. This would facilitate reading.

  We thank the reviewer for this suggestion.

- Additionally in Figure 6: i. Suggestion: Providing the column information (Canada, provinces) above the columns might facilitate reading, especially in the presence of the axes names (see 12.a.ii). ii. Lines 517-519 should be part of the figure 6 description itself.

  We thank the reviewer for this suggestion. We have included lines 517-519 in the caption for Figure 6.

- Additionally in Figure 8: i. The legend could be omitted, if the data is provided in front of each row. ii. Please provide the prior distribution above each column of the plot.

  We thank the reviewer for this suggestion. The prior is for a four dimensional vector of parameters which we cannot plot in two dimensions.

(m) Tables (main part and supplement):

In general: Please introduce all abbreviations, e.g. SI. *Please see point (b) of the referee; this has been done.*

Table 1 (c): Please refer to (b) for tuple definition. *There is no mention of tuples in the referee's point (b). Indeed, we are not sure what "tuple" means here. Furthermore, the word "tuple" does not appear in Table 1 (c).*

Table 2: No reference for ID method available? *Added.*

Supplement: Please correct "... denotes a standard deviation great than..." to "denotes a standard deviation greater than". *Done.*
