# Supplementary material for: Estimating the basic reproduction number at the beginning of an outbreak
Source: PLoS One. 2022 Jun 17;17(6):e0269306. doi: 10.1371/journal.pone.0269306 (PMC9205483; doi:10.1371/journal.pone.0269306)
Supplement: Attachment — Submitted filename: reply_to_reviewer.pdf [file pone.0269306.s005.pdf]

Response to reviewers for PONE-D-21-22343: "Estimating the basic reproduction number at the beginning of an outbreak"

Sawitree Boonpatcharanon, Jane Heffernan, Hanna Jankowski

May 4, 2022

Dear Editor and Reviewers,

We thank the Editor and both reviewers for their consideration and careful reading of our manuscript. Reviewer #2 is now completed satisfied with the manuscript and is not requesting a single change. Reviewer #1 has requested two minor changes and we have implemented both. Detailed responses appear below.

1. If the authors have adequately addressed your comments raised in a previous round of review and you feel that this manuscript is now acceptable for publication, you may indicate that here to bypass the "Comments to the Author" section, enter your conflict of interest statement in the "Confidential to Editor" section, and submit your "Accept" recommendation.

   Reviewer 1: (No Response)

2. Is the manuscript technically sound, and do the data support the conclusions?

   The manuscript must describe a technically sound piece of scientific research with data that supports the conclusions. Experiments must have been conducted rigorously, with appropriate controls, replication, and sample sizes. The conclusions must be drawn appropriately based on the data presented.

   Reviewer 1: Yes

3. Has the statistical analysis been performed appropriately and rigorously?

   Reviewer 1: Yes

4. Have the authors made all data underlying the findings in their manuscript fully available?

   The PLOS Data policy requires authors to make all data underlying the findings described in their manuscript fully available without restriction, with rare exception (please refer to the Data Availability Statement in the manuscript PDF file). The data should be provided as part of the manuscript or its supporting information, or deposited to a public repository. For example, in addition to summary statistics, the data points behind means, medians and variance measures should be available. If there are restrictions on publicly sharing data—e.g. participant privacy or use of data from a third party—those must be specified.

   Reviewer 1: Yes

5. Is the manuscript presented in an intelligible fashion and written in standard English?

   PLOS ONE does not copyedit accepted manuscripts, so the language in submitted articles must be clear, correct, and unambiguous. Any typographical or grammatical errors should be corrected at revision, so please note any specific errors here.

   Reviewer 1: Yes

6. Review Comments to the Author

   Please use the space provided to explain your answers to the questions above. You may also include additional comments for the author, including concerns about dual publication, research ethics, or publication ethics. (Please upload your review as an attachment if it exceeds 20,000 characters)

   Reviewer 1: The authors have addressed almost all of my comments.

(a) An issue that was not addressed, yet, can be found in lines 410-425. There are still "influenza one" and "influenza two", although the authors stated that they have changed all to "influenza 1" and "influenza 2", respectively. Please adapt.

This has been done.

(b) Coming back to strength and limitations in the discussion, my wording was not clear. I am sorry for the inconvenience. My suggestion was to additionally provide a paragraph on strengths and limitations (and approaches to mitigate them) of the conducted study with respect to, e.g., selected scenarios and the selection of the investigated estimators. Additionally to the strengths and limitations of the investigated estimators. Maybe a simple reordering might be a solution for highlighting.

This has been done, please see lines 605-615 (last paragraph of the Conclusion section).
